# LION/web: a web-based ontology enrichment tool for lipidomic data analysis

**DOI:** 10.1093/gigascience/giz061

**Published:** 2019-05-29

**Authors:** Martijn R Molenaar, Aike Jeucken, Tsjerk A Wassenaar, Chris H A van de Lest, Jos F Brouwers, J Bernd Helms

**Affiliations:** 1 Department of Biochemistry and Cell Biology, Faculty of Veterinary Medicine, Utrecht University, Yalelaan 2, 3584 CM, Utrecht, The Netherlands; 2 Groningen Biomolecular Sciences and Biotechnology Institute and Zernike Institute for Advanced Materials, University of Groningen, Nijenborgh 7, 9747 AG Groningen, The Netherlands

**Keywords:** lipidomics, lipids, membrane biology, lipid ontology, LION, LION-term enrichment analysis, membrane biology, web-tool, data analysis, LION/web

## Abstract

**Background:**

A major challenge for lipidomic analyses is the handling of the large amounts of data and the translation of results to interpret the involvement of lipids in biological systems.

**Results:**

We built a new lipid ontology (LION) that associates >50,000 lipid species to biophysical, chemical, and cell biological features. By making use of enrichment algorithms, we used LION to develop a web-based interface (LION/web, www.lipidontology.com) that allows identification of lipid-associated terms in lipidomes. LION/web was validated by analyzing a lipidomic dataset derived from well-characterized sub-cellular fractions of RAW 264.7 macrophages. Comparison of isolated plasma membranes with the microsomal fraction showed a significant enrichment of relevant LION-terms including “plasma membrane", “headgroup with negative charge", "glycerophosphoserines", “above average bilayer thickness", and “below average lateral diffusion". A second validation was performed by analyzing the membrane fluidity of Chinese hamster ovary cells incubated with arachidonic acid. An increase in membrane fluidity was observed both experimentally by using pyrene decanoic acid and by using LION/web, showing significant enrichment of terms associated with high membrane fluidity ("above average", "very high", and "high lateral diffusion" and "below average transition temperature").

**Conclusions:**

The results demonstrate the functionality of LION/web, which is freely accessible in a platform-independent way.

## Background

The comprehensive study of lipids, also termed lipidomics, is gaining momentum. Instrumentation is becoming increasingly more sensitive, precise, and fast, and the use of lipidomics to address key questions in membrane biology has become widespread. As a result, datasets are rapidly increasing in terms of both size and complexity. Owing to a lack of methods to perform global and in-depth data mining, lipidomic research tends to focus on individual lipid classes or lipid species. A common approach in other “omics” disciplines to reduce complexity is the use of ontologies, e.g., Gene Ontology (GO) [1], Chemical Entities of Biological Interest ontology [2], combined with statistical tools to determine terms of interest. Although lipid structure is closely related to lipid function, it is currently impossible to associate properties of individual lipids with complex lipid mixtures of cellular lipidomes. Examples of biophysical properties that play an important role in membrane biology are numerous and include membrane thickness (e.g., as driving force in the sub-cellular localization of proteins [3]), membrane fluidity (e.g., regulating bacterial survival [4], membrane heterogeneity in cellular signaling [5]), intrinsic curvature (e.g., of lipids as key player in lipid droplet biogenesis [6, 7] or coat protein I [COPI] coat disassembly [8]), and net charge (e.g., of membranes as a determinant in lipid-protein interactions [9]). Here, we aim to provide a lipid ontology database and complementary enrichment analysis tool that (i) contains chemical and biophysical information of lipid species, (ii) is platform independent and compatible with routine mass spectrometry−based lipid analysis, (iii) can be used by researchers without computer programming skills, and (iv) is freely available to the scientific community.

## Findings

### Basic structure of LION

We constructed an ontology database called LION (File S1) that links >50,000 lipid species with 4 major branches: “lipid classification” (the LIPIDMAPS classification hierarchy [10]), “chemical and physical properties” (fatty acid length and unsaturation, headgroup charge, intrinsic curvature, membrane fluidity, bilayer thickness), “function", and “sub-cellular component” (predominant sub-cellular localization). The resulting database contains >250,000 connections (“edges”), providing a detailed system for in-depth annotation of lipids. An example of all LION-terms associated with a single phosphatidylserine (PS) lipid species, PS(34:2), is depicted in Fig. S1. We describe the construction of LION in more detail in the Methods section. All LION-terms, classification rules, and references are described in Data S1; all lipids currently supported by LION are described in Data S2.

### Addition of biophysical properties to LION

An important feature of LION is the association of lipid species with biophysical properties. We made use of experimental data (from 5 phospholipid classes and sphingomyelin) [11] and data (from 5 phospholipid classes) obtained by coarse-grain molecular dynamics simulation (CG-MD) [12], each providing distinct biophysical properties. These data were used to estimate the biophysical properties of all related lipids in the LION-database by multiple linear regression analysis.

The regression models were validated in 2 ways. First, we performed leave-one-out cross-validations (LOOCV) of all 3 models (Fig. S2A–C), showing satisfactory agreement between determined and predicted values. Second, we compared 2 properties closely associated with membrane fluidity: “transition temperature” (from experimental datasets) and “lateral diffusion” (from the CG-MD datasets) (Fig. S2D). As expected, lipids with low transition temperatures were predicted to have high lateral diffusion values at a defined simulation temperature and vice versa.

Subsequently, all numerical data points for each biophysical property were categorized into 5 predefined groups (“very low", “low", “average", “high", “very high”). We aimed to find group definitions with physiological relevance. Therefore, limits of each group were calculated on the basis of 4 mammalian lipidomics publications that served as a reference [13–16]. Using these group definitions, numerical values of all applicable lipid species present in LION were classified and connected to their respective LION-term (Fig. S2E).

### LION enrichment analysis and web-tool LION/web

**Figure 1:**
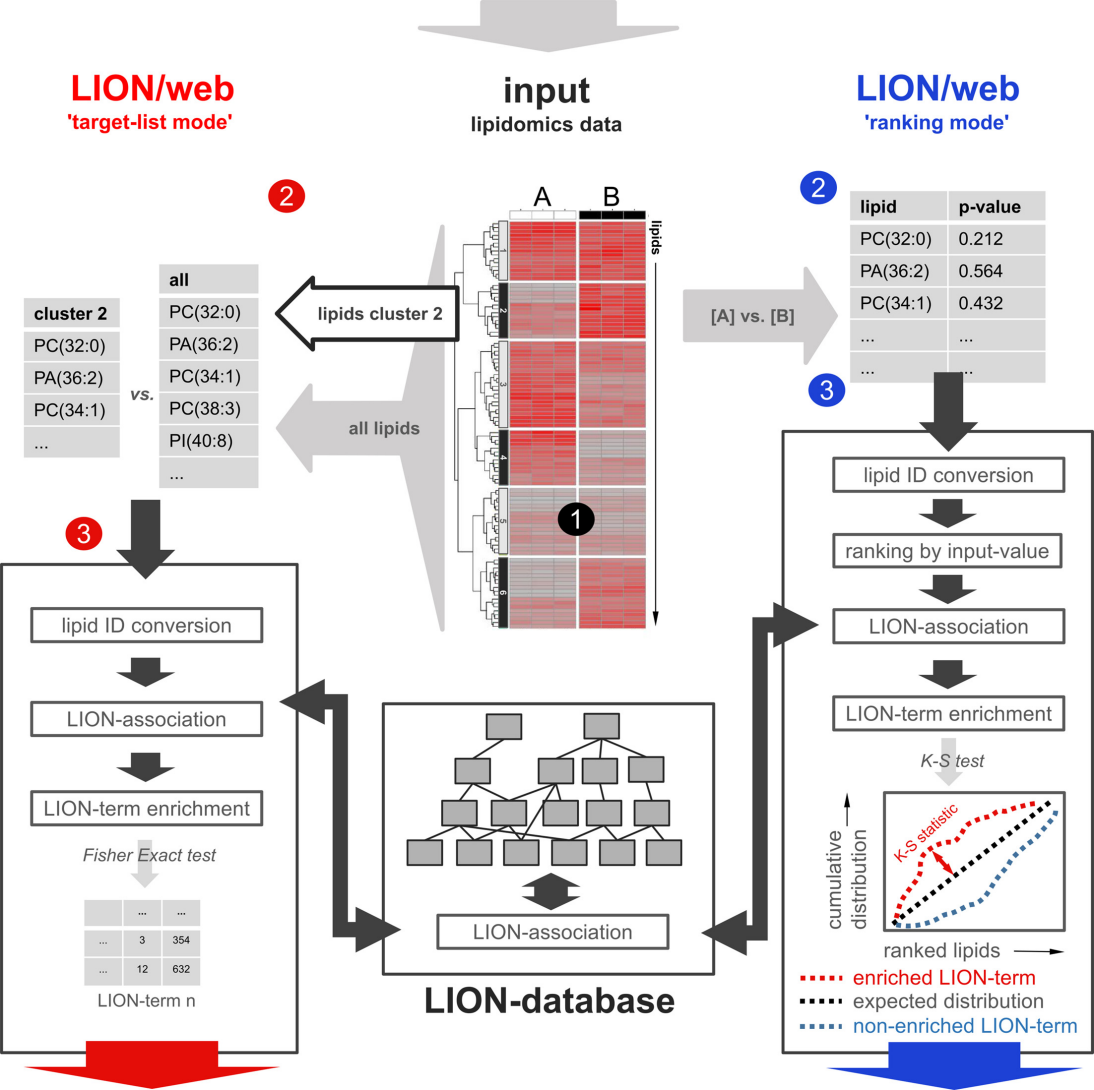
Enrichment analysis approaches supported by LION/web. A lipidomics dataset containing lipid identifiers and abundances derived from 2 or more conditions (1) can be processed by LION/web in 2 ways. In the “target-list mode” (left, red 2), a subset of lipids (e.g., derived from thresholding or clustering) is compared to the total set of lipids. After standardization of lipid nomenclature (red 3), applicable LION-terms are associated and assessed for enrichment in the subset by Fisher's exact statistics. In the “ranking mode", input lipids are ranked by numeric values (“local” statistics) (blue 2). After ranking, lipid nomenclature is standardized (blue 3). Applicable LION-terms are subsequently associated to the dataset and distributions are compared to a uniform distribution by “global” statistics (here, Kolmogorov-Smirnov [K-S] tests). Calculated *P*-values of LION-terms from both modes are corrected for multiple testing (Benjamini-Hochberg). PC: phosphatidylcholine; PA: phosphatidic acid; PI: phosphatidylinositol.

Next, we used LION as a basis to build an ontology enrichment tool that facilitates reduction of lipidome complexities in an unbiased manner. To this end, we made use of an adapted version of “topGO", an R package designed for enrichment analysis of GO-terms [17]. Subsequently, we designed a web-tool with R package Shiny (“LION/web" [18]) that offers an intuitive user interface and supports 2 major workflows (Fig. 1): enrichment analysis of a subset of lipids of interest (“target-list mode”) and enrichment analysis performed on a complete and ranked list of lipids (“ranking mode", referred to as “SAFE” and described in the context of genes [19]). A detailed step-by-step description of LION/web's workflow can be found in Note S1.

Analogous to GO enrichment approaches [1], which facilitate preselection of ontology subdomains or subsets of GO-terms (“GO-slims”), LION/web offers the option to limit analysis to specific LION-terms of interest. Furthermore, the web-tool allows removal of the most generic LION-term (the one with the highest hierarchy) if a related term contains the same subset of lipids. For example, the term “diacylglycerophosphocholines” might be associated with the same lipids as “glycerophosphocholines". With this option switched on, only the most specific term (“diacylglycerophosphocholines”) is included in the results.

### Performance of “target-list mode” by LION/web

**Figure 2:**
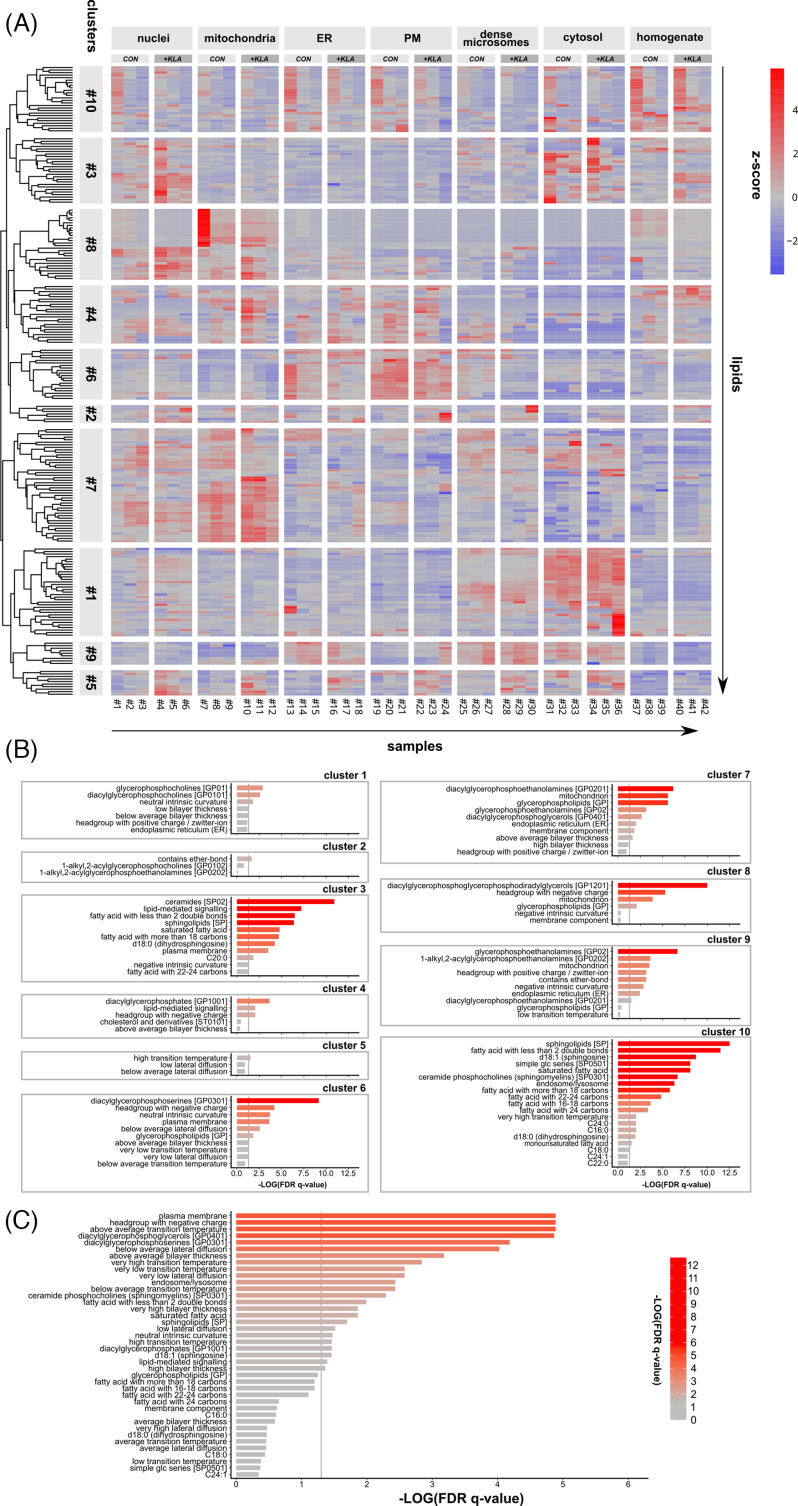
LION-term enrichment analysis of RAW 264.7 macrophages. (A) Heat map of scaled lipid amounts (z-score < 0: blue, z-score > 0: red) of sub-cellular lipidomics data [13] with samples on the x-axis and individual lipid species on the y-axis. Lipids were clustered into 10 groups by hierarchical clustering. (B) Enrichment analyses of all lipid clusters in the “target-list mode". For each cluster, the first *n* + 2 significant LION-terms are shown. (C) Enrichment analysis of plasma membrane (PM) vs endoplasmic reticulum (ER) fractions in the “ranking mode". The gray vertical lines indicate the cut-off value of significant enrichments (*q* < 0.05). Bar colors are scaled with the enrichment (−log *q*-values). FDR: false-discovery rate.

To test the functionality of LION/web, we made use of a previously published and well-characterized dataset containing lipidomics data from several sub-cellular fractions of RAW 264.7 macrophages, with or without toll-like receptor 4 (TLR4) activation by Kdo_2_-lipid A (KLA) [13] (see Methods for a direct link to the dataset). First, we renormalized the dataset by expressing all lipid species as fraction of the total amount of lipid per sample. Subsequently, the data were visualized by constructing a heat map graph (Fig. 2A). Lipid species were grouped into 10 clusters by hierarchical clustering. Each lipid cluster was subsequently analyzed by LION/web, which was able to reformat and match the vast majority (>97%) of the submitted lipids in the dataset. In the target-list mode, LION/web assesses the enrichment of LION-terms in a subset of lipids, as compared to all lipids in the experiment. For every cluster, lipids (Data S3) were entered as target-list and compared with the background list. Enrichment analysis of all 10 clusters resulted in ≥1 significant LION-term (Fig. 2B). The heat map showed that lipids present in clusters 7 and 8 were abundant in the mitochondrial fractions (Fig. 2A). In line with this observation, enrichment analyses of these clusters resulted in significant terms associated with this organelle (e.g., “diacylglycerophosphoetahnolamines", “mitochondrion", “diacylglycerophosphoglycerols", “headgroup with negative charge”). Similar results were obtained for cluster 6 (terms related to the plasma membrane), and to a lesser extent for cluster 9 (terms related to endoplasmic reticulum [ER]). Lipids in cluster 5 were more abundant in KLA-treated fractions and resulted in terms reported by LION/web that were associated with low membrane fluidity.

### Performance of “ranking mode” by LION/web

An alternative method to assess enrichment of LION-terms in LION/web is the ranking-mode. In the ranking-mode, all individual lipid species of 2 conditions are compared and ranked on the basis of a “local” statistic. This local statistic is any numeric value that associates individual (hence “local”) lipids with the provided conditions. LION/web supports 3 different local statistics: 1-tailed Welch 2-sample *t*-test *P*-values (comparison of 2 conditions); log_2_ fold-change values (comparison of 2 conditions), and 1-way ANOVA F-test *P*-values (comparison of >2 conditions). Subsequently, the distributions of all associated LION-terms over the ranked list are compared to uniform distributions by using 1-tailed Kolmogorov-Smirnov tests (“global” statistics, as full lipidomes are assessed). A LION-term is enriched when its associated lipids are higher ranked than expected by chance. To illustrate the ranking mode, we compared the isolated plasma membrane (PM) fraction (samples 19–21 from Fig. 2A) with the ER fraction (samples 13–15 from Fig. 2A) from non-stimulated macrophages using 1-tailed Welch 2-sample *t*-test *P*-values as local statistic. Subsequently, LION/web assessed all LION-terms for enrichment (Fig. 2C). In good agreement with current descriptions of the selected organelles [20, 21], significant enriched LION-terms included terms associated with chemical descriptions (e.g., “glycerophosphoserines", “headgroup with negative charge", “phosphosphingolipids”), biological features (“plasma membrane”), and biophysical properties (e.g., “above average bilayer thickness", “below average lateral diffusion", “very low lateral diffusion", “very high bilayer thickness", "neutral intrinsic curvature"). LION/web also reported the significant enrichment of “very high transition temperature", which is in line with the (very) low lateral diffusion terms (see also Fig. S2D). The term “very low transition temperature” was also reported to be significantly enriched. Inspection of the lipid species responsible for the LION-term “very low transition temperature” revealed the presence of lipids that all contain polyunsaturated fatty acids with ≥4 unsaturations. This may be a macrophage-specific phenomenon, related to their involvement in inflammation [22].

### Enrichment performance of chemical and biophysical LION-terms

**Figure 3:**
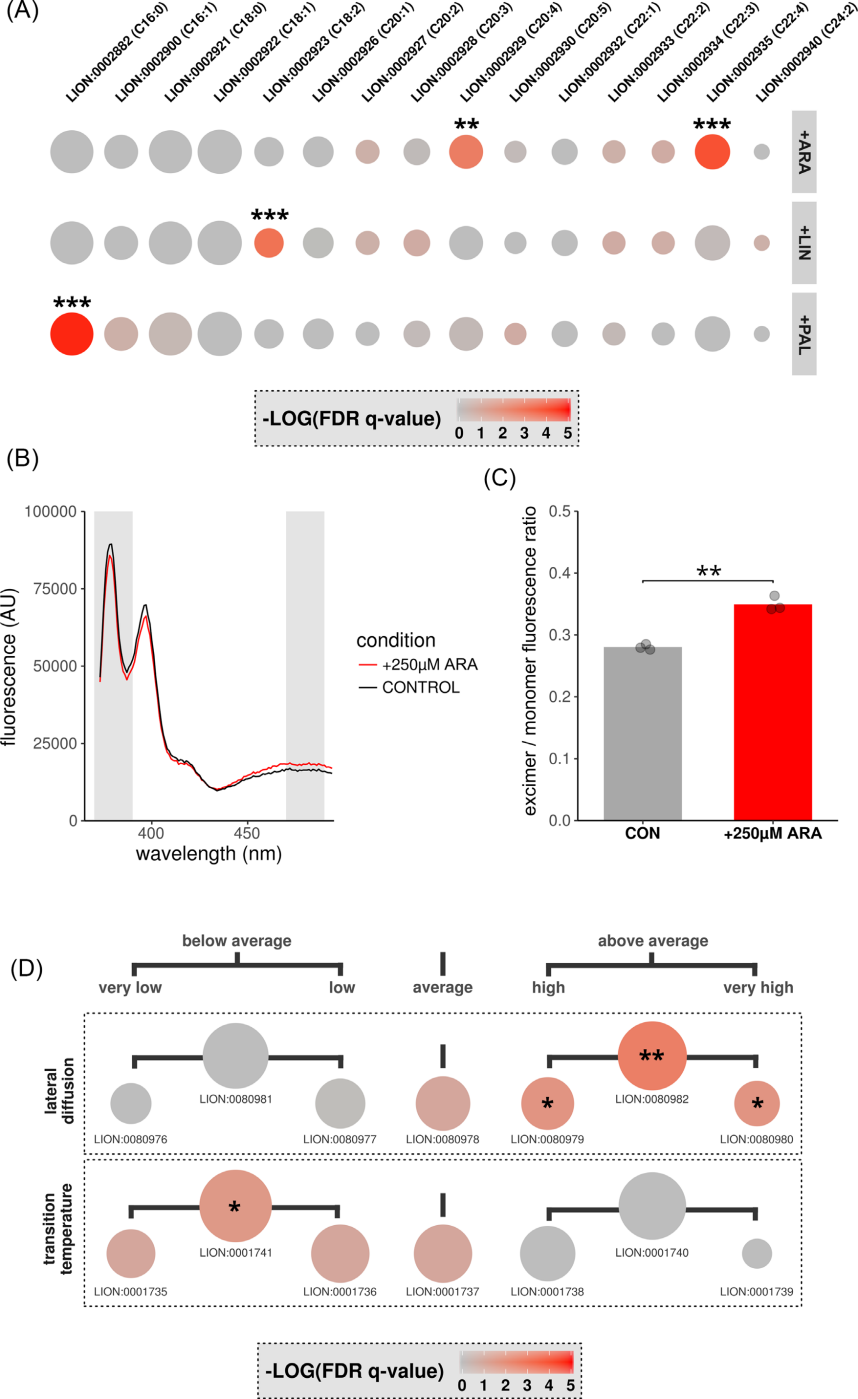
LION-term enrichment and membrane fluidity of CHO K1 cells. CHO K1 cells were incubated overnight with PAL, LIN, or ARA (100 μM) (A) or with ARA (250 μM) (B–D). All incubations were performed in triplicate. For control (CON) incubations, cells were incubated with fatty acid free–BSA. (A, D) After extraction and lipidomics profiling by LC-MS/MS, enrichment analyses of the conditions of interest vs control incubations were performed by LION/web of (A) LION-terms indicating the presence of selected fatty acids or (D) LION-terms indicating the degree of membrane fluidity. Dot sizes in the dot plots are scaled to the number of associated lipids; colors are scaled to the level of enrichment (–log *q*-values). (B, C) After incubation, fluorescence emission spectra of lysates containing pyrenedecanoic acid (PDA) were measured (B). Fluorescence spectra examples of either control (black) or ARA-stimulated lysates (red). Gray shades indicate monomer and excimer fluorescence filters. (C) Mean ratios (bar) and individual data points (dots) of excimer over monomer fluorescence (representative data of 3 independent experiments). Statistical significance was determined by Welch's 2-tailed *t*-test. (A, C, D) Asterisk indicates *P* or *q* < 0.05, double asterisk *P* or *q* < 0.01, and triple asterisk *P* or *q* < 0.001. FDR: false-discovery rate.

To further characterize the enrichment of chemical and biophysical properties by LION/web, we used 2 different experimental approaches. First, we investigated the enrichment of chemical features that can be easily incorporated into lipids. To this end, Chinese hamster ovary (CHO) K1 cells were incubated overnight in the presence of palmitic acid (PAL), linoleic acid (LIN), or arachidonic acid (ARA) complexed to bovine serum albumin (BSA). Subsequently, lipids were analyzed by liquid chromatography−tandem mass spectrometry (LC-MS/MS) and quantified. When available, we used MS/MS data to annotate lipids with their most abundant fatty acid composition. This level of annotation is important because it enables LION to link input lipids with terms associated with fatty acids (Data S4 and Fig. S3). Next, the web-tool was set to use the ranking mode and to limit analysis to LION-terms indicating the presence of fatty acids as lipid building blocks. LION/web reliably reported the significant enrichment of the respective fatty acid in the 3 different conditions (Fig. 3A and Data S5).

Second, to investigate the enrichment of biophysical LION-terms, we incubated CHO K1 cells with ARA. This procedure is known to increase membrane fluidity [ 23]. After incubation, the membrane fluidity properties of the samples were analyzed both experimentally and by LION/web. Membrane fluidity was experimentally assessed using pyrene decanoic acid (PDA) (Fig. 3B). This fluorescent probe can exist as monomer or excimer, resulting in a shift of its emission spectrum. The ratio of excimer over monomer fluorescence is proportional to the degree of membrane fluidity [24]. As expected, the ratio of excimer/monomer forms of PDA revealed a significant increase in membrane fluidity of lysates in the presence of ARA (Fig. 3C). For parallel LION/web analysis of membrane fluidity properties, lipids were extracted from the same samples and analyzed by LC-MS/MS (Data S6 and Fig. S4). LION contains 2 sets of terms associated with membrane fluidity: “transition temperature” and “lateral diffusion". Accordingly, LION/web was set to limit enrichment analyses to these sets, after which the lipidomic data were analyzed (ranking mode). In line with the experimentally measured increase in membrane fluidity, terms associated with high membrane fluidity ("above average", "very high", and "high lateral diffusion" and "below average transition temperature") were significantly enriched in cells that had been treated with ARA (Fig. 3D and Data S7).

## Discussion

Despite the quick growth of lipidomics and the rise of many tools to process raw data into lipid compositions [25], no automated pipeline to reduce complexity in lipidomic datasets using prior knowledge was yet available. Such a tool facilitates the generation of hypotheses, which is an important aim in many omics experiments. Here, we have presented a new ontology called LION and have used this ontology to build a web-based online LION-term enrichment tool suited to fulfill this need. In a single analysis, trends in complex lipidomic datasets can now be assessed in a standardized way. The web-tool ensures that the pipeline is accessible to users who are not familiar with programming languages.

Just like enrichment analysis approaches in other omics fields, LION-term enrichment analysis comes with specific strengths and limitations. The quality and coverage of the underlying ontology is of great importance. For LION, we aimed to support most commonly found lipid species in mammalian systems. In our examples, >85% of the input lipids could be matched to the ontology. Owing to the great diversity of lipidomes in different organisms, this coverage could be lower in user-provided datasets from non-mammalian systems. We hope to support LION's coverage of plant, bacterial, and yeast lipidomes better in the future. LION/web offers users several feedback options to keep track of missing annotations and to act specifically upon users' needs.

It is important to note that the enrichment of biophysical properties such as membrane fluidity, membrane thickness, and curvature cannot replace functional assays. More factors than lipids alone—e.g., protein composition, temperature—are playing important roles. Moreover, the effect of cholesterol is complex and depends on the interaction with other lipids. Therefore, the biophysical effects of cholesterol are not included. Also, the relative amounts of lipids in the described enrichment analysis methods are not taken into account: low-abundant lipids contribute equally to enrichment as their high-abundant counterparts.

This limitation can be circumvented by defining local statistics that takes abundances into account. This type of statistic will become more urgent when lipidomic analyses shift from mostly semi-quantitative to quantitative analyses in the future.

In summary, LION/web reveals changes in lipid patterns that allow researchers to study the complexity of lipidomes in a biological context. With future expansions of the LION database and of LION/web (also upon request of the scientific community), LION/web will increasingly successfully bridge the gap between lipidomics and cell biology.

## Methods

### Creation of lipid ontology (LION)

We built an ontology database that connects lipid species to the following 4 major branches: “lipid classification", “function", “cellular component", and “physical or chemical properties". For readability, a term is included at the top of each branch to indicate the nature of a LION-branch. These “category” terms are distinguished from other LION-terms with an ID containing the prefix “CAT”.

The classification system is based on the LIPIDMAPS classification [10]. LIPIDMAPS does not support lipid species with summed fatty acid. However, this extra layer is useful because it enables mapping when exact fatty acid compositions of measured lipids are not known. This concept is also used in the SwissLipids system [26]. Downstream, individual lipid species belonging to classes described in Data S1 were constructed as combinations of the following fatty acids: C12:0, C14:0, C14:1, C16:0, C16:1, C18:0, C18:1, C18:2; C18:3, C20:0, C20:1, C20:2, C20:3, C20:4, C20:5, C22:0, C22:1, C22:2, C22:3, C22:4, C22:5, C22:6, C24:0, C24:1, C24:2, C24:3, C24:4, C24:5, C24:6, C26:0; C26:1, C26:2, C26:3, C26:4, C26:5; C26:6, and C26:7. For sphingolipids, sphingosine (d18:1) and sphinganine (d18:0) were used as possible backbones. In the current version, LION does not distinguish between *sn*-positions. Fatty acids were ordered by chain length (low to high) and number of unsaturations (low to high). Altogether, LION contains ∼50,000 lipid species.

The branch “function” comprises 3 subcategories: “membrane component” (associated with lipids that are primarily regarded as a structural component of lipid bilayers), “lipid-mediated signaling” (lipids that have been implicated in signaling), and “lipid-storage” (lipids that are associated with storage, primarily in lipid droplets). In the category “cellular component", lipid classes that are enriched in particular cellular organelles are linked to their corresponding organelle terms [7, 20, 21]. The branch “physical or chemical properties” comprises a number of subcategories. First, a number of chemical descriptions (“contains fatty acid", “fatty acid unsaturation", “fatty acid length", and “type by bond”) was inferred from the species names. Second, data about “intrinsic curvature” [7, 27] were categorized into either negative, neutral, or positive curvature. Because data on the species level are limited, curvature was assumed to be predominantly headgroup-dependent and fatty acid composition was neglected. The third subcategory, “charge headgroup", was divided into 3 groups based on structural data: “negative", "positive/zwitter-ion", and “neutral” [26]. This last term also comprises lipids lacking a headgroup. The fourth subcategory in “physical or chemical properties” is “chain-melting transition temperature". This property is derived from a number of sources, comprehensively reviewed by Marsh [11]. This dataset covers a range of lipid classes in both glycerophospholipids (phosphatidylcholine [PC], phosphatidylethanolamine [PE], phosphatidylglycerol [PG], phosphatidic acid [PA], phosphatidylserine [PS]) and sphingolipids. We made use of multiple linear regression analysis with lipid class, fatty acid length, and unsaturation as predictors to facilitate data extrapolation to previously unreported lipid species. The obtained model (coefficients are available via Data S8) was validated by LOOCV. Briefly, 1 data point from the dataset was taken out, after which the model was rebuilt with the remaining points as training set. Subsequently, the selected data point was used as validation sample. This procedure was repeated for all the data points (Fig. S2C).

Ontologies contain categorical data and are not compatible with numeric values. Therefore, we classified chain-melting transition temperature values into 5 distinct categorical data groups: “very low", “low", “average", “high", or “very high". To define the limits of these intrinsic subjective groups, we used 4 previously reported datasets to serve as reference lipidomes [13–16]. From all reported lipids, the transition temperature was predicted by the model. The obtained transition temperature distribution was used to define the groups: the lowest 20% (first quintile) was classified as “very low", the second 20% (second quintile) as “low", etc. Subsequently, these limits were used to categorize all lipid species present in LION. Lipids with transition temperature values lower than the lowest limit were defined as “very low", whereas values higher than the highest limit were defined as “very high". A flow chart of this procedure is depicted in Fig. S2E.

In addition to these experimental data sets, we also used data [12] that were obtained by coarse-grain molecular dynamics simulation (Martini force field [28]) and which include the membrane properties “bilayer thickness” and “lateral diffusion". The dataset contains lipids from 5 common classes of glycerophospholipids (PC, PS, PG, PA, PE) but lacks sphingolipids and sterols. By definition, coarse-grained lipids represent a range of structures. So that the dataset could be used in the ontology system, names of coarse-grained lipids were translated into their representing counterparts. Subsequently, lipid properties were extrapolated to the entire database by multiple linear regression analysis models (with lipid class, fatty acid length, and unsaturation as predictors; coefficients are available via Data S8) and validated by LOOCV (Fig. S2A and B). We followed the same procedure as used for transition temperatures; extrapolated results for both properties were categorized into representative classes: “very low", “low", “average", “high", or “very high", based on values, predicted by our models, of the reference datasets [13–16].

The initial structure of LION was built with OBO-Edit v.2.3.1 [29] and formatted as an OBO file. Subsequently, custom R scripts connected specific terms with more general terms based on the described datasets. The entire ontology can be found as File S1.

### Implementation of enrichment analysis tool

To use LION with existing ontology enrichment tools, we used an adapted and generalized version of the Bioconductor R package topGO [17]. This version, called “topOnto", allows users to include ontologies other than those provided with the package. TopOnto's attached Perl script was used to convert the ontology file from OBO to SQLite format. Apart from this extra feature, the topOnto package provides the same functionality as the original version.

To perform the enrichment analysis, 2 statistical approaches are used. In the target-list mode, 1-tailed Fisher exact statistics are used to test enrichment. To this end, 2 × 2 contingency tables are constructed for every LION-term, containing the number of lipids associated and not associated with the given term for both the target-list and the background set, and analyzed. In the ranking mode, 1-tailed Kolmogorov-Smirnov tests are used as “global” statistics to assess enrichment of LION-terms over a ranked (by “local” statistics) list of lipids. For every LION-term, the cumulative distribution of associated lipids over the ranked list is compared with the uniform distribution. Enrichment is defined as over-representation of highly ranked lipids associated with the term. To rank input lipids, LION/web offers 3 different local statistics: *P*-values from 1-tailed Welch *t*-tests (2-condition comparison), log_2_ fold-change values (2-condition comparison), and *P*-values from 1-way ANOVA F-tests (>2 conditions comparison). Ranking direction (from high to low, or vice versa) is automatically updated after local statistic selection but can be set manually. In addition, users can use custom local statistics. In both modes, topGO's classic algorithm is selected [17]. After LION enrichment analysis, raw *P*-values are corrected for multiple testing (Benjamini-Hochberg). The R scripts were used to build the user-friendly web-based tool LION/web (Note S1) with R package Shiny. The application has been made available on the Shiny applications server as a free online tool, accessible through LION/web [18].

### Cell culture and preparation of fatty acid−albumin complexes

CHO K1 cells were cultured in Ham's F-12 medium (Thermo Fisher Scientific, Waltham, MA, USA) supplemented with 7.5% fetal bovine serum (Thermo Fisher Scientific, Waltham, MA, USA), 100 units/mL penicillin, and 100 μg/mL streptomycin (Thermo Fisher Scientific). Cells were grown in a humidified incubator at 37°C containing 5% CO_2_ and passaged twice a week. Stocks of 10 mM ARA, LIN, or PAL (all obtained from Sigma, St. Louis, MO, USA) were complexed to 2 mM fatty acid−free BSA (Sigma), filter-sterilized, and stored at −20°C. Control incubations without fatty acid contained equivalent amounts of fatty acid−free BSA. All experimental incubations were performed in plastic 6-well culture dishes (Corning, Tewksbury, MA, USA).

### Measuring membrane fluidity

After overnight incubation in the absence or presence of fatty acids (using fatty acid−free BSA or fatty acids coupled to BSA, respectively), cells were washed and scraped in phosphate-buffered saline. Cells were subsequently homogenized on ice with 26-gauge needles (BD Bioscience, San Jose, CA, USA). Homogenates (equivalent to 40,000 cells) were mixed 1:1 with the manufacturer's supplied dilution buffer (Membrane fluidity kit, Abcam, Cambridge, UK) in the absence (background) or presence of 5 μM PDA and transferred into a 96-well plate (black plastic with glass bottom, Greiner Bio-One, Frickenhausen, Germany). After 30 minutes of incubation at 37°C, fluorescence spectra (excitation at 360 nm, emission between 375–500 nm, 37°C) were measured with a temperature-controlled fluorescence microplate reader (CLARIOstar, BMG Labtech, Offenburg, Germany). Data were processed in R by expressing monomer (370–390 nm) and excimer (470–490 nm) as ratios of mean fluorescence after subtraction of background fluorescence (samples with cells but without PDA). Results were expressed as means. Differences were analyzed by 2-tailed Welch's *t*-tests.

### Lipidomics by LC-MS/MS

After incubation, lipids were extracted as described previously [30]. Lipid extracts were dried under nitrogen and dissolved in 100 μL chloroform/methanol (1:1) and injected (10 μL) on a hydrophilic interaction liquid chromatography (HILIC) column (2.6 μm HILIC 100 Å, 50 × 4.6 mm, Phenomenex, Torrance, CA, USA). Lipid classes were separated by gradient elution on an Infinity II 1290 UPLC (Agilent, Santa Clara, CA, USA). At a constant flow rate of 1 mL/min, acetonitrile/acetone (9:1, v/v) was used as solvent A. Solvent B consisted of a mixture of acetonitrile/H_2_O (7:3, v/v) with 10 mM ammonium formate. Both solvents contained 0.1% formic acid. The gradient was as follows (time in min, %B): (0, 0), (1, 50), (3, 50), (3.1, 100), (4, 100). Samples were injected without re-equilibration of the column. The column effluent was connected to a heated electrospray ionization source of an Orbitrap Fusion mass spectrometer (Thermo Scientific) operated at –3,600V in the negative ionization mode. Temperatures for the vaporizer and ion transfer tube were 275°C and 380°C, respectively. Full scan MS1 measurements in the mass range from 450 to 1,150 u were collected in the Orbitrap at a resolution of 120,000. Parallelized data-dependent MS2 experiments were done with higher-energy collisional dissociation fragmentation set at 30V, using the dual-stage linear ion trap to generate up to 30 spectra per second.

### Lipidomics data analysis

Acquired raw datafiles were converted to mzXML files by msConvert (part of ProteoWizard v3.0.913) [31] and processed with the R package xcms v2.99.3 [32]. After deisotoping, annotation of lipids was performed by matching measured MS-1 *m/z* values with theoretical *m/z* values. Lipids with the same or similar *m/z* values—e.g., bis(monoacylglycero)phosphate(38:4) and PG(38:4)—could be distinguished by differences in retention time (Figs S3 and S4). Lipid annotation containing individual fatty acids (extra column “most abundant isomer annotation” in Data S4) as used in Figs 2A and S3 was accomplished by examining MS-2 spectra. When MS-2 spectra were available for a given MS-1 peak, the most abundant fatty acid combination was used to annotate the lipid. The resulting experimental datasets, as well as the public RAW 264.7 macrophage dataset [13], were normalized by expressing all lipids as ratios of the sum of all intensities per sample. MetaboAnalyst 3.0 [33] was used to replace missing values (of the RAW 264.7 dataset) by half of the minimum positive value in the original data, and to perform principal component analysis (with Pareto scaling).

### Heat map, hierarchical cluster analysis, and LION-enrichment analyses

The heat map of the RAW 264.7 dataset was constructed after calculating *z*-scores for all lipids (all lipids were scaled to a mean of zero and a standard deviation of 1) using the R package pheatmap v1.0.10. Lipids were grouped by hierarchical clustering. The dendrogram of the lipids on the y-axis of the heat map used Euclidean distance as the similarity measure and was performed with complete linkage. The number of clusters was set to 10. Enrichment analysis of each of the 10 clusters was performed using the target-list mode with default settings.

Enrichment analyses used in Figs 2C and 3A and D were performed using the ranking mode, with 1-tailed Welch 2-sample *t*-test *P*-values as local statistics. The analysis for Fig. 2C was performed with default settings, whereas LION-terms to be considered were limited to all child-terms of “contains fatty acid” (CAT:0000100) for Fig. 3A and all child-terms of “chain-melting transition temperature” (CAT:0001734) and “lateral diffusion” (CAT:0080950) for Fig. 3D.

### Software and R packages

All R scripts were run with RStudio v1.0.153 (R v3.4.4) with the following packages: Shiny v1.1.1, visNetwork v2.0.1, data.table v1.10.4-2, GMD v0.3.3, igraph v1.0.1, reshape2 v1.4.2, ggplot2 v2.2.1, ggthemes v3.4.0, shinyTree v0.2.2, shinyWidgets v0.4.1, shinythemes v1.1.1, RSQLite v2.1.1, topOnto v0.99.0, pheatmap v1.0.10, and xcms v2.99.3 [32]. Perl scripts provided with the topOnto package were run with Perl v5.26.0. All figures were built in R and processed in Cytoscape v3.5.1 or Inkscape v0.92.2.

## Availability of source code and requirements

The source code of the web-tool is available via github.

Project name: LION-web

Project home page: https://github.com/martijnmolenaar/LION-web/

Operating system(s): platform independent

Programming language: R

License: GNU General Public License v3.0

RRID: SCR_017018

## Availability of supporting data and materials

The LION database (OBO format) and raw lipidomics data are available as Supplementary Data. The public RAW 264.7 macrophages dataset [13] is available on the journal's website [34]. The R package topOnto is available at [35], the associated R package containing the LION database in topOnto-friendly format at [36]. Snapshots of our code and other supporting data are available in the *GigaScience* repository, GigaDB [37].

## Additional files

**Additional Figure S1**. LION-terms associated with PS(34:2)

**Additional Figure S2**. Model validations of biophysical properties in LION

**Additional Figure S3**. Lipidomics of CHO K1 cells incubated with free fatty acids

**Additional Figure S4**. Lipidomics of CHO K1 cells incubated with ARA

**Supplementary Data 1**. XLSX-file containing all LION-terms excluding lipids with classification rules and sources

**Supplementary Data 2**. CSV file containing all lipids present in LION

**Supplementary Data 3**. CSV file with lipid clusters

**Supplementary Data 4**. CSV file with lipidomics dataset supporting Fig. 2D

**Supplementary Data 5**. CSV file with LION/web output values supporting Fig. 2D

**Supplementary Data 6**. CSV file with lipidomics dataset supporting Fig. 2A

**Supplementary Data 7**. CSV file with LION/web output values supporting Fig. 2A

**Supplementary Data 8**. XLSX file containing the coefficients of the biophysical models

**Supplementary Data 9**. CSV file with test set for lipid names conversion

**Supplementary File 1**. LION database in OBO format

giz061_GIGA-D-18-00357_Original_Submission

giz061_GIGA-D-18-00357_Revision_1

giz061_GIGA-D-18-00357_Revision_2

giz061_Response_to_Reviewer_Comments_Original_Submission

giz061_Response_to_Reviewer_Comments_Revision_1

giz061_Reviewer_1_Report_Original_Submission -- Ruth Welti 10/29/2018 Reviewed

giz061_Reviewer_1_Report_Revision_1 -- Ruth Welti, 3/5/2019 Reviewed

giz061_Reviewer_2_Report_Original_Submission -- Aleksander Andreyev 11/24/2018 Reviewed

giz061_Reviewer_2_Report_Revision_1 -- Aleksander Andreyev 3/26/2019 Reviewed

giz061_Supplemental_Files

## Abbreviations

ARA: arachidonic acid; ANOVA: analysis of variance; BSA: bovine serum albumin; CAT: prefix for category term; CG-MD: coarse-grain molecular dynamics simulation; CHO: Chinese hamster ovary; COPI: coat protein I; CSV: comma separated values; ER: endoplasmic reticulum; GO: gene ontology; HILIC: hydrophilic interaction liquid chromatography; KLA: Kdo_2_-lipid A; LIN: linoleic acid; LC-MS/MS: liquid chromatography−tandem mass spectrometry; LION: lipid ontology; LOOCV: leave-one-out cross-validations; PA: phosphatidic acid; PAL: palmitic acid; PC: phosphatidylcholine; PDA: pyrene decanoic acid; PE: phosphatidylethanolamine; PG: phosphatidylglycerol; PI: phosphatidylinositol; PM: plasma membrane; PS: phosphatidylserine; TLR4: toll-like receptor 4; v/v: volume/volume.

## Competing interests

The authors declare that they have no competing interests.

## Funding

This research was supported by grant No. BB/2012-10047 of the biobased ecologically balanced sustainable industrial chemistry (BE-BASIC) project to J.B.H. and J.F.B.

## Authors’ contributions

M.R.M. and J.B.H. conceived the project. M.R.M. developed LION and LION/web and performed the experiments. A.J. tested and suggested improvements for LION/web. C.H.A.v.d.L. and T.A.W. contributed to the regression models and statistical concepts. C.H.A.v.d.L. and J.F.B. contributed to the lipidomics data processing and analysis. M.R.M. and J.B.H. wrote the manuscript.
