# Supplementary material for: LION/web: a web-based ontology enrichment tool for lipidomic data analysis
Source: Gigascience. 2019 May 29;8(6):giz061. doi: 10.1093/gigascience/giz061 (PMC6541037; doi:10.1093/gigascience/giz061)
Supplement: giz061_GIGA-D-18-00357_Revision_1 [file giz061_giga-d-18-00357_revision_1.pdf]

GigaScience

LION/web: a web-based ontology enrichment tool for lipidomic data analysis

--Manuscript Draft--

**Manuscript Number:**	GIGA-D-18-00357R1
**Full Title:**	LION/web: a web-based ontology enrichment tool for lipidomic data analysis
**Article Type:**	Technical Note
**Funding Information:**	
**Abstract:**	A major challenge for lipidomic analyses is the handling of the large amounts of data and the translation of results to interpret the involvement of lipids in biological systems. We built a new lipid ontology (LION) that associates over 50,000 lipid species to biophysical, chemical and cell biological features. By making use of enrichment algorithms, we used LION to develop a web-based interface (LION/web, www.lipidontology.com) that allows identification of lipid-associated terms in lipidomes. LION/web was validated by analyzing a lipidomic dataset derived from well-characterized sub-cellular fractions of RAW 264.7 macrophages. Comparison of isolated plasma membranes with the microsomal fraction showed a significant enrichment of relevant LION-terms including 'plasma membrane', 'headgroup with negative charge, 'glycerophosphoserines', 'above average bilayer thickness', and 'below average lateral diffusion'. A second validation was performed by analyzing the membrane fluidity of CHO cells incubated with arachidonic acid. An increase in membrane fluidity was observed both experimentally by using pyrene decanoic acid and by using LION/web, showing significant enrichment of terms associated with high membrane fluidity ('above average', 'very high' and 'high lateral diffusion', and 'below average transition temperature'). The results demonstrate the functionality of LION/web, which is freely accessible in a platform-independent way.
**Corresponding Author:**	Bernd Helms<br><br>NETHERLANDS
**Corresponding Author Secondary Information:**	
**Corresponding Author's Institution:**	
**Corresponding Author's Secondary Institution:**	
**First Author:**	Martijn R. Molenaar
**First Author Secondary Information:**	
**Order of Authors:**	Martijn R. Molenaar
	Aike Jeucken
	Tsjerk A. Wassenaar
	Chris H. A. van de Lest
	Jos F. Brouwers
	Bernd Helms
**Order of Authors Secondary Information:**	
**Response to Reviewers:**	For a formatted version with answers to the comments in blue, please see uploaded document 'Response to reviewers'<br><br>Ruth Welti (Reviewer 1)<br><br>LION provides useful information helping users associate lipidomics data on membrane lipid species from mammalian systems with the chemical and physical properties of those systems. Overall this is an ambitious undertaking that is likely to provide insights on lipid properties, particularly to users that are not familiar with

Powered by Editorial Manager® and ProduXion Manager® from Aries Systems Corporation

chemical or physical properties of membrane lipids. Overall, the tool seems useful and the paper is well-written, but a few points could be explained in more detail.

We appreciate the positive and constructive comments of the reviewer.

#1. It should be mentioned, and perhaps the authors could include an explanatory note at the site, noting that actual physical properties of membranes (such as fluidity) depend on factors in addition to the typically measured lipids, including sterols and protein type and content.

We incorporated a statement about this aspect at three different locations: i) in the web-tool (on the '?' sign, beneath the results output); ii) in a new F.A.Q. that is now available via the website; and iii) in the Discussion (line 200-204).

#2. It might be useful to point out specifically that the samples chosen to "calibrate" the lipid categorization are all from mammalian cells and thus the ability to accurately interpret lipidomics data from other types of systems is not clear. Perhaps this is because it is not clear to the reviewer precisely how the categorized lipids (page 4, lines 69-74) were used in the analysis. Since many mammalian tissues (e.g. brain, heart) have more extreme compositions, will this be a problem for analysis?

Indeed, we made use of mammalian lipidomics datasets as reference to define the groups of three biophysical properties. To emphasize this, we included a comment on LION's focus on mammalian lipidomes in the Discussion (line 193-197). This will, however, not compromise the results in specific examples as mentioned by the reviewer as the principle of LION/Web is based on sample comparison (Fig.1, sample A and B). A comparison between tissues with more extreme compositions (e.g. brain and liver) is likely to result in enriched terms related to very low Tm's or very high lateral diffusions, and in different lipid classes/species, results that reflect the respective lipidomes. Comparison between samples from the same tissue (e.g. wt brain vs. geneX-/- brain) will often yield more subtle differences, depending on the knockout. However, LION/Web will report any significant difference, e.g. if geneX affects lipid composition. The statistical power of the significance can be further increased by increasing the number of replicates (n).

#3. The ranking approach appears to be a pairwise comparison. I.e., even when multiple samples are present, comparison is to one (control) sample. This is analogous to a typical transcriptomic approach but, given that it's actually easier to collect lipidomic data than transcriptomic data on hundreds of samples/conditions, having to analyze the data pairwise might be a bit burdensome. Maybe you could discuss the choice of approach in the paper or clarify if the reviewer's understanding is incorrect.

We thank the reviewer for this comment. We have extended the web-tool with more options to calculate local statistics (values that are used to rank lipids). One of these options is the use of p-values derived from one-way ANOVA F-tests. This statistic analysis allows comparison of multiple conditions and can be used to rank the most fluctuating lipid species in datasets. Subsequent enrichment analysis will result in LION-terms summarizing these lipids.
A second option that we included to characterize lipidomic datasets with more than 2 conditions, is the use of hierarchical clustering in combination with the target-list mode. A new figure (Figure 2B) illustrates this approach using the same public dataset that we used in the initial version of the manuscript. Enrichment analysis of the lipids in the clusters, in combination with a visual presentation of the clusters in relation to the conditions, further aids in characterization of the full dataset.

#4. An example showing the output from the target mode would be helpful to the reader.

We agree with the reviewer that the manuscript would benefit from an example of the target mode. As mentioned above (#3) we now include a new figure (Figure 2B) that shows a clustered heat map of the RAW 264.7 macrophages dataset. Each cluster is characterized by assessing LION-term enrichment of the lipids within each cluster, as compared to all the lipids in the experiment.

Aleksander Andreyev (Reviewer 2)

This technical note describes a LIpid-related ONtology database (LION) and accompanying enrichment analysis tool with potentially high value for lipidomics research. According to the authors they aim to "bridge<> the gap between lipidomics and cell biology" (p.7, l.138). A mere attempt at this herculean task is highly commendable. This entails, however, that the narration should be comprehensible for a non-expert user, presumably a cell biologist with little understanding of bioinformatics (which would be also in line with the GigaScience editorial guidelines). Unfortunately, the manuscript is plagued with multiple issues that make it very hard to understand the utility and intended use of the tools and nearly impossible to evaluate their validity. From the way manuscript is written, it feels as if it is intended more for bioinformatics audience which almost defeats the purpose. It is also somewhat disorganized with the logical flow being interrupted by off-hand remarks and description of one topic spread over different parts of the manuscript, sometimes repetitively. In a few cases, the text is burdened with statements of the obvious (e.g., "lipid structure is closely related to lipid function", "allows identification of lipid-associated terms in lipidomes"). There are multiple typos, grammar errors and misused words or terms that make a mere reading of the article a torture. One step to address this issues might be including subsections under the Findings section, another - careful reassessment of what material represents technical side and belongs to Methods and what should be in the Findings (my feeling is that a good portion of the LION description, currently under Methods, actually belongs to the Findings, right after the background information). The same goes to figure legends - I think currently they are overloaded with information that belongs in the Methods. The manuscript suffers from frequent use of vague statements. Instead of describing WHAT was done the authors simply state the means for doing it: "we used" this or that, "we made use of" this or that, such and such "was used", etc. Instead of explaining HOW something was done a bare statement "based on" is often made. References are missing (e.g., "as described in the literature", p.4, l.53, "was reported", p.5, l.99). The tally of connections between membrane biophysics and cell biology (p.3, l.35-43) looks random and lacking completeness. Besides, it seems somewhat misplaced. Authors use what appears to be in-house or jargon terms, such as "by target list", "by ranking" for the modes of the enrichment analysis, "local" statistics, etc. Use of such terms should be avoided. For such important terms as the modes of analysis the names should be related to their function and, ideally, self- explanatory (or, at least, thoroughly explained). All these issues pertaining to the quality of the narration should be addressed before the substance of the work can be properly evaluated.

We thank reviewer #2 for his thorough review report and would like to apologize for the typos and grammar errors in the manuscript that made ‘reading of the article a torture’. As suggested, the Findings section is now subdivided into subsections with headings. We also include a separate Discussion section to avoid the ‘interruption by off-hand remarks’.

Indeed, LION/web is intended to be useful for non-experts in bioinformatics. We recognize that some concepts used in the manuscript might be difficult to grasp with limited bioinformatics experience. Nevertheless, some basic understanding of data-analysis must be expected from users that obtained omics-data (which is obviously a prerequisite to use LION/web). In the updated version, we have provided more explanation and illustrate some of the concepts with examples in the following ways:
i) throughout the manuscript, we added additional information.
ii) we added a point-by-point frequently asked question (F.A.Q.) section in the web-tool, that can be accessed via the main menu of the website.
iii) we added ‘tooltips’ in the LION/Web application. Tooltips are pieces of information or instructions that appear when users hover the mouse cursor over an item - without clicking on it. This allows for specific instructions for specific steps.
Upon the reviewer’s request, we have considered several alternative names for the

Powered by Editorial Manager® and ProduXion Manager® from Aries Systems Corporation

enrichment modes (ranking and target-list mode). However, we found the initial names to be the clearest, as it describes the difference between the modes the best. The use of a target-list (usually referred to as gene list, ID list, etc.) is also common practise in gene ontology enrichment procedures (DAVID, Panther, GOrilla). Users who have experience in this field will recognize the concept ‘target-list’. To improve the understanding of these terms/modes, we included more details about both modes in the Methods section. In addition, we added a new figure (Figure 2A+B) to illustrate the target-list mode.

With respect to the comment “The tally of connections between membrane biophysics and cell biology (p.3, l.35-43) looks random and lacking completeness” we note that the listing of biophysical properties related to membrane biology in the background section was not intended to be complete, but to provide a few intuitive examples. To clarify this, we put these examples in parentheses and ‘e.g.’.

Concerning missing references: Details about references per data source is now available via Supplemental Data 1. The statement ‘was reported’ (page-5/line-99 of initial manuscript) refers to LION-terms that were reported by the web-tool.

However, even in the present state the manuscript allows to point out the following weaknesses/areas for improvement:

#1. The LION should be completely verbally described (beyond the present reference to the .obo file). This should include a list of categorical ontology terms and rules of association between them. For the ones that are not obvious, a justification should be provided. As it stands now, the terms in question are hidden inside 1275-page long Excel file among about 50,000 terms representing individual lipids. Some of them relate to conventional structural elements of lipids, others are less obvious. For example, "fatty acid with 16-18 carbons" - is there any scientific meaning in this term? What is so special about this particular chain length? What exactly are the extra levels of classification between lipid classes and species? - they are mentioned but not described.

Upon the reviewer’s request, we describe LION in a better structured way by inclusion of two additional tables:
(1) Supplemental Data 1; describing all LION-terms (excluding lipid species), with detailed information about hierarchy, classification and references.
(2) Supplemental Data 2; describing all lipid species present in LION.

Concerning the scientific meaning of terms: one of the guiding principles of LION was to be able to construct defined subsets of lipids (‘terms’). LION/web then aids to report the most interesting subsets. Some of these subsets might be of interest, others might not. Scientific meaning should be evaluated by the scientist. For example, "fatty acid with 16-18 carbons" might indeed sound trivial at the first sight. Nevertheless, its enrichment could hint towards testable biological hypotheses.

#2. The enrichment tool is the crux of the article, the thing the authors are trying to "sell". However, there is no description of what it does and how it can be used. I flatter myself to be a qualified used but I could not make a head or tail of what the so called "by target list" mode does. If my "target list" includes unsaturated lipids I'll get enrichment in "double bonds", "below average transition temperature", etc. That much is obvious without running the tool. What else? What are the scenarios when I need to use it? Why do I need two lipidomic data sets for this? What does "derived from thresholding or clustering" mean?

We recognize that in the initial version of the manuscript, the use of the ‘target-list mode’ was not illustrated. We added an extra figure (figure 2) that demonstrates the use of both modes using the RAW 264.7 macrophages dataset (figure 2A+B for the target-list, figure 2C for the ranking mode). Figure 2C was a supplemental figure in the original manuscript.

The second mode, apart from the name (why "by ranking"? isn't this purely technical approach to facilitate stat analysis?), is less problematic. However, the option to limit analysis to a specific set of terms ("terms of interest") should be mentioned upfront.

Powered by Editorial Manager® and ProduXion Manager® from Aries Systems Corporation

Then, the questions arise in what scenarios this would be advantageous? Would this create a bias in the analysis or not, both with regard to outcome and its stat significance?

We now describe the selection of specific sets at an earlier stage.

#3. The claim of the scope is overreaching. The "function" category, most interesting for cell biology researchers, appears to be extremely frugal, limited just to the crudest distinction between structural, signaling and storage functions. If this perception is correct, the LION would be of limited value for cell biology. The "chemical" properties appear to be a misnomer with chemical information limited purely to structural elements with no regard to reactivity, biochemical synthetic pathways, etc. I would say that, according to this Technical Note, the LION is the ontology linking lipidomics data to biophysical properties of corresponding membranes. The testing of the ontology was performed in a set of assays pertaining to membrane biophysics.

We found a single occurrence of 'cell biology' ('...web-tool bridges the gap between lipidomics and cell biology...') in the initial manuscript. This claim is now phrased with greater caution by '... future expansions of the LION database..., LION/web will be increasingly successful to bridge the gap between lipidomics and cell biology.' (line 216-218). However, we believe that besides 'function', also 'cellular component' and the biophysical properties are of interest for scientists studying cell biology. In addition, we will maintain the LION database and update it when new lipid data and functions of individual lipid species or classes become available (see also our reply to the comment of the expert editorial board member).

#4. It would be advantageous to sync terminology with other ontologies whenever possible, for example, use the GO term "cellular component" instead of "cellular localization", etc. "Lipid component" is a very dubious term for a structural lipid.

As suggested, we replaced the LION-term name "cellular localization" by "cellular component". "Lipid component" was a typo in the manuscript, and not the name of a term in LION. We apologise for this mistake.

#5. The biophysical properties of the vast majority of lipids were inferred from a limited set of literature data. It is therefore of utmost importance to thoroughly describe the approach used. What kind of data the sources provided? Where they for individual lipids or mixes, measured or calculated? How many entries? The equations for the multiple linear (sic!) regression analysis should be shown. The resulting coefficients could be of value by itself - why not publish them here?

We thank the reviewer for noticing the missing word 'linear'. We replaced multiple occurrences of 'multiple regression analysis' by 'multiple linear regression analysis'. As mentioned earlier, we now include a supplemental table with data sources per LION-term. The raw numeric values (per lipid) of the biophysical properties derived from these sources were already provided together with the original manuscript in 'scripts' folder. It is our understanding that this folder is available to the reviewers (and to the public after publication).

We appreciate the suggestion to report the coefficients of the models. To this end, we now include an Excel spreadsheet containing the coefficients of the models, together with input cells to predict (numerical) values of the biophysical properties (Suppl. Data 8).

#6. The lipids appear to be divided into "quintiles" using a hard-to-describe (and almost lacking description in the manuscript) procedure based initially on a number of lipids in each group rather than the value of a biophysical parameter. What is the rationale for this? Does transition temperature of a lipid membrane care how many other membranes share the same value? I think the categorization should be based upon the magnitudes of biophysical properties alone. By the way, how many groups are actually there? The text says 5 but Fig. 2 shows 7… Also, Fig. 2 shows FDR q-values which are not mentioned in either legend or the main text.

Powered by Editorial Manager® and ProduXion Manager® from Aries Systems Corporation

We categorized ‘transition temperature’ into 5 groups: very low, low, etc. These descriptions are not defined and intrinsically subjective: whether a membrane has a low Tm depends on the context. To provide this context, we selected four lipidomics studies to serve as reference. Lipids from these reference lipidomes were ranked based in the predicted numeric values of the biophysical property. Then, the first 20% (first quintile) was defined as ‘very low’, the second 20% (second quintile) as ‘low’, etc. The limits of these quintiles were then used to classify all lipids present in LION. We believe that this approach defines the group limits with more physiological relevance. The alternative approach, based on magnitudes of biophysical properties alone (as suggested by the reviewer) is more likely to yield a quintile ‘average’ for a group of non-physiological lipids.

The confusing of 5 groups vs. the 7 groups in figure 2 (now figure 3) is related to hierarchy. The groups ‘very low ...’ and ‘low ...’ are linked to a parental group called ‘below average ...’. The same goes for ‘high ...’ and ‘very high ...’, they are linked to ‘above average ...’. We updated the figure by adding a graphical representation of this hierarchy to the figure (new figure 3D). The hierarchy of LION-terms is also depicted in supplemental Data S1.

We now include a reference to ‘q-values’ in the figure legends.

#7. It is not absolutely clear from the manuscript but appears that the enrichment tool relies on the significance of the changes (p-value), as opposed to magnitude, to evaluate enrichment. Is this true? Is it possible that highly significant changes in low abundance lipids would dominate the outcome list without having much effect on the properties of membrane?

All enrichment analyses in the initial version of the manuscript used the ranking-mode with one-tailed t-test p-values to rank the lipids. Other statistical methods could be considered, but every choice has its pros and its cons. Magnitude (fold-change of condition B over condition A) has the undesirable property to overestimate effects when lipid concentrations are close to noise levels: it does not take sample variance into account. In contrast, p-values are more robust, but might be less intuitive to users without strong background in statistics. Using p-values, it is potentially possible that ‘highly significant changes in low abundance lipids could dominate the outcome list’. However, most low abundant lipids usually display higher variance due to lower signal/noise levels. As a result, they usually do not generate extreme low p-values.

To provide more flexibility for users and to make the choice of a local statistic explicit, we now offer three local statistics (one-tailed t-test p-values, 2log fold-change, F-test p-values) in the updated version of the web-tool. The statistical method must be selected each time an analysis in the ranking mode is initiated.

#8. More detail should be provided on the statistics, for example, how the distribution curve was generated for K-S analysis, what were the input parameters for the Fisher exact test, etc.

We added more information in the Methods section.

#9. Methods for PDA assay and LC-MS should be brought to compliance with editorial guidelines to allow duplicate these studies. Missing are parameters such as cell number, concentration of the dye, shape of LC gradient, LC system used, MS/MS settings, to name a few. The full name of the Fusion mass spec should be provided because there are several different models. The text is not clear on the sequence of events: it sounds as if analyte ions fly from orbitrap to linear ion trap for detection - is this even possible?

We added details about the PDA in the manuscript.

The methods for LC-MS have now been described in greater detail to facilitate easy replication of experiments. Parallelization of MS1 and MS2 experiments has been clarified to avoid confusion. Current versions of the MS instrument are branded as ‘Fusion Lumos’ or ‘Fusion IDX’. However, the original ‘Orbitrap Fusion’ mass spectrometer (serial number FSN10438) was branded under that name and this is the

model used in our studies. Therefore, we cannot specify the type of instrument more accurately than we currently do.

#10. With regard to membrane fluidity data, although they show the desired differences they could be made much more convincing with appropriate controls subtracting intrinsic fluorescence of the cells.

The membrane fluidity data presented in the manuscript were subtracted from background fluorescence (blanks were samples with cells but without PDA dye). To make this clear, we updated the Methods section with this information.

#11. Annotating lipids with the "most abundant fatty acid composition" is misleading - if isobaric species are not resolved the overall composition (total carbons, total double bonds) should be shown as primary annotation (possibly followed by the most abundant isomer).

We now include the overall composition as primary annotation, together with a second column containing the most abundant isomer (Data S4). MS/MS analysis allows identification of the most abundant isomer (e.g. PC with a C18:1 and a C18:0 fatty acid) without assignment of the sn1/sn2 position of the respective fatty acids. It is important for experiments such as described in figure 3A to use identifiers containing individual fatty acids. LION-terms related to fatty acids cannot be associated to a dataset that lacks this information. To avoid confusion, we have renamed the lipid species from e.g. PC(18:1/18:0) to PC(18:1_18:0) to indicate the fatty acid composition of lipid species without sn1/sn2 assignment.

Expert editorial board comments on usability:

The following comments are thus from the perspective of a potential user.

Can the authors specify the source of the 50,000 lipid species included into the analyses? To my knowledge the lipidmaps database reports around 42,000 entries only.

We used the lipid classification system (hierarchy) in accordance with LIPIDMAPS. The individual lipid species in LION were constructed by combining lipid classes with abundant fatty acids. LIPIDMAPS is probably somewhat more stringent about the inclusion of lipid species in their database as they intend to provide additional information for individual species. We added a few lines (231-236)
about the construction of individual lipid species in LION to the Methods section.

The number of lipid species linked to experimental or in silico data is more than two orders of magnitudes lower that the indicated number of 50,000 and mainly refers to membrane lipids. Are all of these 50,000 species associated with one or more than one feature? Can the authors comment how many of these 50,000 lipids are associated with features going beyond chemical properties? What kind of cell biological features were used and which of these features where linked to which lipid species? In order to understand and validate the assignments as more detailed description would be helpful.

Many lipids have a number of associations, whereas some lipids only have a few. As a consequence of the hierarchical structure of LION, lipids with only one association will not occur: lipids are (indirectly) associated with the neighbour’s neighbour. To make this information more accessible for users, we improved the enrichment-report , which can be obtained by the button ‘download report’. It now contains three files: a CSV-file with the enrichment information, a CSV-file containing all the LION-terms associated with the lipids in the dataset, and vice versa, a CSV-file containing all lipids of the dataset with associated LION-terms. With this information, users are better equipped to understand the underlying data structures and improve interpretation of obtained results.

Can the authors comment on why they integrated coarse-grain but not (in addition) atomistic MD data?

	To our knowledge, there is no comprehensive lipid dataset available that has been obtained by atomistic molecular dynamics simulations. More importantly, the biophysical properties are categorized into distinct groups (very low, low, average, etc.). Given this categorization in groups, we suspect that the increased resolution of atomistic MD will be of no or very limited added value.<br><br>Can the authors specify which data of the two papers in particular was included into building the application?<br><br>We now provide a detailed supplemental table (Data S1) containing references per LION-term. Moreover, the source data and code are available via the script folder.<br><br>The fact that the application in its current form is restricted to glycerol-based lipids and fatty acids should be indicated in the abstract and in the discussion of the dataset.<br><br>We agree that the current LION database is not a complete end product. However, it is not true that LION only contains (associations to) glycerol-based lipids and fatty acids. The database includes many more: sphingolipids (sphingomyelins, ceramides, glycosphingolipids), cholesterol derivatives and retinoids. As comprehensive biophysical data about these classes is hardly available or too complex in the case of cholesterol, not all these classes are associated with biophysical properties. The biophysical properties obtained by MD are limited to glycerol-based lipids. The transition temperatures are also associated with sphingomyelins. Cellular component, intrinsic curvature, headgroup charge are associated with many lipid classes. Limitations of LION/web are included in the Discussion section.<br><br>For this first version of LION the authors included only information from two publications. There is an increasing amount of data available going beyond this information. Can the authors comment on how they plan to allow for integration of additional information? Will users be able to do so in a ‘customized’ fashion?<br><br>We recognize the importance to involve users in the improvement of LION and LION/web. To this end, we added several features to the web-tool.<br>(i) We include an option (not selected by default for privacy reasons) that -when selected- informs us when lipids could not be matched to LION. This helps us to keep track of lipid identifiers that are often used, but not present in LION.<br>(ii) We include a contact form on the website to lower the threshold to contact us for questions, requests, suggestions or feedback.<br>Web-tool improvement will not stop after publication. Currently, we are working on features to build heat maps and principle component analyses within the web-tool. When new sources containing useful data become available, this will be added this to the database.<br><br>The power of application depends on the number of features associated with each lipid species. Can the authors comment on how they plan to advance the data base, e.g., by including the community? Will the application be hosted and if so, what is the perspective?<br><br>The full ontology, R-packages to perform enrichment analysis and R-code for the web-tool is publicly available. This is sufficient for experienced users to build customized versions of LION or the web-tool. We understand, however, that this will be challenging for inexperienced users. In the future, we plan to build a dedicated LION R-package with detailed instructions and guidelines to augment the ontology by individual users. An R-package provides more flexibility than a web-tool and the use of user-customized ontology versions will be easier to support.<br><br>The web-tool is currently hosted by Shinyapps.io. It will be hosted elsewhere in case this service discontinuous its operation. The domain name lipidontology.com is owned by the department and the web-tool LION/web will remain accessible via lipidontology.com.
**Additional Information:**	
**Question**	**Response**

Are you submitting this manuscript to a special series or article collection?	No
**Experimental design and statistics**<br><br>Full details of the experimental design and statistical methods used should be given in the Methods section, as detailed in our Minimum Standards Reporting Checklist. Information essential to interpreting the data presented should be made available in the figure legends.<br><br>Have you included all the information requested in your manuscript?	Yes
**Resources**<br><br>A description of all resources used, including antibodies, cell lines, animals and software tools, with enough information to allow them to be uniquely identified, should be included in the Methods section. Authors are strongly encouraged to cite Research Resource Identifiers (RRIDs) for antibodies, model organisms and tools, where possible.<br><br>Have you included the information requested as detailed in our Minimum Standards Reporting Checklist?	Yes
**Availability of data and materials**<br><br>All datasets and code on which the conclusions of the paper rely must be either included in your submission or deposited in publicly available repositories (where available and ethically appropriate), referencing such data using a unique identifier in the references and in the "Availability of Data and Materials" section of your manuscript.<br><br>Have you have met the above requirement as detailed in our Minimum	Yes

Standards Reporting Checklist?	

Revised Manuscript text

Click here to view linked References

Click here to access/download;Manuscript;20190227 LION Manuscript_after review final.docx

LION/web: a web-based ontology enrichment tool for lipidomic data analysis

Martijn R. Molenaar[§], Aike Jeucken[§], Tsjerk A. Wassenaar[‡], Chris H. A. van de Lest[§], Jos F. Brouwers[§], J. Bernd Helms*[§]

[§] Department of Biochemistry and Cell Biology, Faculty of Veterinary Medicine, Utrecht University, 3584 CM, Utrecht, The Netherlands

[‡] Groningen Biomolecular Sciences and Biotechnology Institute and Zernike Institute for Advanced Materials, University of Groningen, Nijenborgh 7, 9747 AG Groningen, The Netherlands

* To whom correspondence should be addressed: J.B.H (J.B.Helms@uu.nl)

ABSTRACT

A major challenge for lipidomic analyses is the handling of the large amounts of data and the translation of results to interpret the involvement of lipids in biological systems. We built a new lipid ontology (LION) that associates over 50,000 lipid species to biophysical, chemical and cell biological features. By making use of enrichment algorithms, we used LION to develop a web-based interface (LION/web, www.lipidontology.com) that allows identification of lipid-associated terms in lipidomes. LION/web was validated by analyzing a lipidomic dataset derived from well-characterized sub-cellular fractions of RAW 264.7 macrophages. Comparison of isolated plasma membranes with the microsomal fraction showed a significant enrichment of relevant LION-terms including 'plasma membrane', 'headgroup with negative charge, 'glycerophosphoserines', 'above average bilayer thickness', and 'below average lateral diffusion'. A second validation was performed by analyzing the membrane fluidity of CHO cells incubated with arachidonic acid. An increase in membrane fluidity was observed both experimentally by using pyrene decanoic acid and by using LION/web, showing significant enrichment of terms associated with high membrane fluidity ('above average', 'very high' and 'high lateral diffusion', and 'below average transition temperature'). The results demonstrate the functionality of LION/web, which is freely accessible in a platform-independent way.

KEYWORDS

lipidomics; lipids; membrane biology; lipid ontology; LION; LION-term enrichment analysis; membrane biology; web-tool; data analysis; LION/web

BACKGROUND

The comprehensive study of lipids, also termed lipidomics, is gaining momentum. Instrumentation is becoming increasingly more sensitive, precise and fast, and the use of lipidomics to address key questions in membrane biology has become widespread. As a result, datasets are rapidly increasing both in terms of size and complexity. Due to a lack of methods to perform global and in-depth data mining, lipidomic research tends to focus on individual lipid classes or lipid species. A common approach in other ‘omics’ disciplines to reduce complexity is the use of ontologies *e.g.*, Gene Ontology [1], Chemical Entities of Biological Interest ontology [2], combined with statistical tools to determine terms of interest. Although lipid structure is closely related to lipid function, it is currently impossible to associate properties of individual lipids with complex lipid mixtures of cellular lipidomes. Examples of biophysical properties that play an important role in membrane biology are numerous and include membrane thickness (*e.g.*, as driving force in the sub-cellular localization of proteins [3]), membrane fluidity (*e.g.*, regulating bacterial survival [4], membrane heterogeneity in cellular signaling [5]), intrinsic curvature (*e.g.*, of lipids as key player in lipid droplet biogenesis [6,7] or COPI coat disassembly [8]), and net charge (*e.g.*, of membranes as a determinant in lipid-protein interactions [9]). Here, we aim to provide a lipid ontology database and complementary enrichment analysis tool that (i) contains chemical and biophysical information of lipid species, (ii) is platform independent and compatible with routine mass spectrometry-based lipid analysis, (iii) can be used by researchers without computer programming skills, and (iv) is freely available to the scientific community.

FINDINGS

Basic structure of LION

We constructed an ontology database called LION (**File S1**) that links over 50,000 lipid species with four major branches: 'lipid classification' (the LIPIDMAPS classification hierarchy [10]), 'chemical and physical properties' (fatty acid length and unsaturation, headgroup charge, intrinsic curvature, membrane fluidity, bilayer thickness), 'function', and 'subcellular component' (predominant subcellular localisation). The resulting database contains more than 250,000 connections ('edges'), providing a detailed system for in-depth annotation of lipids. An example of all LION-terms associated with a single phosphatidylserine (PS) lipid species, PS(34:2), is depicted in **Figure S1**. We describe the construction of LION in more detail in the Methods section. All LION-terms, classification rules and references are described in **Data S1**, all lipids currently supported by LION are described in **Data S2**.

Addition of biophysical properties to LION

An important feature of LION is the association of lipid species with biophysical properties. We made use of experimental data (from five phospholipid classes and sphingomyelin) [11] and data (from five phospholipid classes) obtained by coarse-grain molecular dynamics simulation (CG-MD) [12], each providing distinct biophysical properties . These data were used to estimate the biophysical properties of all related lipids in the LION-database by multiple linear regression analysis.

The regression models were validated in two ways. First, we performed leave-one-out cross-validations (LOOCV) of all three models (**Fig. S2 A-C)**, showing satisfactory agreement between determined and predicted values. Second, we compared two properties closely associated with membrane fluidity: 'transition temperature' (from experimental datasets) and 'lateral diffusion' (from the CG-MD datasets) **(Fig. S2 D)**. As expected, lipids with low

transition temperatures were predicted to have high lateral diffusion values at a defined simulation temperature and vice versa.

Subsequently, all numerical datapoints for each biophysical property were categorized into five pre-defined groups (‘very low’, ‘low’, ‘average’, ‘high’, ‘very high’). We aimed to find group definitions with physiological relevance. Therefore, limits of each group were calculated on the basis of four mammalian lipidomics publications that served as a reference [13-16]. Using these group definitions, numerical values of all applicable lipid species present in LION were classified and connected to their respective LION-term (**Fig. S2 E**).

LION enrichment analysis and web-tool LION/web

Next, we used LION as a basis to build an ontology enrichment tool that facilitates reduction of lipidome complexities in an unbiased manner. To this end, we made use of an adapted version of ‘topGO’, an R-package designed for enrichment analysis of GO-terms [17]. Subsequently, we designed a web-tool with R-package Shiny (‘LION/web’, www.lipidontology.com) that offers an intuitive user-interface and supports two major workflows **(Fig. 1)**: enrichment analysis of a subset of lipids of interest (‘target-list mode’) and enrichment analysis performed on a complete and ranked list of lipids (‘ranking mode’, referred to as ‘SAFE’ and described in the context of genes [18]). A detailed step-by-step description of LION/web’s workflow can be found in **Note S1**.

Analogous to Gene Ontology enrichment approaches [1], which facilitate pre-selection of ontology sub-domains or subsets of GO-terms (‘GO-slims’), LION/web offers the option to limit analysis to specific LION-terms of interest. Furthermore, the web-tool allows removal of the most generic LION-term (the one with the highest hierarchy) if a related term contains the same subset of lipids. For example, the term ‘diacylglycerophosphocholines’ might be associated with the same lipids as ‘glycerophosphocholines’. With this option switched on, only the most specific term (‘diacylglycerophosphocholines’) is included in the results.

Performance of 'target-list mode' by LION/web

To test the functionality of LION/web, we made use of a previously published and well characterized dataset containing lipidomics data from several sub-cellular fractions of RAW 264.7 macrophages, with or without TLR-4 activation by Kdo_2-lipid A (KLA) [13] (see Methods for a direct link to the dataset). First, we re-normalized the dataset by expressing all lipid species as fraction of the total amount of lipid per sample. Subsequently, the data were visualized by constructing a heat map graph **(Fig. 2 A)**. Lipid species were grouped into 10 clusters by hierarchical clustering. Each lipid cluster was subsequently analyzed by LION/web, which was able to reformat and match the vast majority (>97%) of the submitted lipids in the dataset. In the 'target-list mode', LION/web assesses the enrichment of LION-terms in a subset of lipids, as compared to all lipids in the experiment. For every cluster, lipids **(Data S3)** were entered as target-list and compared with the background list. Enrichment analysis of all 10 clusters resulted in at least one significant LION-term **(Fig. 2 B)**. The heat map showed that lipids present in clusters 7 and 8 were abundant in the mitochondrial fractions **(Fig. 2 A).** In line with this observation, enrichment analyses of these clusters resulted in significant terms associated with this organelle (*e.g.*, 'diacylglycerophosphoetahnolamines', 'mitochondrion', 'diacylglycerophosphoglycerols', 'headgroup with negative charge'). Similar results were obtained for cluster 6 (terms related to the plasma membrane), and to lesser extent for cluster 9 (terms related to endoplasmic reticulum). Lipids in cluster 5 were more abundant in KLA-treated fractions and resulted in terms reported by LION/web that were associated with low membrane fluidity.

Performance of 'ranking mode' by LION/web

An alternative method to assess enrichment of LION-terms in LION/web is the 'ranking-mode'. In the 'ranking-mode', all individual lipid species of two conditions are compared and ranked based on a 'local' statistic. This local statistic is any numeric value that associates

individual (hence 'local') lipids with the provided conditions. LION/web supports three different local statistics: one-tailed Welch two sample t-tests *P*-values (comparison of 2 conditions); ^{2}log fold-change values (comparison of 2 conditions) and one-way ANOVA F-tests *P*-values (comparison of >2 conditions). Subsequently, the distributions of all associated LION-terms over the ranked list are compared to uniform distributions by using one-tailed Kolmogorov–Smirnov tests ('global' statistics, as full lipidomes are assessed). A LION-term is enriched when its associated lipids are higher ranked than expected by chance. To illustrate the 'ranking mode', we compared the isolated PM fraction (samples #19-21 from **Fig. 2 A)** with the ER fraction (samples #13-15 from **Fig. 2 A)** from non-stimulated macrophages using one-tailed Welch two sample t-tests *P*-values as local statistic. Subsequently, LION/web assessed all LION-terms for enrichment **(Fig. 2 C)**. In good agreement with current descriptions of the selected organelles [19,20], significant enriched LION-terms included terms associated with chemical descriptions (*e.g.*, 'glycerophosphoserines', 'headgroup with negative charge', 'phosphosphingolipids'), biological features ('plasma membrane') and biophysical properties (*e.g.*, 'above average bilayer thickness', 'below average lateral diffusion', 'very low lateral diffusion', 'very high bilayer thickness', 'neutral intrinsic curvature'). LION/web also reported the significant enrichment of 'very high transition temperature', which is in line with the (very) low lateral diffusion terms (see also **Fig. S2 D**). The term 'very low transition temperature' was also reported to be significantly enriched. Inspection of the lipid species responsible for the LION-term 'very low transition temperature' revealed the presence of lipids that all contain polyunsaturated fatty acids (PUFAs) with at least four unsaturations. This may be a macrophage-specific phenomenon, related to their involvement in inflammation [21].

Enrichment performance of chemical and biophysical LION-terms

To further characterize the enrichment of chemical and biophysical properties by LION/web, we used two different experimental approaches. First, we investigated the enrichment of

chemical features that can be easily incorporated into lipids. To this end, CHO-k1 cells were incubated overnight in the presence of palmitic acid (PA), linoleic acid (LA) or arachidonic acid (AA) complexed to bovine serum albumin (BSA). Subsequently, lipids were analysed by LC-MS/MS and quantified. When available, we used MS/MS data to annotate lipids with their most abundant fatty acid composition. This level of annotation is important as it enables LION to link input lipids with terms associated with fatty acids **(Data S4** and **Fig. S3).** Next, the web-tool was set to use the 'ranking mode' and to limit analysis to LION-terms indicating the presence of fatty acids as lipid building blocks. LION/web reliably reported the significant enrichment of the respective fatty acid in the three different conditions **(Fig. 3 A** and **Data S5)**.

Second, to investigate the enrichment of biophysical LION-terms, we incubated CHO-k1 cells with arachidonic acid (AA). This procedure is known to increase membrane fluidity [22]. After incubation, the membrane fluidity properties of the samples were analyzed both experimentally and by LION/web. Membrane fluidity was experimentally assessed using pyrene decanoic acid (PDA) **(Fig. 3 B)**. This fluorescent probe can exist as monomer or excimer, resulting in a shift of its emission spectrum. The ratio of excimer over monomer fluorescence is proportional to the degree of membrane fluidity [23]. As expected, the ratio of excimer/monomer forms of PDA revealed a significant increase in membrane fluidity of lysates in the presence of AA **(Fig. 3 C)**. For parallel LION/web analysis of membrane fluidity properties, lipids were extracted from the same samples and analysed by LC-MS/MS **(Data S6** and **Fig. S4)**. LION contains two sets of terms associated with membrane fluidity: 'transition temperature' and 'lateral diffusion'. Accordingly, LION/web was set to limit enrichment analyses to these sets, after which the lipidomic data were analyzed ('ranking mode'). In line with the experimentally measured increase in membrane fluidity, terms associated with high membrane fluidity ('above average', 'very high' and 'high lateral diffusion', and 'below average transition temperature') were significantly enriched in cells that had been treated with AA **(Fig. 3 D** and **Data S7)**.

DISCUSSION

Despite the quick grow of lipidomics and the rise of many tools to process raw data into lipid compositions [24], no automated pipeline to reduce complexity in lipidomic datasets using prior knowledge was yet available. Such a tool facilitates the generation of hypotheses, which is an important aim in many omics experiments. Here, we have presented a new ontology called LION and have used this ontology to build a web-based online LION-term enrichment tool suited to fulfill this need. In a single analysis, trends in complex lipidomic datasets can now be assessed in a standardized way. The web-tool assures that the pipeline is accessible to users that are not familiar with programming languages.

Just like enrichment analysis approaches in other omics fields, LION-term enrichment analysis comes with specific strengths and limitations. The quality and coverage of the underlying ontology is of great importance. For LION, we aimed to support most commonly found lipid species in mammalian systems. In our examples, >85% of the input lipids could be matched to the ontology. Due to the great diversity of lipidomes in different organisms, this coverage could be be lower in user-provided datasets from non-mammalian systems. We hope to support LION's coverage of plant, bacterial and yeast lipidomes better in the future. LION/web offers users several feedback options to keep track of missing annotations and to act specifically upon users' needs.

It is important to note that the enrichment of biophysical properties such as membrane fluidity, membrane thickness and curvature cannot replace functional assays. More factors than lipids alone – protein composition, temperature - are playing important roles. Moreover, the effect of cholesterol is complex and depends on the interaction with other lipids. Therefore, the biophysical effects of cholesterol are not included. Also, the relative amounts of lipids in the described enrichment analysis methods are not taken into account: low abundant lipids contribute equally to enrichment as their high abundant counterparts.

This limitation can be circumvented by defining local statistics that takes abundancies into

account. This type of statistic will become more urgent when lipidomic analyses shifts from mostly semi-quantitative to quantitative analyses in the future.

In summary, LION/web reveals changes in lipid patterns that allow researchers to study the complexity of lipidomes in a biological context. With future expansions of the LION database and of LION/web (also upon request of the scientific community), LION/web will be increasingly successful to bridge the gap between lipidomics and cell biology.

METHODS

Creation of lipid ontology (LION)

We built an ontology database that connects lipid species to the following four major branches: 'lipid classification', 'function', 'cellular component' and 'physical or chemical properties'. For readability, a term is included at the top of each branch to indicates the nature of a LION-branch. These 'category' terms are distinguished from other LION-terms with an ID containing the prefix 'CAT'.

The classification system is based on the LIPIDMAPS classification [10]. LIPIDMAPS does not support lipid species with summed fatty acid. However, this extra layer is useful as it enables mapping or when exact fatty acid compositions of measured lipids are not known. This concept is also used in the Swiss Lipids system [25]. Downstream, individual lipid species belonging to classes described in **Data S1** were constructed as combinations of the following fatty acids: C12:0; C14:0; C14:1; C16:0; C16:1; C18:0; C18:1; C18:2; C18:3; C20:0; C20:1; C20:2; C20:3; C20:4; C20:5; C22:0; C22:1; C22:2; C22:3; C22:4; C22:5; C22:6; C24:0; C24:1; C24:2; C24:3; C24:4; C24:5; C24:6; C26:0; C26:1; C26:2; C26:3; C26:4; C26:5; C26:6 and C26:7. For sphingolipids, sphingosine (d18:1) and sphinganine (d18:0) were used as possible backbones. In the current version, LION does not distinguish between *sn*-positions. Fatty acids were ordered by chain length (low to high) and number of unsaturations (low to high). Altogether, LION contains circa 50,000 lipid species.

The branch ‘function’ comprises three subcategories: ‘membrane component’ (associated with lipids that are primary regarded as structural component of lipid bilayers), ‘lipid-mediated signaling’ (lipids that have been implicated in signaling) and ‘lipid-storage’ (lipids that are associated with storage, primarily in lipid droplets). In the category ‘cellular component’, lipid classes that are enriched in particular cellular organelles are linked to their corresponding organelle terms [7,19,20]. The branch ‘physical or chemical properties’ comprises a number of subcategories. First, a number of chemical descriptions (‘contains fatty acid’, ‘fatty acid unsaturation’, ‘fatty acid length’ and ‘type by bond’) was inferred from the species names. Second, data about ‘intrinsic curvature’ [7,26] were categorized into either negative, neutral or positive curvature. As data on species-level are limited, curvature was assumed to be predominantly headgroup-dependent and fatty acid composition was neglected. The third subcategory, ‘charge headgroup’, was divided into three groups based on structural data: ‘negative’, ’positive/zwitter-ion’ and ‘neutral’ [25]. This last term comprises also lipids lacking a headgroup. The fourth subcategory in ‘physical or chemical properties’ is 'chain-melting transition temperature'. This property is derived from a number of sources, comprehensively reviewed by Marsh [11]. This dataset covers a range of lipid classes in both glycerophospholipids (PC, PE, PG, PA, PS) and sphingolipids (SM). We made use of multiple linear regression analysis with lipid class, fatty acid length and unsaturation as predictors to facilitate data extrapolation to previously unreported lipid species. The obtained model (coefficients are available via **Data S8)** was validated by leave-one-out cross-validation (LOOCV). Briefly, one datapoint from the dataset was taken out, after which the model was rebuilt with the remaining points as training set. Subsequently, the selected datapoint was used as validation sample. This procedure was repeated for all the datapoints (**Fig. S2 C)**. Ontologies contain categorical data and are not compatible with numeric values. Therefore, we classified chain-melting transition temperature values into five distinct categorical data groups: 'very low', 'low', 'average', 'high' or 'very high' chain-melting transition temperature. To define the limits of these intrinsic subjective groups, we used four previously reported

datasets to serve as reference lipidomes [13–16]. From all reported lipids, the transition temperature was predicted by the model. The obtained transition temperature distribution was used to define the groups: the lowest 20% (first quintile) was classified as 'very low', the second 20% (second quintile) as 'low', etc. Subsequently, these limits were used to categorize all lipid species present in LION. Lipids with transition temperature values lower than the lowest limit were defined as 'very low', whereas values higher than the highest limit were defined as 'very high'. A flow-chart of this procedure is depicted in **Fig. S2 E**.

In addition to these experimental data sets, we also used data [12] that was obtained by coarse grain molecular dynamics simulation (MARTINI force-field [27]) and which includes membrane properties 'bilayer thickness' and 'lateral diffusion'. The dataset contains lipids from five common classes of glycerophospholipids (PC, PS, PG, PA, PE), but lacks sphingolipids and sterols. By definition, coarse-grained lipids represent a range of structures. To be able to use the dataset in the ontology system, names of coarse-grained lipids were translated into their representing counterparts. Subsequently, lipid properties were extrapolated to the entire database by multiple linear regression analysis models (with lipid class, fatty acid length and unsaturation as predictors, coefficients are available via **Data S8**) and validated by LOOCV (**Fig. S2 A-B)**. We followed the same procedure as used for transition temperatures; extrapolated results for both properties were categorized into representative classes: 'very low', 'low', 'average', 'high' or 'very high', based on values, predicted by our models, of the reference datasets [13–16].

The initial structure of LION was built with OBOEdit v.2.3.1 [28] and formatted as OBO-file. Subsequently, custom R-scripts connected specific terms with more general terms based on the described datasets. The entire ontology can be found as **File S1.**

Implementation of enrichment analysis tool

To use LION with existing ontology enrichment tools, we used an adapted and generalized version of Bioconductor R-package 'topGO' [17]. This version, called 'topOnto', allows users to include ontologies other than those provided with the package. TopOnto's attached Perl-

script was used to convert the ontology file from OBO- to SQLite-format. Apart from this extra feature, the 'topOnto' package provides the same functionality as the original version. To perform the enrichment analysis, two statistical approaches are used. In the 'target-list mode', one-tailed Fisher-exact statistics are used to test enrichment. To this end, 2x2 contingency tables are constructed for every LION-term, containing the number of lipids associated and not associated with the given term for both the target-list and the background set, and analyzed. In the 'ranking mode', one-tailed Kolmogorov-Smirnov tests are used as 'global' statistics to assess enrichment of LION-terms over a ranked (by 'local' statistics) list of lipids. For every LION-term, the cumulative distribution of associated lipids over the ranked list is compared with the uniform distribution. Enrichment is defined as over-representation of highly ranked lipids associated with the term. To rank input lipids, LION/web offers three different 'local' statistics: *P* values from one-tailed Welch t-tests (2 condition comparison), ^{2}log fold-change values (2 condition comparison) and *P* values from one-way ANOVA F-tests (>2 conditions comparison). Ranking direction (from high to low, or vice versa) is automatically updated after local statistic selection, but can be set manually. In addition, users can use custom local statistics. In both modes, topGO's classic algorithm is selected [17]. After LION enrichment analysis, raw *P* values are corrected for multiple testing (Benjamini-Hochberg). The R-scripts were used to build the user-friendly web-based tool LION/web **(Note S1)** with R-package 'shiny'. The application has been made available on the shinyapps.io server as a free online tool, accessible through http://www.lipidontology.com/.

Cell culture and preparation of fatty acid-albumin complexes

CHO-k1 cells were cultured in Ham's F-12 medium (Thermo Fisher Scientific, Waltham, MA, USU) supplemented with 7.5% FBS (Thermo Fisher Scientific, Waltham, MA, USU), 100 units/ml penicillin and 100 µg/ml streptomycin (Thermo Fisher Scientific, Waltham, MA, USU). Cells were grown in a humidified incubator at 37°C containing 5% CO_2 and passaged

twice a week. Stocks of 10 mM arachidonic acid, linoleic acid, oleic acid, or palmitic acid (all obtained from Sigma, St. Louis, MO, USA) were complexed to 2 mM fatty-acid free BSA (Sigma, St. Louis, MO, USA), filter-sterilized and stored at −20 °C. Control incubations without fatty acid contained equivalent amounts of fatty-acid free BSA. All experimental incubations were performed in plastic 6-well culture dishes (Corning, Tewksbury, MA, USA).

Measuring membrane fluidity

After overnight incubation in the absence or presence of fatty-acids (using fatty acid-free BSA or fatty acids coupled to BSA, respectively), cells were washed and scraped in PBS. Cells were subsequently homogenized on ice with 26-gauge needles (BD Bioscience, San Jose, CA, USA). Homogenates (equivalent to 40,000 cells) were mixed 1:1 with the manufacturer's supplied dilution buffer (Membrane fluidity kit, Abcam, Cambridge, UK) in the absence (background) or presence of 5 µM pyrenedecanoic acid (PDA) and transferred into a 96-well plate (black plastic with glass bottom, Greiner Bio-One, Frickenhausen, Germany). After 30 minutes of incubation at 37°C, fluorescence spectra (excitation at 360 nm, emission between 375-500 nm, 37°C) were measured with a temperature-controlled fluorescence microplate reader (CLARIOstar, BMG Labtech, Offenburg, Germany). Data were processed in R by expressing monomer (370-390 nm) and excimer (470-490 nm) as ratios of mean fluorescence after subtraction of background fluorescence (samples with cells but without PDA). Results were expressed as means. Differences were analyzed by two-tailed Welch's t-tests.

Lipidomics by LC-MS/MS

After incubation, lipids were extracted as described before [29]. Lipid extracts were dried under nitrogen and dissolved in 100 µL chloroform/methanol (1:1) and injected (10 µL) on a hydrophilic interaction liquid chromatography (HILIC) column (2.6 µm HILIC 100 Å, 50 x 4.6 mm, Phenomenex, Torrance, CA). Lipid classes were separated by gradient elution on an Infinity II 1290 UPLC (Agilent, Santa Clara, CA, USA). At a constant flow rate of 1 ml/min,

ACN/acetone (9:1, v/v) was used as solvent A. Solvent B consisted of a mixture of ACN/H2O (7:3, v/v) with 10mM ammonium formate. Both solvents contained 0.1% formic acid. The gradient was as follows (time in min, %B): (0, 0), (1, 50), (3, 50), (3.1, 100), (4, 100). Samples were injected without re-equilibration of the column. The column effluent was connected to a heated electrospray ionization (hESI) source of an Orbitrap Fusion mass spectrometer (Thermo Scientific, Waltham, MA) operated at -3600V in the negative ionization mode. Temperatures for the vaporizer and ion transfer tube were 275°C and 380°C, respectively. Full scan MS1 measurements in the mass range from 450 to 1150 amu were collected in the orbitrap at a resolution of 120.000. Parallelized data-dependent MS2 experiments were done with HCD fragmentation set at 30V, using the dual stage linear ion trap to generate up to 30 spectra per second.

Lipidomics data analysis

Acquired raw datafiles were converted to mzXML-files by msConvert (part of ProteoWizard v3.0.913) [30] and processed with R-package 'xcms' v2.99.3 [31]. After deisotoping, annotation of lipids was performed by matching measured MS-1 m/z values with theoretical m/z values. Lipids with the same or similar m/z values - *e.g.*, BMP(38:4) and PG(38:4) - could by distinguished by differences in retention time **(Fig. S3 and S4)**. Lipid annotation containing individual fatty acids (extra column 'most abundant isomer annotation' in **Data S4**) as used in **Fig. 2 A** and **Fig. S3** was accomplished by examining MS-2 spectra. When MS-2 spectra were available for a given MS-1 peak, the most abundant fatty acid combination was used to annotate the lipid. The resulting experimental datasets, as well as the public RAW 264.7 macrophage dataset [13], were normalized by expressing all lipids as ratios of the sum of all intensities per sample. MetaboAnalyst 3.0 [32] was used to replace missing values (of the RAW 264.7 dataset) by half of the minimum positive value in the original data, and to perform Principal Component Analysis (with Pareto scaling).

Heat map, hierarchical cluster analysis and LION-enrichment analyses

The heat map of the RAW 264.7 dataset was constructed after calculating z-scores for all lipids (all lipids were scaled to a mean of zero and a standard deviation of 1) using R-package 'pheatmap v1.0.10'. Lipids were grouped by hierarchical clustering. The dendrogram of the lipids on the y-axis of the heatmap used Euclidean distance as the similarity measure and was performed with complete linkage. The number of clusters was set to 10. Enrichment analysis of each of the 10 clusters was performed using the 'target-list mode' with default settings. Enrichment analyses used in **Fig. 2 C** and **Fig. 3 A and D** were performed using the 'ranking mode', with one-tailed Welch two sample t-tests P-values as local statistics. The analysis for **Fig. 2 C** was performed with default settings, whereas LION-terms to be considered were limited to all child-terms of 'contains fatty acid' (CAT:0000100) for **Fig. 3 A** and all child-terms of 'chain-melting transition temperature' (CAT:0001734) and 'lateral diffusion' (CAT:0080950) for **Fig. 3 D**.

Software and R-packages

All R-scripts were run with RStudio v1.0.153 (R v3.4.4) with the following packages: 'shiny v1.1.1', 'visNetwork v2.0.1', 'data.table v1.10.4-2', 'GMD v0.3.3', 'igraph v1.0.1', 'reshape2 v1.4.2', 'ggplot2 v2.2.1', 'ggthemes v3.4.0', 'shinyTree v0.2.2', 'shinyWidgets v0.4.1', 'shinythemes v1.1.1', 'RSQLite v2.1.1', 'topOnto v0.99.0', 'pheatmap v1.0.10' and 'xcms v2.99.3' [31]. Perl-scripts provided with the topOnto package were run with Perl v5.26.0. All figures were built in R and processed in Cytoscape v3.5.1 or Inkscape v0.92.2.

Data and code availability

The LION database (OBO-format) and raw lipidomics data are available as Supplementary Data. The public RAW 264.7 macrophages dataset [13] is available on the journal's website (http://www.jlr.org/content/suppl/2010/06/23/jlr.M008748.DC1/jlr.M008748-1.xls). R-package 'topOnto' is available at https://github.com/hxin/topOnto, the associated R-package

containing the LION database in topOnto-friendly format at https://github.com/martijnmolenaar/topOnto.LION2.db. The source code of the web-tool is available via github; Project name: LION-web; Project home page: https://github.com/martijnmolenaar/LION-web/ ; Operating system(s): platform independent; Programming language: R; License: GNU General Public License v3.0

ACKNOWLEDGEMENTS

We thank Xin He, PhD, for providing and supporting the topONTO R-package. We thank Jeroen W.A. Jansen for the excellent technical assistance with the lipidomics experiments.

AUTHOR CONTRIBUTIONS

M.R.M. and J.B.H. conceived the project. M.R.M. developed LION, LION/web and performed the experiments. A.J. tested and suggested improvements for LION/web. C.H.A.v.d.L. and T.A.W. contributed to the regression models and statistical concepts. C.H.A.v.d.L. and J.F.B. contributed to the lipidomics data processing and analysis. M.R.M. and J.B.H. wrote the manuscript.

COMPETING FINANCIAL INTERESTS

The authors declare no competing financial interests.

ADDITIONAL MATERIAL

Figure S1. LION-terms associated with PS(34:2).

Figure S2. Model validations of biophysical properties in LION.

Figure S3. Lipidomics of CHO-k1 cells incubated with free fatty acids.

Figure S4. Lipidomics of CHO-k1 cells incubated with arachidonic acid (AA).

Supplementary Data 1. XLSX-file containing all LION-terms excluding lipids with classification rules and sources.

Supplementary Data 2. CSV-file containing all lipids present in LION.

Supplementary Data 3. CSV-file with lipid clusters.

Supplementary Data 4. CSV-file with lipidomics dataset supporting Figure 2D.

Supplementary Data 5. CSV-file with LION/web output values supporting Figure 2D.

Supplementary Data 6. CSV-file with lipidomics dataset supporting Figure 2A.

Supplementary Data 7. CSV-file with LION/web output values supporting Figure 2A.

Supplementary Data 8. XLSX-file containing the coefficients of the biophysical models.

Supplementary Data 9. CSV-file with test-set for lipid names conversion.

Supplementary File 1. LION-database in OBO-format.

ABBREVIATIONS

LION: lipid ontology; CG-MD: coarse-grain molecular dynamics simulation; LOOCV: leave-one-out cross-validations; GO: gene ontology; PUFA: polyunsaturated fatty acid; PA: palmitic acid; LA: linoleic acid; AA: arachidonic acid; BSA: bovine serum albumin; LC-MS/MS: liquid chromatography – tandem mass spectrometry; PDA: pyrene decanoic acid; CSV: comma separated values

REFERENCES

[1] M. Ashburner, C. A. Ball, J. A. Blake, D. Botstein, H. Butler, J. M. Cherry, A. P. Davis, K. Dolinski, S. S. Dwight, J. T. Eppig, M. A. Harris, D. P. Hill, L. Issel-Tarver, A. Kasarskis, S. Lewis, J. C. Matese, J. E. Richardson, M. Ringwald, G. M. Rubin, and G. Sherlock, "Gene ontology: tool for the unification of biology. The Gene Ontology Consortium.," *Nat. Genet.*, vol. 25, no. 1, pp. 25–9, May 2000.

[2] K. Degtyarenko, P. de Matos, M. Ennis, J. Hastings, M. Zbinden, A. McNaught, R. Alcántara, M. Darsow, M. Guedj, and M. Ashburner, "ChEBI: a database and ontology for chemical entities of biological interest.," *Nucleic Acids Res.*, vol. 36, no. Database issue, pp. D344-50, Jan. 2008.

[3] H. J. Sharpe, T. J. Stevens, and S. Munro, "A comprehensive comparison of transmembrane domains reveals organelle-specific properties.," *Cell*, vol. 142, no. 1, pp. 158–69, Jul. 2010.

[4] M. E. Inda, M. Vandenbranden, A. Fernández, D. de Mendoza, J.-M. Ruysschaert, and L. E. Cybulski, "A lipid-mediated conformational switch modulates the thermosensing activity of DesK.," *Proc. Natl. Acad. Sci. U. S. A.*, vol. 111, no. 9, pp. 3579–84, Mar. 2014.

[5] E. Sezgin, I. Levental, S. Mayor, and C. Eggeling, "The mystery of membrane organization: composition, regulation and roles of lipid rafts," *Nat. Rev. Mol. Cell Biol.*, vol. 18, no. 6, pp. 361–374, Mar. 2017.

[6] K. Ben M'barek, D. Ajjaji, A. Chorlay, S. Vanni, L. Forêt, and A. R. Thiam, "ER Membrane Phospholipids and Surface Tension Control Cellular Lipid Droplet Formation.," *Dev. Cell*, vol. 41, no. 6, p. 591–604.e7, Jun. 2017.

[7] A. R. Thiam, R. V Farese, and T. C. Walther, "The biophysics and cell biology of lipid droplets.," *Nat. Rev. Mol. Cell Biol.*, vol. 14, no. 12, pp. 775–86, Dec. 2013.

[8] J. Bigay, P. Gounon, S. Robineau, and B. Antonny, "Lipid packing sensed by ArfGAP1

couples COPI coat disassembly to membrane bilayer curvature.," *Nature*, vol. 426, no. 6966, pp. 563–6, Dec. 2003.

[9] G. Enkavi, H. Mikkolainen, B. Güngör, E. Ikonen, and I. Vattulainen, "Concerted regulation of npc2 binding to endosomal/lysosomal membranes by bis(monoacylglycero)phosphate and sphingomyelin," *PLOS Comput. Biol.*, vol. 13, no. 10, p. e1005831, Oct. 2017.

[10] E. Fahy, S. Subramaniam, R. C. Murphy, M. Nishijima, C. R. H. Raetz, T. Shimizu, F. Spener, G. van Meer, M. J. O. Wakelam, and E. A. Dennis, "Update of the LIPID MAPS comprehensive classification system for lipids.," *J. Lipid Res.*, vol. 50 Suppl, no. Supplement, pp. S9-14, Apr. 2009.

[11] D. Marsh, "Structural and thermodynamic determinants of chain-melting transition temperatures for phospholipid and glycolipids membranes.," *Biochim. Biophys. Acta*, vol. 1798, no. 1, pp. 40–51, Jan. 2010.

[12] T. A. Wassenaar, H. I. Ingólfsson, R. A. Böckmann, D. P. Tieleman, and S. J. Marrink, "Computational lipidomics with insane: A versatile tool for generating custom membranes for molecular simulations," *J. Chem. Theory Comput.*, vol. 11, no. 5, pp. 2144–2155, May 2015.

[13] A. Y. Andreyev, E. Fahy, Z. Guan, S. Kelly, X. Li, J. G. McDonald, S. Milne, D. Myers, H. Park, A. Ryan, B. M. Thompson, E. Wang, Y. Zhao, H. A. Brown, A. H. Merrill, C. R. H. Raetz, D. W. Russell, S. Subramaniam, and E. A. Dennis, "Subcellular organelle lipidomics in TLR-4-activated macrophages.," *J. Lipid Res.*, vol. 51, no. 9, pp. 2785–97, Sep. 2010.

[14] R. A. Haraszti, M.-C. Didiot, E. Sapp, J. Leszyk, S. A. Shaffer, H. E. Rockwell, F. Gao, N. R. Narain, M. DiFiglia, M. A. Kiebish, N. Aronin, and A. Khvorova, "High-resolution proteomic and lipidomic analysis of exosomes and microvesicles from different cell sources," *J. Extracell. Vesicles*, vol. 5, no. 1, p. 32570, Jan. 2016.

[15] M. S. Köberlin, B. Snijder, L. X. Heinz, C. L. Baumann, A. Fauster, G. I. Vladimer, A. C. Gavin, and G. Superti-Furga, "A Conserved Circular Network of Coregulated Lipids Modulates Innate Immune Responses," *Cell*, vol. 162, no. 1, pp. 170–183, Jul. 2015.

[16] L. Lin, Y. Ding, Y. Wang, Z. Wang, X. Yin, G. Yan, L. Zhang, P. Yang, and H. Shen, "Functional lipidomics: Palmitic acid impairs hepatocellular carcinoma development by modulating membrane fluidity and glucose metabolism.," *Hepatology*, vol. 66, no. 2, pp. 432–448, Aug. 2017.

[17] A. Alexa and J. Rahnenfuhrer, "Gene set enrichment analysis with topGO," *Bioconductor*, 2017.

[18] W. T. Barry, A. B. Nobel, and F. A. Wright, "Significance analysis of functional categories in gene expression studies: a structured permutation approach.," *Bioinformatics*, vol. 21, no. 9, pp. 1943–9, May 2005.

[19] J. C. M. Holthuis and A. K. Menon, "Lipid landscapes and pipelines in membrane homeostasis.," *Nature*, vol. 510, no. 7503, pp. 48–57, Jun. 2014.

[20] G. van Meer, D. R. Voelker, and G. W. Feigenson, "Membrane lipids: where they are and how they behave.," *Nat. Rev. Mol. Cell Biol.*, vol. 9, no. 2, pp. 112–24, Feb. 2008.

[21] P. C. Calder, "Marine omega-3 fatty acids and inflammatory processes: Effects, mechanisms and clinical relevance.," *Biochim. Biophys. Acta*, vol. 1851, no. 4, pp. 469–84, Apr. 2015.

[22] X. Yang, W. Sheng, G. Y. Sun, and J. C. M. Lee, "Effects of fatty acid unsaturation numbers on membrane fluidity and α-secretase-dependent amyloid precursor protein processing.," *Neurochem. Int.*, vol. 58, no. 3, pp. 321–9, Feb. 2011.

[23] J. Eisinger and S. F. Scarlata, "The lateral fluidity of erythrocyte membranes temperature and pressure dependence," *Biophys. Chem.*, vol. 28, no. 3, pp. 273–281, Dec. 1987.

[24] D. Schwudke, A. Shevchenko, N. Hoffmann, and R. Ahrends, "Lipidomics informatics

for life-science," *J. Biotechnol.*, vol. 261, pp. 131–136, Nov. 2017.

[25] L. Aimo, R. Liechti, N. Hyka-Nouspikel, A. Niknejad, A. Gleizes, L. Götz, D. Kuznetsov, F. P. A. David, F. G. van der Goot, H. Riezman, L. Bougueleret, I. Xenarios, and A. Bridge, "The SwissLipids knowledgebase for lipid biology.," *Bioinformatics*, vol. 31, no. 17, pp. 2860–6, Sep. 2015.

[26] A. I. P. M. de Kroon, P. J. Rijken, and C. H. De Smet, "Checks and balances in membrane phospholipid class and acyl chain homeostasis, the yeast perspective.," *Prog. Lipid Res.*, vol. 52, no. 4, pp. 374–94, Oct. 2013.

[27] S. J. Marrink, A. H. de Vries, and A. E. Mark, "Coarse Grained Model for Semiquantitative Lipid Simulations," *J. Phys. Chem. B*, vol. 108, no. 2, pp. 750–760, 2004.

[28] T. Wächter and M. Schroeder, "Semi-automated ontology generation within OBO-Edit.," *Bioinformatics*, vol. 26, no. 12, pp. i88-96, Jun. 2010.

[29] E. G. Bligh and W. J. Dyer, "A rapid method of total lipid extraction and purification," *Can. J. Biochem. Physiol.*, vol. 37, no. 8, pp. 911–917, Aug. 1959.

[30] R. Adusumilli and P. Mallick, "Data Conversion with ProteoWizard msConvert.," *Methods Mol. Biol.*, vol. 1550, pp. 339–368, 2017.

[31] C. A. Smith, E. J. Want, G. O'Maille, R. Abagyan, and G. Siuzdak, "XCMS: processing mass spectrometry data for metabolite profiling using nonlinear peak alignment, matching, and identification.," *Anal. Chem.*, vol. 78, no. 3, pp. 779–87, Feb. 2006.

[32] J. Xia, I. V Sinelnikov, B. Han, and D. S. Wishart, "MetaboAnalyst 3.0--making metabolomics more meaningful.," *Nucleic Acids Res.*, vol. 43, no. W1, pp. W251-7, Jul. 2015.

FIGURE LEGENDS

Figure 1. Enrichment analysis approaches supported by LION/web. A lipidomics dataset containing lipid identifiers and abundances derived from two or more conditions (❶) can be processed by LION/web in two ways. In the 'target-list mode' (left, ❷), a subset of lipids (*e.g.*, derived from thresholding or clustering) is compared to the total set of lipids. After standardization of lipid nomenclature (❸), applicable LION-terms are associated and assessed for enrichment in the subset by Fisher's exact statistics. In the 'ranking mode', input lipids are ranked by numeric values ('local' statistics) (❷). After ranking, lipid nomenclature is standardized (❸). Applicable LION-terms are subsequently associated to the dataset and distributions are compared to a uniform distribution by 'global' statistics (here, Kolmogorov–Smirnov tests). Calculated *P* values of LION-terms from both modes are corrected for multiple testing (Benjamini-Hochberg).

Figure 2. LION-term enrichment analysis of RAW 264.7 macrophages. **(A)** Heatmap of scaled lipid amounts (z-score < 0: blue, z-score > 0: red) of subcellular lipidomics data [13] with samples on the x-axis and individual lipid species on the y-axis. Lipids were clustered into 10 groups by hierarchical clustering. **(B)** Enrichment analyses of all lipid clusters in the 'target-list mode'. For each cluster, the first *n* + 2 significant LION-terms are shown. **(C)** Enrichment analysis of PM *vs.* ER fractions in the 'ranking mode'. The gray vertical lines indicate the cut-off value of significant enrichments ($q < 0.05$). Bar colors are scaled with the enrichment (-log *q*-values).

Figure 3. LION-term enrichment and membrane fluidity of CHO-k1 cells. CHO-k1 cells were incubated overnight with PA, LA or AA (100 µM) **(A)** or with AA (250 µM) **(B-D)**. All incubations were performed in triplicate. For control incubations, cells were incubated with fatty-acid free BSA. **(A,D)** After extraction and lipidomics profiling by LC-MS/MS, enrichment analyses of the conditions of interest versus control incubations were performed by LION/web of **(A)** LION-terms indicating the presence of selected fatty acids or **(D)** LION-terms indicating the degree of membrane fluidity. Dot sizes in the dot plots are scaled to the number of associated lipids; colors are scaled to the level of enrichment (-log *q*-values). **(B,C)** After incubation, fluorescence emission spectra of lysates containing pyrenedecanoic acid (PDA) were measured **(B)**. Fluorescence spectra examples of either control (black) or AA-stimulated lysates (red). Gray shades indicate monomer and excimer fluorescence filters. **(C)** Mean ratios (bar) and individual datapoints (dots) of excimer over monomer fluorescence (representative data of three independent experiments). Statistical significance was determined by Student's two-tailed t-test. **(A,C,D)** * P or $q < 0.05$, ** P or $q < 0.01$, *** P or $q < 0.001$.

Click here to access/download;Figure;Figure 1.png

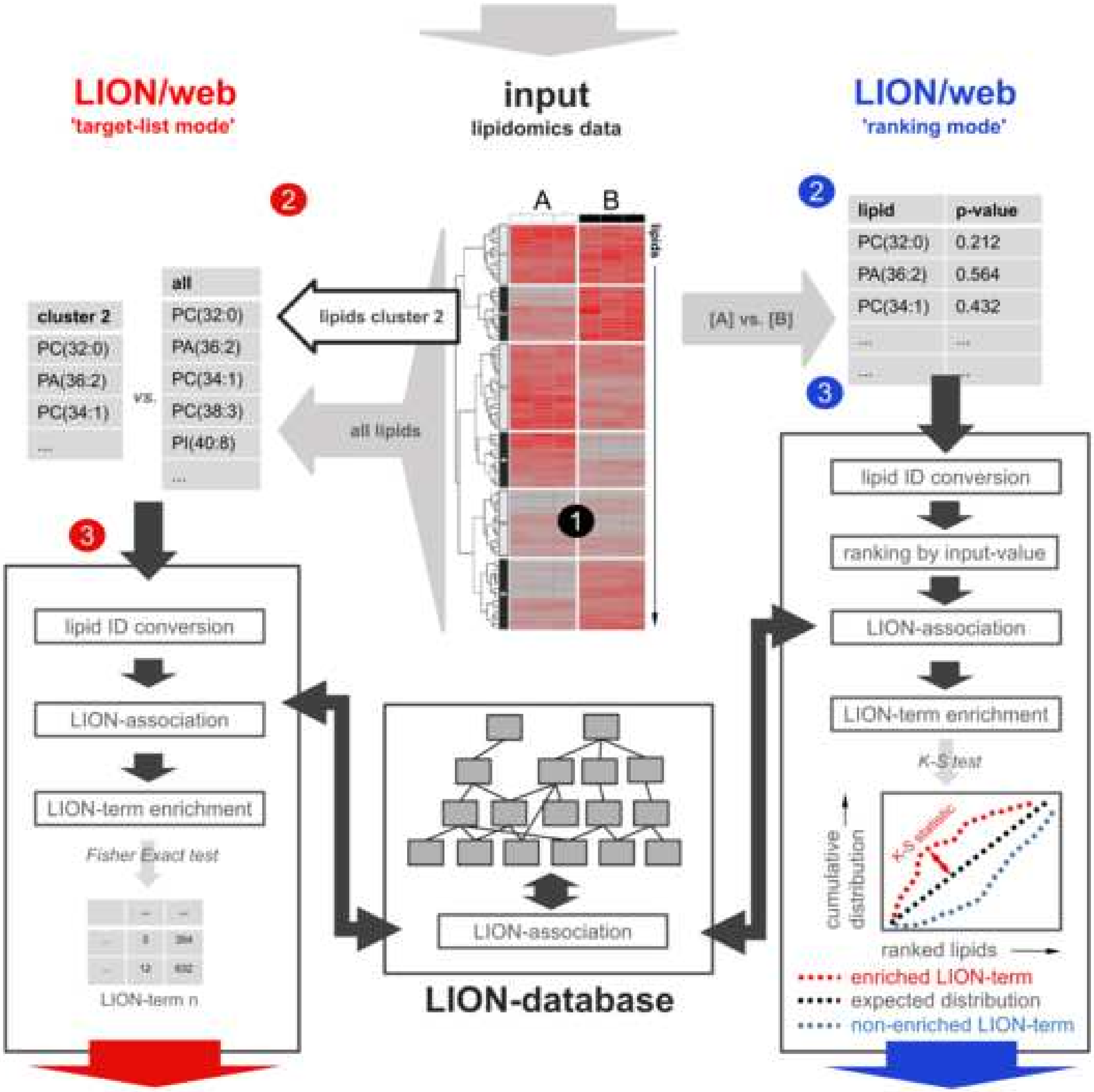

Figure 2

Click here to access/download;Figure;Figure 2.png

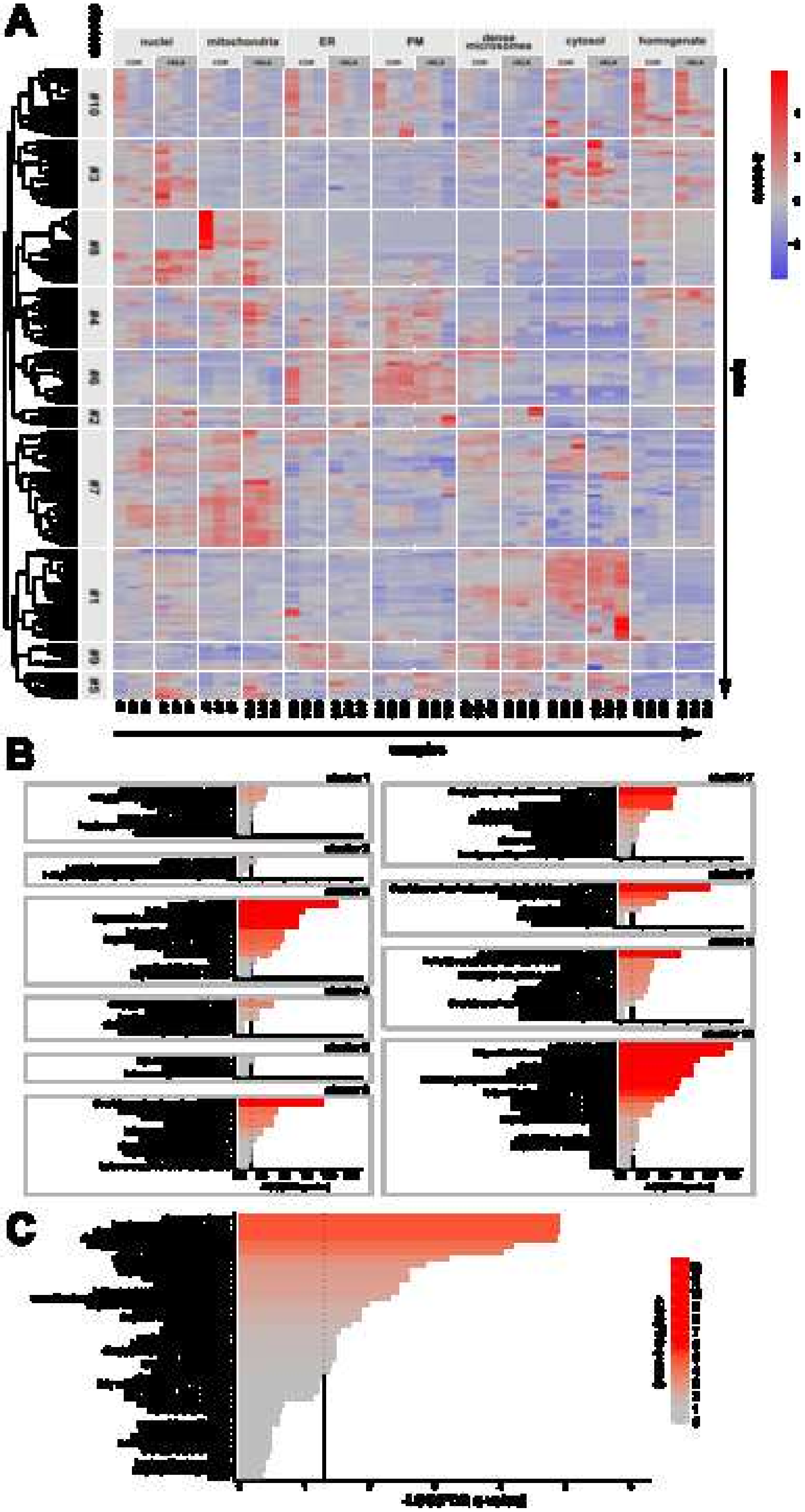

Figure 3 Click here to access/download;Figure;Figure 3.png

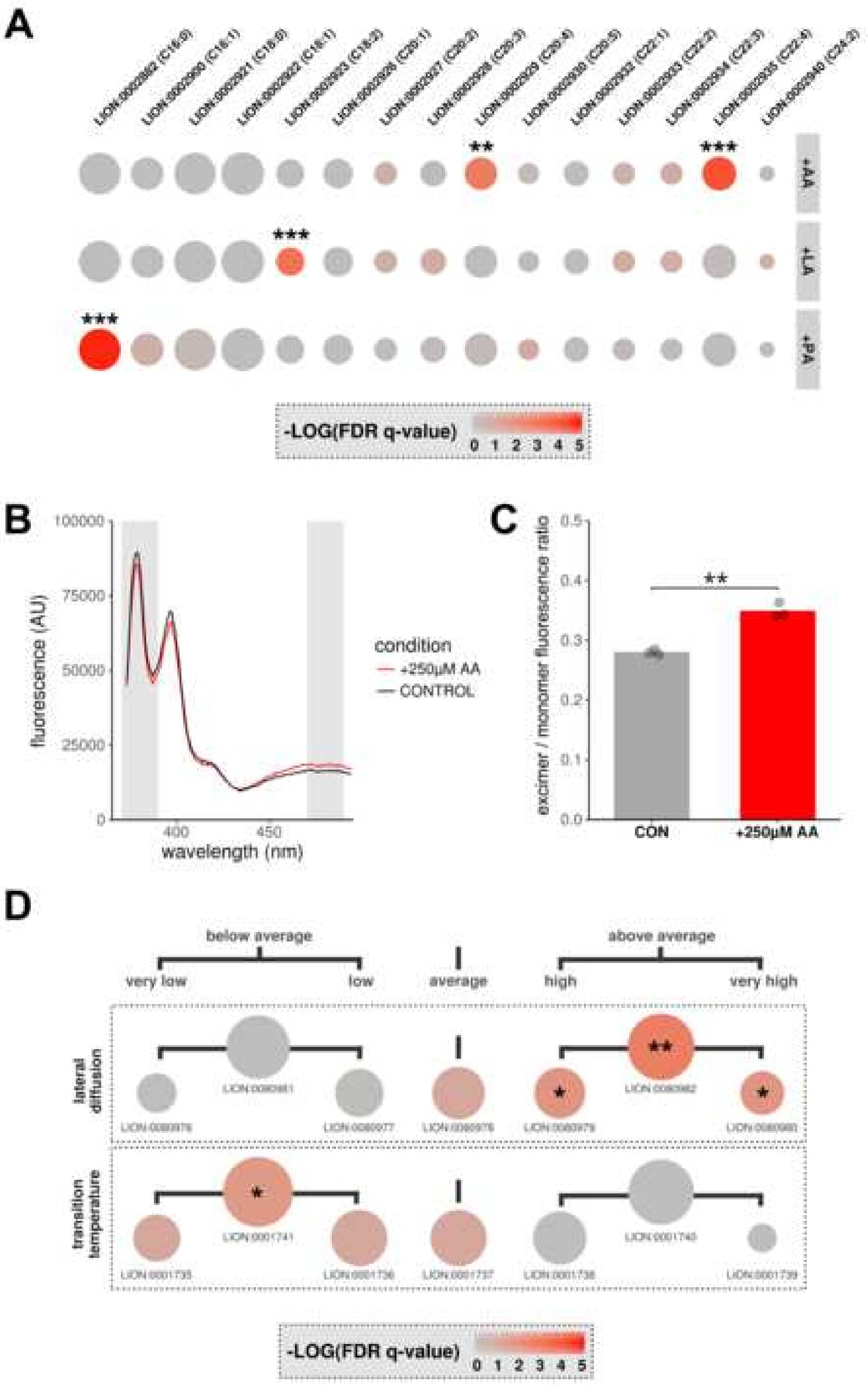

Suppl. Fig. 3

Click here to access/download
Supplementary Material
Figure S3.png

Supplementary text

Click here to access/download
Supplementary Material
20190226 LION Supplement_after review final.docx

Supplementary data 1

Click here to access/download
Supplementary Material
Supplemental Data 1.xlsx

Supplementary data 2

Click here to access/download
Supplementary Material
Supplemental Data 2.csv

Supplementary data 3

Click here to access/download
Supplementary Material
Supplemental Data 3.csv

Supplementary data 4

Click here to access/download
Supplementary Material
Supplemental Data 4.csv

Supplementary data 5

Click here to access/download
Supplementary Material
Supplemental Data 5.csv

Supplementary data 6

Click here to access/download
Supplementary Material
Supplemental Data 6.csv

Supplementary data 7

Click here to access/download
Supplementary Material
Supplemental Data 7.csv

Supplementary data 8

Click here to access/download
Supplementary Material
Supplemental Data 8.xlsx

Supplementary data 9

Click here to access/download
Supplementary Material
Supplemental Data 9.csv

Supplementary File 1

Click here to access/download
Supplementary Material
Supplemental File 1 LION.obo

Cover Letter revised manuscript Molenaar et al. Click here to access/download;Personal Cover;Cover Letter Molenaar et al revised version GIGA-D-18-00357.pdf

Universiteit Utrecht

GigaScience
The Editor
Nicole Nogoy, Ph.D

Faculty of Veterinary Medicine
Department of Biochemistry
and Cell Biology
PO Box 80176
3508 TD Utrecht
The Netherlands
Tel: +31-30-2535375
Fax: +31-30-2535492
Email: j.b.helms@uu.nl

February 27, 2019

Dear Nicole Nogoy,

Please find enclosed our revised manuscript GIGA-D-18-00357 entitled "LION/web: a web-based ontology enrichment tool for lipidomic data analysis" by Martijn Molenaar et al. that we would like to resubmit for publication in your journal GigaScience.

Attached you will find a Point-by-Point answer to the comments made by the reviewers. We have also included a revised version of the manuscript with the changes highlighted. Most important changes include the incorporation of an additional figure to illustrate the 'target-list mode' and a strong improvement of the description and explanation of the database and webtool.

We thank the reviewers for their helpful comments that have helped to improve the quality of this manuscript.

We hope you find the manuscript now suitable for publication in GigaScience.
With kind regards

Bernd Helms

Prof. Dr. J.B. Helms

Response to Reviewers Click here to access/download;Personal Cover;20190227 Rebuttal.doc

Ruth Welti (Reviewer 1)

LION provides useful information helping users associate lipidomics data on membrane lipid species from mammalian systems with the chemical and physical properties of those systems. Overall this is an ambitious undertaking that is likely to provide insights on lipid properties, particularly to users that are not familiar with chemical or physical properties of membrane lipids. Overall, the tool seems useful and the paper is well-written, but a few points could be explained in more detail.

We appreciate the positive and constructive comments of the reviewer.

#1. It should be mentioned, and perhaps the authors could include an explanatory note at the site, noting that actual physical properties of membranes (such as fluidity) depend on factors in addition to the typically measured lipids, including sterols and protein type and content.

We incorporated a statement about this aspect at three different locations: i) in the web-tool (on the '?' sign, beneath the results output); ii) in a new F.A.Q. that is now available via the website; and iii) in the Discussion (line 200-204).

#2. It might be useful to point out specifically that the samples chosen to "calibrate" the lipid categorization are all from mammalian cells and thus the ability to accurately interpret lipidomics data from other types of systems is not clear. Perhaps this is because it is not clear to the reviewer precisely how the categorized lipids (page 4, lines 69-74) were used in the analysis. Since many mammalian tissues (e.g. brain, heart) have more extreme compositions, will this be a problem for analysis?

Indeed, we made use of mammalian lipidomics datasets as reference to define the groups of three biophysical properties. To emphasize this, we included a comment on LION's focus on mammalian lipidomes in the Discussion (line 193-197). This will, however, not compromise the results in specific examples as mentioned by the reviewer as the principle of LION/Web is based on sample comparison (Fig.1, sample A and B). A comparison between tissues with more extreme compositions (e.g. brain and liver) is likely to result in enriched terms related to very low T_m's or very high lateral diffusions, and in different lipid classes/species, results that reflect the respective lipidomes. Comparison between samples from the same tissue (e.g. wt brain vs. geneX$^{-/-}$ brain) will often yield more subtle differences, depending on the knockout. However, LION/Web will report any significant difference, *e.g.* if geneX affects lipid composition. The statistical power of the significance can be further increased by increasing the number of replicates (n).

#3. The ranking approach appears to be a pairwise comparison. I.e., even when multiple samples are present, comparison is to one (control) sample. This is analogous to a typical transcriptomic approach but, given that it's actually easier to collect lipidomic data than transcriptomic data on hundreds of samples/conditions, having to analyze the data pairwise might be a bit burdensome. Maybe you could discuss the choice of approach in the paper or clarify if the reviewer's understanding is incorrect.

We thank the reviewer for this comment. We have extended the web-tool with more options to calculate local statistics (values that are used to rank lipids). One of these options is the use of p-values derived from one-way ANOVA F-tests. This statistic analysis allows comparison of multiple conditions and can be used to rank the most fluctuating lipid species in datasets. Subsequent enrichment analysis will result in LION-terms summarizing these lipids.
A second option that we included to characterize lipidomic datasets with more than 2 conditions, is the use of hierarchical clustering in combination with the target-list mode. A new figure (Figure 2B)

illustrates this approach using the same public dataset that we used in the initial version of the manuscript. Enrichment analysis of the lipids in the clusters, in combination with a visual presentation of the clusters in relation to the conditions, further aids in characterization of the full dataset.

#4. An example showing the output from the target mode would be helpful to the reader.

We agree with the reviewer that the manuscript would benefit from an example of the target mode. As mentioned above (#3) we now include a new figure (Figure 2B) that shows a clustered heat map of the RAW 264.7 macrophages dataset. Each cluster is characterized by assessing LION-term enrichment of the lipids within each cluster, as compared to all the lipids in the experiment.

Aleksander Andreyev (Reviewer 2)

This technical note describes a LIpid-related ONtology database (LION) and accompanying enrichment analysis tool with potentially high value for lipidomics research. According to the authors they aim to "bridge<> the gap between lipidomics and cell biology" (p.7, l.138). A mere attempt at this herculean task is highly commendable. This entails, however, that the narration should be comprehensible for a non-expert user, presumably a cell biologist with little understanding of bioinformatics (which would be also in line with the GigaScience editorial guidelines). Unfortunately, the manuscript is plagued with multiple issues that make it very hard to understand the utility and intended use of the tools and nearly impossible to evaluate their validity. From the way manuscript is written, it feels as if it is intended more for bioinformatics audience which almost defeats the purpose. It is also somewhat disorganized with the logical flow being interrupted by off-hand remarks and description of one topic spread over different parts of the manuscript, sometimes repetitively. In a few cases, the text is burdened with statements of the obvious (e.g., "lipid structure is closely related to lipid function", "allows identification of lipid-associated terms in lipidomes"). There are multiple typos, grammar errors and misused words or terms that make a mere reading of the article a torture. One step to address this issues might be including subsections under the Findings section, another - careful reassessment of what material represents technical side and belongs to Methods and what should be in the Findings (my feeling is that a good portion of the LION description, currently under Methods, actually belongs to the Findings, right after the background information). The same goes to figure legends - I think currently they are overloaded with information that belongs in the Methods. The manuscript suffers from frequent use of vague statements. Instead of describing WHAT was done the authors simply state the means for doing it: "we used" this or that, "we made use of" this or that, such and such "was used", etc. Instead of explaining HOW something was done a bare statement "based on" is often made. References are missing (e.g., "as described in the literature", p.4, l.53, "was reported", p.5, l.99). The tally of connections between membrane biophysics and cell biology (p.3, l.35-43) looks random and lacking completeness. Besides, it seems somewhat misplaced. Authors use what appears to be in-house or jargon terms, such as "by target list", "by ranking" for the modes of the enrichment analysis, "local" statistics, etc. Use of such terms should be avoided. For such important terms as the modes of analysis the names should be related to their function and, ideally, self- explanatory (or, at least, thoroughly explained). All these issues pertaining to the quality of the narration should be addressed before the substance of the work can be properly evaluated.

We thank reviewer #2 for his thorough review report and would like to apologize for the typos and grammar errors in the manuscript that made '*reading of the article a torture*'. As suggested, the Findings section is now subdivided into subsections with headings. We also include a separate Discussion section to avoid the 'interruption by off-hand remarks'.

Indeed, LION/web is intended to be useful for non-experts in bioinformatics. We recognize that some concepts used in the manuscript might be difficult to grasp with limited bioinformatics experience. Nevertheless, some basic understanding of data-analysis must be expected from users that obtained omics-data (which is obviously a prerequisite to use LION/web). In the updated version, we have provided more explanation and illustrate some of the concepts with examples in the following ways:
i) throughout the manuscript, we added additional information.
ii) we added a point-by-point frequently asked question (F.A.Q.) section in the web-tool, that can be accessed via the main menu of the website.

iii) we added ‘tooltips’ in the LION/Web application. Tooltips are pieces of information or instructions that appear when users hover the mouse cursor over an item - without clicking on it. This allows for specific instructions for specific steps.
Upon the reviewer’s request, we have considered several alternative names for the enrichment modes (ranking and target-list mode). However, we found the initial names to be the clearest, as it describes the difference between the modes the best. The use of a target-list (usually referred to as gene list, ID list, etc.) is also common practise in gene ontology enrichment procedures (DAVID, Panther, GOrilla). Users who have experience in this field will recognize the concept ‘target-list’. To improve the understanding of these terms/modes, we included more details about both modes in the Methods section. In addition, we added a new figure (Figure 2A+B) to illustrate the target-list mode.

With respect to the comment *“The tally of connections between membrane biophysics and cell biology (p.3, l.35-43) looks random and lacking completeness”* we note that the listing of biophysical properties related to membrane biology in the background section was not intended to be complete, but to provide a few intuitive examples. To clarify this, we put these examples in parentheses and ‘e.g.’.

Concerning missing references: Details about references per data source is now available via Supplemental Data 1. The statement ‘*was reported*’ (page-5/line-99 of initial manuscript) refers to LION-terms that were reported by the web-tool.

However, even in the present state the manuscript allows to point out the following weaknesses/areas for improvement:

#1. The LION should be completely verbally described (beyond the present reference to the .obo file). This should include a list of categorical ontology terms and rules of association between them. For the ones that are not obvious, a justification should be provided. As it stands now, the terms in question are hidden inside 1275-page long Excel file among about 50,000 terms representing individual lipids. Some of them relate to conventional structural elements of lipids, others are less obvious. For example, "fatty acid with 16-18 carbons" - is there any scientific meaning in this term? What is so special about this particular chain length? What exactly are the extra levels of classification between lipid classes and species? - they are mentioned but not described.

Upon the reviewer’s request, we describe LION in a better structured way by inclusion of two additional tables:
(1) Supplemental Data 1; describing all LION-terms (excluding lipid species), with detailed information about hierarchy, classification and references.
(2) Supplemental Data 2; describing all lipid species present in LION.

Concerning the scientific meaning of terms: one of the guiding principles of LION was to be able to construct defined subsets of lipids (‘terms’). LION/web then aids to report the most interesting subsets. Some of these subsets might be of interest, others might not. Scientific meaning should be evaluated by the scientist. For example, "*fatty acid with 16-18 carbons*" might indeed sound trivial at the first sight. Nevertheless, its enrichment could hint towards testable biological hypotheses.

#2. The enrichment tool is the crux of the article, the thing the authors are trying to "sell". However, there is no description of what it does and how it can be used. I flatter myself to be a qualified used but I could not make a head or tail of what the so called "by target list" mode does. If my "target list" includes unsaturated lipids I'll get enrichment in "double bonds", "below average transition temperature", etc. That much is obvious without running the tool. What else? What are the scenarios

when I need to use it? Why do I need two lipidomic data sets for this? What does "derived from thresholding or clustering" mean?

We recognize that in the initial version of the manuscript, the use of the 'target-list mode' was not illustrated. We added an extra figure (figure 2) that demonstrates the use of both modes using the RAW 264.7 macrophages dataset (figure 2A+B for the target-list, figure 2C for the ranking mode). Figure 2C was a supplemental figure in the original manuscript.

The second mode, apart from the name (why "by ranking"? isn't this purely technical approach to facilitate stat analysis?), is less problematic. However, the option to limit analysis to a specific set of terms ("terms of interest") should be mentioned upfront. Then, the questions arise in what scenarios this would be advantageous? Would this create a bias in the analysis or not, both with regard to outcome and its stat significance?

We now describe the selection of specific sets at an earlier stage.

#3. The claim of the scope is overreaching. The "function" category, most interesting for cell biology researchers, appears to be extremely frugal, limited just to the crudest distinction between structural, signaling and storage functions. If this perception is correct, the LION would be of limited value for cell biology. The "chemical" properties appear to be a misnomer with chemical information limited purely to structural elements with no regard to reactivity, biochemical synthetic pathways, etc. I would say that, according to this Technical Note, the LION is the ontology linking lipidomics data to biophysical properties of corresponding membranes. The testing of the ontology was performed in a set of assays pertaining to membrane biophysics.

We found a single occurrence of 'cell biology' (*'...web-tool bridges the gap between lipidomics and cell biology...'*) in the initial manuscript. This claim is now phrased with greater caution by *'... future expansions of the LION database..., LION/web will be increasingly successful to bridge the gap between lipidomics and cell biology.'* (line 216-218). However, we believe that besides 'function', also 'cellular component' and the biophysical properties are of interest for scientists studying cell biology. In addition, we will maintain the LION database and update it when new lipid data and functions of individual lipid species or classes become available (see also our reply to the comment of the expert editorial board member).

#4. It would be advantageous to sync terminology with other ontologies whenever possible, for example, use the GO term "cellular component" instead of "cellular localization", etc. "Lipid component" is a very dubious term for a structural lipid.

As suggested, we replaced the LION-term name "cellular localization" by "cellular component". "Lipid component" was a typo in the manuscript, and not the name of a term in LION. We apologise for this mistake.

#5. The biophysical properties of the vast majority of lipids were inferred from a limited set of literature data. It is therefore of utmost importance to thoroughly describe the approach used. What kind of data the sources provided? Where they for individual lipids or mixes, measured or calculated? How many entries? The equations for the multiple linear (sic!) regression analysis should be shown. The resulting coefficients could be of value by itself - why not publish them here?

We thank the reviewer for noticing the missing word 'linear'. We replaced multiple occurrences of 'multiple regression analysis' by 'multiple linear regression analysis'.

As mentioned earlier, we now include a supplemental table with data sources per LION-term. The raw numeric values (per lipid) of the biophysical properties derived from these sources were already provided together with the original manuscript in 'scripts' folder. It is our understanding that this folder is available to the reviewers (and to the public after publication).

We appreciate the suggestion to report the coefficients of the models. To this end, we now include an Excel spreadsheet containing the coefficients of the models, together with input cells to predict (numerical) values of the biophysical properties (Suppl. Data 8).

#6. The lipids appear to be divided into "quintiles" using a hard-to-describe (and almost lacking description in the manuscript) procedure based initially on a number of lipids in each group rather than the value of a biophysical parameter. What is the rationale for this? Does transition temperature of a lipid membrane care how many other membranes share the same value? I think the categorization should be based upon the magnitudes of biophysical properties alone. By the way, how many groups are actually there? The text says 5 but Fig. 2 shows 7... Also, Fig. 2 shows FDR q-values which are not mentioned in either legend or the main text.

We categorized 'transition temperature' into 5 groups: very low, low, etc. These descriptions are not defined and intrinsically subjective: whether a membrane has a low T_m depends on the context. To provide this context, we selected four lipidomics studies to serve as reference. Lipids from these reference lipidomes were ranked based in the predicted numeric values of the biophysical property. Then, the first 20% (first quintile) was defined as 'very low', the second 20% (second quintile) as 'low', etc. The limits of these quintiles were then used to classify all lipids present in LION. We believe that this approach defines the group limits with more physiological relevance. The alternative approach, based on magnitudes of biophysical properties alone (as suggested by the reviewer) is more likely to yield a quintile 'average' for a group of non-physiological lipids.

The confusing of 5 groups vs. the 7 groups in figure 2 (now figure 3) is related to hierarchy. The groups 'very low ...' and 'low ...' are linked to a parental group called 'below average ...'. The same goes for 'high ...' and 'very high ...', they are linked to 'above average ...'. We updated the figure by adding a graphical representation of this hierarchy to the figure (new figure 3D). The hierarchy of LION-terms is also depicted in supplemental Data S1.

We now include a reference to 'q-values' in the figure legends.

#7. It is not absolutely clear from the manuscript but appears that the enrichment tool relies on the significance of the changes (p-value), as opposed to magnitude, to evaluate enrichment. Is this true? Is it possible that highly significant changes in low abundance lipids would dominate the outcome list without having much effect on the properties of membrane?

All enrichment analyses in the initial version of the manuscript used the ranking-mode with one-tailed t-test p-values to rank the lipids. Other statistical methods could be considered, but every choice has its pros and its cons. Magnitude (fold-change of condition B over condition A) has the undesirable property to overestimate effects when lipid concentrations are close to noise levels: it does not take sample variance into account. In contrast, p-values are more robust, but might be less intuitive to users without strong background in statistics. Using p-values, it is potentially possible that '*highly significant changes in low abundance lipids could dominate the outcome list*'. However, most low abundant lipids usually display higher variance due to lower signal/noise levels. As a result, they usually do not generate extreme low p-values.

To provide more flexibility for users and to make the choice of a local statistic explicit, we now offer three local statistics (one-tailed t-test p-values, ^{2}log fold-change, F-test p-values) in the updated version of the web-tool. The statistical method must be selected each time an analysis in the ranking mode is initiated.

#8. More detail should be provided on the statistics, for example, how the distribution curve was generated for K-S analysis, what were the input parameters for the Fisher exact test, etc.

We added more information in the Methods section.

#9. Methods for PDA assay and LC-MS should be brought to compliance with editorial guidelines to allow duplicate these studies. Missing are parameters such as cell number, concentration of the dye, shape of LC gradient, LC system used, MS/MS settings, to name a few. The full name of the Fusion mass spec should be provided because there are several different models. The text is not clear on the sequence of events: it sounds as if analyte ions fly from orbitrap to linear ion trap for detection - is this even possible?

We added details about the PDA in the manuscript.

The methods for LC-MS have now been described in greater detail to facilitate easy replication of experiments. Parallelization of MS1 and MS2 experiments has been clarified to avoid confusion. Current versions of the MS instrument are branded as 'Fusion Lumos' or 'Fusion IDX'. However, the original 'Orbitrap Fusion' mass spectrometer (serial number FSN10438) was branded under that name and this is the model used in our studies. Therefore, we cannot specify the type of instrument more accurately than we currently do.

#10. With regard to membrane fluidity data, although they show the desired differences they could be made much more convincing with appropriate controls subtracting intrinsic fluorescence of the cells.

The membrane fluidity data presented in the manuscript were subtracted from background fluorescence (blanks were samples with cells but without PDA dye). To make this clear, we updated the Methods section with this information.

#11. Annotating lipids with the "most abundant fatty acid composition" is misleading - if isobaric species are not resolved the overall composition (total carbons, total double bonds) should be shown as primary annotation (possibly followed by the most abundant isomer).

We now include the overall composition as primary annotation, together with a second column containing the most abundant isomer (Data S4). MS/MS analysis allows identification of the most abundant isomer (e.g. PC with a C18:1 and a C18:0 fatty acid) without assignment of the sn1/sn2 position of the respective fatty acids. It is important for experiments such as described in figure 3A to use identifiers containing individual fatty acids. LION-terms related to fatty acids cannot be associated to a dataset that lacks this information. To avoid confusion, we have renamed the lipid species from e.g. PC(18:1/18:0) to PC(18:1_18:0) to indicate the fatty acid composition of lipid species without sn1/sn2 assignment.

Expert editorial board comments on usability:

The following comments are thus from the perspective of a potential user.

Can the authors specify the source of the 50,000 lipid species included into the analyses? To my knowledge the lipidmaps database reports around 42,000 entries only.

We used the lipid classification system (hierarchy) in accordance with LIPIDMAPS. The individual lipid species in LION were constructed by combining lipid classes with abundant fatty acids. LIPIDMAPS is probably somewhat more stringent about the inclusion of lipid species in their database as they intend to provide additional information for individual species. We added a few lines (231-236) about the construction of individual lipid species in LION to the Methods section.

The number of lipid species linked to experimental or in silico data is more than two orders of magnitudes lower that the indicated number of 50,000 and mainly refers to membrane lipids. Are all of these 50,000 species associated with one or more than one feature? Can the authors comment how many of these 50,000 lipids are associated with features going beyond chemical properties? What kind of cell biological features were used and which of these features where linked to which lipid species? In order to understand and validate the assignments as more detailed description would be helpful.

Many lipids have a number of associations, whereas some lipids only have a few. As a consequence of the hierarchical structure of LION, lipids with only one association will not occur: lipids are (indirectly) associated with the neighbour's neighbour. To make this information more accessible for users, we improved the enrichment-report , which can be obtained by the button 'download report'. It now contains three files: a CSV-file with the enrichment information, a CSV-file containing all the LION-terms associated with the lipids in the dataset, and *vice versa*, a CSV-file containing all lipids of the dataset with associated LION-terms. With this information, users are better equipped to understand the underlying data structures and improve interpretation of obtained results.

Can the authors comment on why they integrated coarse-grain but not (in addition) atomistic MD data?

To our knowledge, there is no comprehensive lipid dataset available that has been obtained by atomistic molecular dynamics simulations. More importantly, the biophysical properties are categorized into distinct groups (very low, low, average, etc.). Given this categorization in groups, we suspect that the increased resolution of atomistic MD will be of no or very limited added value.

Can the authors specify which data of the two papers in particular was included into building the application?

We now provide a detailed supplemental table (Data S1) containing references per LION-term. Moreover, the source data and code are available via the script folder.

The fact that the application in its current form is restricted to glycerol-based lipids and fatty acids should be indicated in the abstract and in the discussion of the dataset.

We agree that the current LION database is not a complete end product. However, it is not true that LION only contains (associations to) glycerol-based lipids and fatty acids. The database includes many more: sphingolipids (sphingomyelins, ceramides, glycosphingolipids), cholesterol derivatives

and retinoids. As comprehensive biophysical data about these classes is hardly available or too complex in the case of cholesterol, not all these classes are associated with biophysical properties. The biophysical properties obtained by MD are limited to glycerol-based lipids. The transition temperatures are also associated with sphingomyelins. Cellular component, intrinsic curvature, headgroup charge are associated with many lipid classes. Limitations of LION/web are included in the Discussion section.

For this first version of LION the authors included only information from two publications. There is an increasing amount of data available going beyond this information. Can the authors comment on how they plan to allow for integration of additional information? Will users be able to do so in a ‘customized’ fashion?

We recognize the importance to involve users in the improvement of LION and LION/web. To this end, we added several features to the web-tool.
(i) We include an option (not selected by default for privacy reasons) that -when selected- informs us when lipids could not be matched to LION. This helps us to keep track of lipid identifiers that are often used, but not present in LION.
(ii) We include a contact form on the website to lower the threshold to contact us for questions, requests, suggestions or feedback.
Web-tool improvement will not stop after publication. Currently, we are working on features to build heat maps and principle component analyses within the web-tool. When new sources containing useful data become available, this will be added this to the database.

The power of application depends on the number of features associated with each lipid species. Can the authors comment on how they plan to advance the data base, e.g., by including the community? Will the application be hosted and if so, what is the perspective?

The full ontology, R-packages to perform enrichment analysis and R-code for the web-tool is publicly available. This is sufficient for experienced users to build customized versions of LION or the web-tool. We understand, however, that this will be challenging for inexperienced users. In the future, we plan to build a dedicated LION R-package with detailed instructions and guidelines to augment the ontology by individual users. An R-package provides more flexibility than a web-tool and the use of user-customized ontology versions will be easier to support.

The web-tool is currently hosted by Shinyapps.io. It will be hosted elsewhere in case this service discontinuous its operation. The domain name lipidontology.com is owned by the department and the web-tool LION/web will remain accessible via lipidontology.com.
