# Supplementary material for: LION/web: a web-based ontology enrichment tool for lipidomic data analysis
Source: Gigascience. 2019 May 29;8(6):giz061. doi: 10.1093/gigascience/giz061 (PMC6541037; doi:10.1093/gigascience/giz061)
Supplement: giz061_GIGA-D-18-00357_Revision_2 [file giz061_giga-d-18-00357_revision_2.pdf]

GigaScience

LION/web: a web-based ontology enrichment tool for lipidomic data analysis

--Manuscript Draft--

**Manuscript Number:**	GIGA-D-18-00357R2
**Full Title:**	LION/web: a web-based ontology enrichment tool for lipidomic data analysis
**Article Type:**	Technical Note
**Funding Information:**	BE-BASIC (BB/2012-10047) — Prof. dr. Bernd Helms
**Abstract:**	A major challenge for lipidomic analyses is the handling of the large amounts of data and the translation of results to interpret the involvement of lipids in biological systems. We built a new lipid ontology (LION) that associates over 50,000 lipid species to biophysical, chemical and cell biological features. By making use of enrichment algorithms, we used LION to develop a web-based interface (LION/web, www.lipidontology.com) that allows identification of lipid-associated terms in lipidomes. LION/web was validated by analyzing a lipidomic dataset derived from well-characterized sub-cellular fractions of RAW 264.7 macrophages. Comparison of isolated plasma membranes with the microsomal fraction showed a significant enrichment of relevant LION-terms including ‘plasma membrane’, ‘headgroup with negative charge, ‘glycerophosphoserines’, ‘above average bilayer thickness’, and ‘below average lateral diffusion’. A second validation was performed by analyzing the membrane fluidity of CHO cells incubated with arachidonic acid. An increase in membrane fluidity was observed both experimentally by using pyrene decanoic acid and by using LION/web, showing significant enrichment of terms associated with high membrane fluidity ('above average’, 'very high’ and 'high lateral diffusion’, and 'below average transition temperature’). The results demonstrate the functionality of LION/web, which is freely accessible in a platform-independent way.
**Corresponding Author:**	Bernd Helms<br><br>NETHERLANDS
**Corresponding Author Secondary Information:**	
**Corresponding Author's Institution:**	
**Corresponding Author's Secondary Institution:**	
**First Author:**	Martijn R. Molenaar
**First Author Secondary Information:**	
**Order of Authors:**	Martijn R. Molenaar<br>Aike Jeucken<br>Tsjerk A. Wassenaar<br>Chris H. A. van de Lest<br>Jos F. Brouwers<br>Bernd Helms
**Order of Authors Secondary Information:**	
**Response to Reviewers:**	Reviewer #1:<br>The authors have responded appropriately to my comments, adding statistical options and an example of the target mode. In the pdf, the figure quality appears to be low, but that may be related to the conversion to pdf. In particular, I can't read the text on Figure 2 at all.<br><br>We thank the reviewer for her support. Indeed, the low resolution is related to the pdf-conversion. There is a hyperlink on top of the figure pages to download the figures in

	the original resolution.<br><br>Reviewer #2:<br>This is a tremendously improved version of the manuscript. All critiques are adequately addressed, and the current revision is logical and detailed.<br><br>We appreciate the positive comments of the reviewer.<br><br>A couple of minor comments:<br>#1. The use of colon in the linear regression terms (Suppl. Material #8) is very confusing given the fact that these terms are actually something opposite, i.e., products of corresponding predictors. This should be explicitly stated and the colon changed to something more appropriate.<br><br>We agree that the use of the colon in Supplementary Data 8 can be somewhat misleading. Accordingly, we changed the symbols into asterisks, a more appropriate way to indicate multiplication. The explanation has been added to each sheet of Supplementary Data 8.<br><br>#2. For LION term describing fatty acids with 2 or more double bonds a conventional designation as PUFAs (polyunsaturated FA) should be mentioned; the same may be also applied to their monounsaturated and saturated counterparts.<br><br>We thank the reviewer for pointing this out. We have changed the respective term names into 'polyunsaturated fatty acid', 'monounsaturated fatty acid' and 'saturated fatty acid', updated the web-tool and occurrences of these terms in the figures.
**Additional Information:**	
**Question**	**Response**
Are you submitting this manuscript to a special series or article collection?	No
**Experimental design and statistics**<br><br>Full details of the experimental design and statistical methods used should be given in the Methods section, as detailed in our Minimum Standards Reporting Checklist. Information essential to interpreting the data presented should be made available in the figure legends.<br><br>Have you included all the information requested in your manuscript?	Yes
**Resources**<br><br>A description of all resources used, including antibodies, cell lines, animals and software tools, with enough information to allow them to be uniquely identified, should be included in the Methods section. Authors are strongly encouraged to cite Research Resource	Yes

Powered by Editorial Manager® and ProduXion Manager® from Aries Systems Corporation

Identifiers (RRIDs) for antibodies, model organisms and tools, where possible.<br><br>Have you included the information requested as detailed in our Minimum Standards Reporting Checklist?	
**Availability of data and materials**<br><br>All datasets and code on which the conclusions of the paper rely must be either included in your submission or deposited in publicly available repositories (where available and ethically appropriate), referencing such data using a unique identifier in the references and in the "Availability of Data and Materials" section of your manuscript.<br><br>Have you have met the above requirement as detailed in our Minimum Standards Reporting Checklist?	Yes

Manuscript w DOI

Click here to view linked References

Click here to access/download;Manuscript;20190409 LION Manuscript_after review final NN_MM_JBH W DOI.docx

LION/web: a web-based ontology enrichment tool for lipidomic data analysis

Martijn R. Molenaar[§], Aike Jeucken[§&], Tsjerk A. Wassenaar[‡], Chris H. A. van de Lest[§], Jos F. Brouwers[§], J. Bernd Helms*[§]

[§] Department of Biochemistry and Cell Biology, Faculty of Veterinary Medicine, Utrecht University, 3584 CM, Utrecht, The Netherlands

[‡] Groningen Biomolecular Sciences and Biotechnology Institute and Zernike Institute for Advanced Materials, University of Groningen, 9747 AG Groningen, The Netherlands

ORCIDs:

M.R.M: 0000-0001-5221-608X; T.A.W: 0000-0002-6345-0266; C.H.A.v..d.L.: 0000-0003-2143-2825; J.F.B: 0000-0003-2924-3279; J.B.H.: 0000-0001-7311-3821

[&] Current address: Membrane Enzymology, Groningen Biomolecular and Biotechnology Institute (GBB), University of Groningen, 9747AG Groningen, The Netherlands

* To whom correspondence should be addressed: J.B.H (J.B.Helms@uu.nl)

ABSTRACT

A major challenge for lipidomic analyses is the handling of the large amounts of data and the translation of results to interpret the involvement of lipids in biological systems. We built a new lipid ontology (LION) that associates over 50,000 lipid species to biophysical, chemical and cell biological features. By making use of enrichment algorithms, we used LION to develop a web-based interface (LION/web, www.lipidontology.com) that allows identification of lipid-associated terms in lipidomes. LION/web was validated by analyzing a lipidomic dataset derived from well-characterized sub-cellular fractions of RAW 264.7 macrophages. Comparison of isolated plasma membranes with the microsomal fraction showed a significant enrichment of relevant LION-terms including ‘plasma membrane’, ‘headgroup with negative charge, ‘glycerophosphoserines’, ‘above average bilayer thickness’, and ‘below average lateral diffusion’. A second validation was performed by analyzing the membrane fluidity of CHO cells incubated with arachidonic acid. An increase in membrane fluidity was observed both experimentally by using pyrene decanoic acid and by using LION/web, showing significant enrichment of terms associated with high membrane fluidity ('above average’, 'very high’ and 'high lateral diffusion’, and 'below average transition temperature’). The results demonstrate the functionality of LION/web, which is freely accessible in a platform-independent way.

KEYWORDS

lipidomics; lipids; membrane biology; lipid ontology; LION; LION-term enrichment analysis; membrane biology; web-tool; data analysis; LION/web

BACKGROUND

The comprehensive study of lipids, also termed lipidomics, is gaining momentum. Instrumentation is becoming increasingly more sensitive, precise and fast, and the use of lipidomics to address key questions in membrane biology has become widespread. As a result, datasets are rapidly increasing both in terms of size and complexity. Due to a lack of methods to perform global and in-depth data mining, lipidomic research tends to focus on individual lipid classes or lipid species. A common approach in other 'omics' disciplines to reduce complexity is the use of ontologies *e.g.*, Gene Ontology [1], Chemical Entities of Biological Interest ontology [2], combined with statistical tools to determine terms of interest. Although lipid structure is closely related to lipid function, it is currently impossible to associate properties of individual lipids with complex lipid mixtures of cellular lipidomes. Examples of biophysical properties that play an important role in membrane biology are numerous and include membrane thickness (*e.g.*, as driving force in the sub-cellular localization of proteins [3]), membrane fluidity (*e.g.*, regulating bacterial survival [4], membrane heterogeneity in cellular signaling [5]), intrinsic curvature (*e.g.*, of lipids as key player in lipid droplet biogenesis [6,7] or COPI coat disassembly [8]), and net charge (*e.g.*, of membranes as a determinant in lipid-protein interactions [9]). Here, we aim to provide a lipid ontology database and complementary enrichment analysis tool that (i) contains chemical and biophysical information of lipid species, (ii) is platform independent and compatible with routine mass spectrometry-based lipid analysis, (iii) can be used by researchers without computer programming skills, and (iv) is freely available to the scientific community.

FINDINGS

Basic structure of LION

We constructed an ontology database called LION (**File S1**) that links over 50,000 lipid species with four major branches: 'lipid classification' (the LIPIDMAPS classification hierarchy [10]), 'chemical and physical properties' (fatty acid length and unsaturation, headgroup charge, intrinsic curvature, membrane fluidity, bilayer thickness), 'function', and 'subcellular component' (predominant subcellular localisation). The resulting database contains more than 250,000 connections ('edges'), providing a detailed system for in-depth annotation of lipids. An example of all LION-terms associated with a single phosphatidylserine (PS) lipid species, PS(34:2), is depicted in **Figure S1**. We describe the construction of LION in more detail in the Methods section. All LION-terms, classification rules and references are described in **Data S1**, all lipids currently supported by LION are described in **Data S2**.

Addition of biophysical properties to LION

An important feature of LION is the association of lipid species with biophysical properties. We made use of experimental data (from five phospholipid classes and sphingomyelin) [11] and data (from five phospholipid classes) obtained by coarse-grain molecular dynamics simulation (CG-MD) [12], each providing distinct biophysical properties . These data were used to estimate the biophysical properties of all related lipids in the LION-database by multiple linear regression analysis.

The regression models were validated in two ways. First, we performed leave-one-out cross-validations (LOOCV) of all three models (**Fig. S2 A-C)**, showing satisfactory agreement between determined and predicted values. Second, we compared two properties closely associated with membrane fluidity: 'transition temperature' (from experimental datasets) and 'lateral diffusion' (from the CG-MD datasets) **(Fig. S2 D)**. As expected, lipids with low

transition temperatures were predicted to have high lateral diffusion values at a defined simulation temperature and vice versa.

Subsequently, all numerical datapoints for each biophysical property were categorized into five pre-defined groups (‘very low’, ‘low’, ‘average’, ‘high’, ‘very high’). We aimed to find group definitions with physiological relevance. Therefore, limits of each group were calculated on the basis of four mammalian lipidomics publications that served as a reference [13-16]. Using these group definitions, numerical values of all applicable lipid species present in LION were classified and connected to their respective LION-term (**Fig. S2 E**).

LION enrichment analysis and web-tool LION/web

Next, we used LION as a basis to build an ontology enrichment tool that facilitates reduction of lipidome complexities in an unbiased manner. To this end, we made use of an adapted version of ‘topGO’, an R-package designed for enrichment analysis of GO-terms [17]. Subsequently, we designed a web-tool with R-package Shiny (‘LION/web’, www.lipidontology.com) that offers an intuitive user-interface and supports two major workflows **(Fig. 1)**: enrichment analysis of a subset of lipids of interest (‘target-list mode’) and enrichment analysis performed on a complete and ranked list of lipids (‘ranking mode’, referred to as ‘SAFE’ and described in the context of genes [18]). A detailed step-by-step description of LION/web’s workflow can be found in **Note S1**.

Analogous to Gene Ontology enrichment approaches [1], which facilitate pre-selection of ontology sub-domains or subsets of GO-terms (‘GO-slims’), LION/web offers the option to limit analysis to specific LION-terms of interest. Furthermore, the web-tool allows removal of the most generic LION-term (the one with the highest hierarchy) if a related term contains the same subset of lipids. For example, the term ‘diacylglycerophosphocholines’ might be associated with the same lipids as ‘glycerophosphocholines’. With this option switched on, only the most specific term (‘diacylglycerophosphocholines’) is included in the results.

Performance of 'target-list mode' by LION/web

To test the functionality of LION/web, we made use of a previously published and well characterized dataset containing lipidomics data from several sub-cellular fractions of RAW 264.7 macrophages, with or without TLR-4 activation by Kdo_2-lipid A (KLA) [13] (see Methods for a direct link to the dataset). First, we re-normalized the dataset by expressing all lipid species as fraction of the total amount of lipid per sample. Subsequently, the data were visualized by constructing a heat map graph **(Fig. 2 A)**. Lipid species were grouped into 10 clusters by hierarchical clustering. Each lipid cluster was subsequently analyzed by LION/web, which was able to reformat and match the vast majority (>97%) of the submitted lipids in the dataset. In the 'target-list mode', LION/web assesses the enrichment of LION-terms in a subset of lipids, as compared to all lipids in the experiment. For every cluster, lipids **(Data S3)** were entered as target-list and compared with the background list. Enrichment analysis of all 10 clusters resulted in at least one significant LION-term **(Fig. 2 B)**. The heat map showed that lipids present in clusters 7 and 8 were abundant in the mitochondrial fractions **(Fig. 2 A).** In line with this observation, enrichment analyses of these clusters resulted in significant terms associated with this organelle (*e.g.,* 'diacylglycerophosphoetahnolamines', 'mitochondrion', 'diacylglycerophosphoglycerols', 'headgroup with negative charge'). Similar results were obtained for cluster 6 (terms related to the plasma membrane), and to lesser extent for cluster 9 (terms related to endoplasmic reticulum). Lipids in cluster 5 were more abundant in KLA-treated fractions and resulted in terms reported by LION/web that were associated with low membrane fluidity.

Performance of 'ranking mode' by LION/web

An alternative method to assess enrichment of LION-terms in LION/web is the 'ranking-mode'. In the 'ranking-mode', all individual lipid species of two conditions are compared and ranked based on a 'local' statistic. This local statistic is any numeric value that associates

individual (hence ‘local’) lipids with the provided conditions. LION/web supports three different local statistics: one-tailed Welch two sample t-tests *P*-values (comparison of 2 conditions); ^{2}log fold-change values (comparison of 2 conditions) and one-way ANOVA F-tests *P*-values (comparison of >2 conditions). Subsequently, the distributions of all associated LION-terms over the ranked list are compared to uniform distributions by using one-tailed Kolmogorov–Smirnov tests (‘global’ statistics, as full lipidomes are assessed). A LION-term is enriched when its associated lipids are higher ranked than expected by chance. To illustrate the ‘ranking mode’, we compared the isolated PM fraction (samples #19-21 from **Fig. 2 A)** with the ER fraction (samples #13-15 from **Fig. 2 A)** from non-stimulated macrophages using one-tailed Welch two sample t-tests *P*-values as local statistic. Subsequently, LION/web assessed all LION-terms for enrichment **(Fig. 2 C)**. In good agreement with current descriptions of the selected organelles [19,20], significant enriched LION-terms included terms associated with chemical descriptions (*e.g.*, ‘glycerophosphoserines’, ‘headgroup with negative charge’, ‘phosphosphingolipids’), biological features (‘plasma membrane’) and biophysical properties (*e.g.*, ‘above average bilayer thickness’, ‘below average lateral diffusion’, ‘very low lateral diffusion’, ‘very high bilayer thickness’, ’neutral intrinsic curvature’). LION/web also reported the significant enrichment of ‘very high transition temperature’, which is in line with the (very) low lateral diffusion terms (see also **Fig. S2 D**). The term ‘very low transition temperature’ was also reported to be significantly enriched. Inspection of the lipid species responsible for the LION-term ‘very low transition temperature’ revealed the presence of lipids that all contain polyunsaturated fatty acids (PUFAs) with at least four unsaturations. This may be a macrophage-specific phenomenon, related to their involvement in inflammation [21].

Enrichment performance of chemical and biophysical LION-terms

To further characterize the enrichment of chemical and biophysical properties by LION/web, we used two different experimental approaches. First, we investigated the enrichment of

chemical features that can be easily incorporated into lipids. To this end, CHO-k1 cells were incubated overnight in the presence of palmitic acid (PA), linoleic acid (LA) or arachidonic acid (AA) complexed to bovine serum albumin (BSA). Subsequently, lipids were analysed by LC-MS/MS and quantified. When available, we used MS/MS data to annotate lipids with their most abundant fatty acid composition. This level of annotation is important as it enables LION to link input lipids with terms associated with fatty acids **(Data S4** and **Fig. S3).** Next, the web-tool was set to use the 'ranking mode' and to limit analysis to LION-terms indicating the presence of fatty acids as lipid building blocks. LION/web reliably reported the significant enrichment of the respective fatty acid in the three different conditions **(Fig. 3 A** and **Data S5)**.

Second, to investigate the enrichment of biophysical LION-terms, we incubated CHO-k1 cells with arachidonic acid (AA). This procedure is known to increase membrane fluidity [22]. After incubation, the membrane fluidity properties of the samples were analyzed both experimentally and by LION/web. Membrane fluidity was experimentally assessed using pyrene decanoic acid (PDA) **(Fig. 3 B)**. This fluorescent probe can exist as monomer or excimer, resulting in a shift of its emission spectrum. The ratio of excimer over monomer fluorescence is proportional to the degree of membrane fluidity [23]. As expected, the ratio of excimer/monomer forms of PDA revealed a significant increase in membrane fluidity of lysates in the presence of AA **(Fig. 3 C)**. For parallel LION/web analysis of membrane fluidity properties, lipids were extracted from the same samples and analysed by LC-MS/MS **(Data S6** and **Fig. S4)**. LION contains two sets of terms associated with membrane fluidity: 'transition temperature' and 'lateral diffusion'. Accordingly, LION/web was set to limit enrichment analyses to these sets, after which the lipidomic data were analyzed ('ranking mode'). In line with the experimentally measured increase in membrane fluidity, terms associated with high membrane fluidity ('above average', 'very high' and 'high lateral diffusion', and 'below average transition temperature') were significantly enriched in cells that had been treated with AA **(Fig. 3 D** and **Data S7)**.

DISCUSSION

Despite the quick grow of lipidomics and the rise of many tools to process raw data into lipid compositions [24], no automated pipeline to reduce complexity in lipidomic datasets using prior knowledge was yet available. Such a tool facilitates the generation of hypotheses, which is an important aim in many omics experiments. Here, we have presented a new ontology called LION and have used this ontology to build a web-based online LION-term enrichment tool suited to fulfill this need. In a single analysis, trends in complex lipidomic datasets can now be assessed in a standardized way. The web-tool assures that the pipeline is accessible to users that are not familiar with programming languages.

Just like enrichment analysis approaches in other omics fields, LION-term enrichment analysis comes with specific strengths and limitations. The quality and coverage of the underlying ontology is of great importance. For LION, we aimed to support most commonly found lipid species in mammalian systems. In our examples, >85% of the input lipids could be matched to the ontology. Due to the great diversity of lipidomes in different organisms, this coverage could be be lower in user-provided datasets from non-mammalian systems. We hope to support LION's coverage of plant, bacterial and yeast lipidomes better in the future. LION/web offers users several feedback options to keep track of missing annotations and to act specifically upon users' needs.

It is important to note that the enrichment of biophysical properties such as membrane fluidity, membrane thickness and curvature cannot replace functional assays. More factors than lipids alone – protein composition, temperature - are playing important roles. Moreover, the effect of cholesterol is complex and depends on the interaction with other lipids. Therefore, the biophysical effects of cholesterol are not included. Also, the relative amounts of lipids in the described enrichment analysis methods are not taken into account: low abundant lipids contribute equally to enrichment as their high abundant counterparts.

This limitation can be circumvented by defining local statistics that takes abundancies into

account. This type of statistic will become more urgent when lipidomic analyses shifts from mostly semi-quantitative to quantitative analyses in the future.

In summary, LION/web reveals changes in lipid patterns that allow researchers to study the complexity of lipidomes in a biological context. With future expansions of the LION database and of LION/web (also upon request of the scientific community), LION/web will be increasingly successful to bridge the gap between lipidomics and cell biology.

METHODS

Creation of lipid ontology (LION)

We built an ontology database that connects lipid species to the following four major branches: 'lipid classification', 'function', 'cellular component' and 'physical or chemical properties'. For readability, a term is included at the top of each branch to indicates the nature of a LION-branch. These 'category' terms are distinguished from other LION-terms with an ID containing the prefix 'CAT'.

The classification system is based on the LIPIDMAPS classification [10]. LIPIDMAPS does not support lipid species with summed fatty acid. However, this extra layer is useful as it enables mapping or when exact fatty acid compositions of measured lipids are not known. This concept is also used in the Swiss Lipids system [25]. Downstream, individual lipid species belonging to classes described in **Data S1** were constructed as combinations of the following fatty acids: C12:0; C14:0; C14:1; C16:0; C16:1; C18:0; C18:1; C18:2; C18:3; C20:0; C20:1; C20:2; C20:3; C20:4; C20:5; C22:0; C22:1; C22:2; C22:3; C22:4; C22:5; C22:6; C24:0; C24:1; C24:2; C24:3; C24:4; C24:5; C24:6; C26:0; C26:1; C26:2; C26:3; C26:4; C26:5; C26:6 and C26:7. For sphingolipids, sphingosine (d18:1) and sphinganine (d18:0) were used as possible backbones. In the current version, LION does not distinguish between *sn*-positions. Fatty acids were ordered by chain length (low to high) and number of unsaturations (low to high). Altogether, LION contains circa 50,000 lipid species.

The branch ‘function’ comprises three subcategories: ‘membrane component’ (associated with lipids that are primary regarded as structural component of lipid bilayers), ‘lipid-mediated signaling’ (lipids that have been implicated in signaling) and ‘lipid-storage’ (lipids that are associated with storage, primarily in lipid droplets). In the category ‘cellular component’, lipid classes that are enriched in particular cellular organelles are linked to their corresponding organelle terms [7,19,20]. The branch ‘physical or chemical properties’ comprises a number of subcategories. First, a number of chemical descriptions (‘contains fatty acid’, ‘fatty acid unsaturation’, ‘fatty acid length’ and ‘type by bond’) was inferred from the species names. Second, data about ‘intrinsic curvature’ [7,26] were categorized into either negative, neutral or positive curvature. As data on species-level are limited, curvature was assumed to be predominantly headgroup-dependent and fatty acid composition was neglected. The third subcategory, ‘charge headgroup’, was divided into three groups based on structural data: ‘negative’, ’positive/zwitter-ion’ and ‘neutral’ [25]. This last term comprises also lipids lacking a headgroup. The fourth subcategory in ‘physical or chemical properties’ is 'chain-melting transition temperature'. This property is derived from a number of sources, comprehensively reviewed by Marsh [11]. This dataset covers a range of lipid classes in both glycerophospholipids (PC, PE, PG, PA, PS) and sphingolipids (SM). We made use of multiple linear regression analysis with lipid class, fatty acid length and unsaturation as predictors to facilitate data extrapolation to previously unreported lipid species. The obtained model (coefficients are available via **Data S8)** was validated by leave-one-out cross-validation (LOOCV). Briefly, one datapoint from the dataset was taken out, after which the model was rebuilt with the remaining points as training set. Subsequently, the selected datapoint was used as validation sample. This procedure was repeated for all the datapoints (**Fig. S2 C)**. Ontologies contain categorical data and are not compatible with numeric values. Therefore, we classified chain-melting transition temperature values into five distinct categorical data groups: 'very low', 'low', 'average', 'high' or 'very high' chain-melting transition temperature. To define the limits of these intrinsic subjective groups, we used four previously reported

datasets to serve as reference lipidomes [13–16]. From all reported lipids, the transition temperature was predicted by the model. The obtained transition temperature distribution was used to define the groups: the lowest 20% (first quintile) was classified as ‘very low’, the second 20% (second quintile) as ‘low’, etc. Subsequently, these limits were used to categorize all lipid species present in LION. Lipids with transition temperature values lower than the lowest limit were defined as ‘very low’, whereas values higher than the highest limit were defined as ‘very high’. A flow-chart of this procedure is depicted in **Fig. S2 E**.

In addition to these experimental data sets, we also used data [12] that was obtained by coarse grain molecular dynamics simulation (MARTINI force-field [27]) and which includes membrane properties ‘bilayer thickness’ and ‘lateral diffusion’. The dataset contains lipids from five common classes of glycerophospholipids (PC, PS, PG, PA, PE), but lacks sphingolipids and sterols. By definition, coarse-grained lipids represent a range of structures. To be able to use the dataset in the ontology system, names of coarse-grained lipids were translated into their representing counterparts. Subsequently, lipid properties were extrapolated to the entire database by multiple linear regression analysis models (with lipid class, fatty acid length and unsaturation as predictors, coefficients are available via **Data S8**) and validated by LOOCV (**Fig. S2 A-B)**. We followed the same procedure as used for transition temperatures; extrapolated results for both properties were categorized into representative classes: 'very low', 'low', 'average', 'high' or 'very high', based on values, predicted by our models, of the reference datasets [13–16].

The initial structure of LION was built with OBOEdit v.2.3.1 [28] and formatted as OBO-file. Subsequently, custom R-scripts connected specific terms with more general terms based on the described datasets. The entire ontology can be found as **File S1.**

Implementation of enrichment analysis tool

To use LION with existing ontology enrichment tools, we used an adapted and generalized version of Bioconductor R-package ‘topGO’ [17]. This version, called ‘topOnto’, allows users to include ontologies other than those provided with the package. TopOnto’s attached Perl-

script was used to convert the ontology file from OBO- to SQLite-format. Apart from this extra feature, the 'topOnto' package provides the same functionality as the original version. To perform the enrichment analysis, two statistical approaches are used. In the 'target-list mode', one-tailed Fisher-exact statistics are used to test enrichment. To this end, 2x2 contingency tables are constructed for every LION-term, containing the number of lipids associated and not associated with the given term for both the target-list and the background set, and analyzed. In the 'ranking mode', one-tailed Kolmogorov-Smirnov tests are used as 'global' statistics to assess enrichment of LION-terms over a ranked (by 'local' statistics) list of lipids. For every LION-term, the cumulative distribution of associated lipids over the ranked list is compared with the uniform distribution. Enrichment is defined as over-representation of highly ranked lipids associated with the term. To rank input lipids, LION/web offers three different 'local' statistics: *P* values from one-tailed Welch t-tests (2 condition comparison), ^{2}log fold-change values (2 condition comparison) and *P* values from one-way ANOVA F-tests (>2 conditions comparison). Ranking direction (from high to low, or vice versa) is automatically updated after local statistic selection, but can be set manually. In addition, users can use custom local statistics. In both modes, topGO's classic algorithm is selected [17]. After LION enrichment analysis, raw *P* values are corrected for multiple testing (Benjamini-Hochberg). The R-scripts were used to build the user-friendly web-based tool LION/web **(Note S1)** with R-package 'shiny'. The application has been made available on the shinyapps.io server as a free online tool, accessible through http://www.lipidontology.com/.

Cell culture and preparation of fatty acid-albumin complexes

CHO-k1 cells were cultured in Ham's F-12 medium (Thermo Fisher Scientific, Waltham, MA, USU) supplemented with 7.5% FBS (Thermo Fisher Scientific, Waltham, MA, USU), 100 units/ml penicillin and 100 μg/ml streptomycin (Thermo Fisher Scientific, Waltham, MA, USU). Cells were grown in a humidified incubator at 37°C containing 5% CO_2 and passaged

twice a week. Stocks of 10 mM arachidonic acid, linoleic acid, oleic acid, or palmitic acid (all obtained from Sigma, St. Louis, MO, USA) were complexed to 2 mM fatty-acid free BSA (Sigma, St. Louis, MO, USA), filter-sterilized and stored at −20 °C. Control incubations without fatty acid contained equivalent amounts of fatty-acid free BSA. All experimental incubations were performed in plastic 6-well culture dishes (Corning, Tewksbury, MA, USA).

Measuring membrane fluidity

After overnight incubation in the absence or presence of fatty-acids (using fatty acid-free BSA or fatty acids coupled to BSA, respectively), cells were washed and scraped in PBS. Cells were subsequently homogenized on ice with 26-gauge needles (BD Bioscience, San Jose, CA, USA). Homogenates (equivalent to 40,000 cells) were mixed 1:1 with the manufacturer's supplied dilution buffer (Membrane fluidity kit, Abcam, Cambridge, UK) in the absence (background) or presence of 5 μM pyrenedecanoic acid (PDA) and transferred into a 96-well plate (black plastic with glass bottom, Greiner Bio-One, Frickenhausen, Germany). After 30 minutes of incubation at 37°C, fluorescence spectra (excitation at 360 nm, emission between 375-500 nm, 37°C) were measured with a temperature-controlled fluorescence microplate reader (CLARIOstar, BMG Labtech, Offenburg, Germany). Data were processed in R by expressing monomer (370-390 nm) and excimer (470-490 nm) as ratios of mean fluorescence after subtraction of background fluorescence (samples with cells but without PDA). Results were expressed as means. Differences were analyzed by two-tailed Welch's t-tests.

Lipidomics by LC-MS/MS

After incubation, lipids were extracted as described before [29]. Lipid extracts were dried under nitrogen and dissolved in 100 μL chloroform/methanol (1:1) and injected (10 μL) on a hydrophilic interaction liquid chromatography (HILIC) column (2.6 μm HILIC 100 Å, 50 x 4.6 mm, Phenomenex, Torrance, CA). Lipid classes were separated by gradient elution on an Infinity II 1290 UPLC (Agilent, Santa Clara, CA, USA). At a constant flow rate of 1 ml/min,

ACN/acetone (9:1, v/v) was used as solvent A. Solvent B consisted of a mixture of ACN/H2O (7:3, v/v) with 10mM ammonium formate. Both solvents contained 0.1% formic acid. The gradient was as follows (time in min, %B): (0, 0), (1, 50), (3, 50), (3.1, 100), (4, 100). Samples were injected without re-equilibration of the column. The column effluent was connected to a heated electrospray ionization (hESI) source of an Orbitrap Fusion mass spectrometer (Thermo Scientific, Waltham, MA) operated at -3600V in the negative ionization mode. Temperatures for the vaporizer and ion transfer tube were 275°C and 380°C, respectively. Full scan MS1 measurements in the mass range from 450 to 1150 amu were collected in the orbitrap at a resolution of 120.000. Parallelized data-dependent MS2 experiments were done with HCD fragmentation set at 30V, using the dual stage linear ion trap to generate up to 30 spectra per second.

Lipidomics data analysis

Acquired raw datafiles were converted to mzXML-files by msConvert (part of ProteoWizard v3.0.913) [30] and processed with R-package 'xcms' v2.99.3 [31]. After deisotoping, annotation of lipids was performed by matching measured MS-1 m/z values with theoretical m/z values. Lipids with the same or similar m/z values - *e.g.*, BMP(38:4) and PG(38:4) - could by distinguished by differences in retention time **(Fig. S3 and S4)**. Lipid annotation containing individual fatty acids (extra column 'most abundant isomer annotation' in **Data S4**) as used in **Fig. 2 A** and **Fig. S3** was accomplished by examining MS-2 spectra. When MS-2 spectra were available for a given MS-1 peak, the most abundant fatty acid combination was used to annotate the lipid. The resulting experimental datasets, as well as the public RAW 264.7 macrophage dataset [13], were normalized by expressing all lipids as ratios of the sum of all intensities per sample. MetaboAnalyst 3.0 [32] was used to replace missing values (of the RAW 264.7 dataset) by half of the minimum positive value in the original data, and to perform Principal Component Analysis (with Pareto scaling).

Heat map, hierarchical cluster analysis and LION-enrichment analyses

The heat map of the RAW 264.7 dataset was constructed after calculating z-scores for all lipids (all lipids were scaled to a mean of zero and a standard deviation of 1) using R-package ‘pheatmap v1.0.10’. Lipids were grouped by hierarchical clustering. The dendrogram of the lipids on the y-axis of the heatmap used Euclidean distance as the similarity measure and was performed with complete linkage. The number of clusters was set to 10. Enrichment analysis of each of the 10 clusters was performed using the ‘target-list mode’ with default settings. Enrichment analyses used in **Fig. 2 C** and **Fig. 3 A and D** were performed using the ‘ranking mode’, with one-tailed Welch two sample t-tests P-values as local statistics. The analysis for **Fig. 2 C** was performed with default settings, whereas LION-terms to be considered were limited to all child-terms of 'contains fatty acid' (CAT:0000100) for **Fig. 3 A** and all child-terms of 'chain-melting transition temperature' (CAT:0001734) and 'lateral diffusion' (CAT:0080950) for **Fig. 3 D**.

Software and R-packages

All R-scripts were run with RStudio v1.0.153 (R v3.4.4) with the following packages: ‘shiny v1.1.1’, ‘visNetwork v2.0.1’, ‘data.table v1.10.4-2’, ‘GMD v0.3.3’, ‘igraph v1.0.1’, ‘reshape2 v1.4.2’, ‘ggplot2 v2.2.1’, ‘ggthemes v3.4.0’, ‘shinyTree v0.2.2’, ‘shinyWidgets v0.4.1’, ‘shinythemes v1.1.1’, ‘RSQLite v2.1.1’, ‘topOnto v0.99.0’, ‘pheatmap v1.0.10’ and ‘xcms v2.99.3’ [31]. Perl-scripts provided with the topOnto package were run with Perl v5.26.0. All figures were built in R and processed in Cytoscape v3.5.1 or Inkscape v0.92.2.

Availability of source code and requirements

The source code of the web-tool is available via github.

Project name: LION-web

Project home page: https://github.com/martijnmolenaar/LION-web/

Operating system(s): platform independent

Programming language: R

License: GNU General Public License v3.0

RRID: SCR_017018

Availability of supporting data

The LION database (OBO-format) and raw lipidomics data are available as Supplementary Data. The public RAW 264.7 macrophages dataset [13] is available on the journal's website [33]. R-package 'topOnto' is available at [34], the associated R-package containing the LION database in topOnto-friendly format at [35]. Snapshots of our code and other supporting data are available in the *GigaScience* repository, GigaDB [36].

ABBREVIATIONS

LION: lipid ontology; CG-MD: coarse-grain molecular dynamics simulation; LOOCV: leave-one-out cross-validations; GO: gene ontology; PUFA: polyunsaturated fatty acid; PA: palmitic acid; LA: linoleic acid; AA: arachidonic acid; BSA: bovine serum albumin; LC-MS/MS: liquid chromatography – tandem mass spectrometry; PDA: pyrene decanoic acid; CSV: comma separated values

COMPETING FINANCIAL INTERESTS

The authors declare no competing financial interests.

FUNDING

This research was supported by grant number BB/2012-10047 of the biobased ecologically balanced sustainable industrial chemistry (BE-BASIC) project to JBH and JFB.

AUTHOR CONTRIBUTIONS

M.R.M. and J.B.H. conceived the project. M.R.M. developed LION, LION/web and performed the experiments. A.J. tested and suggested improvements for LION/web.

C.H.A.v.d.L. and T.A.W. contributed to the regression models and statistical concepts. C.H.A.v.d.L. and J.F.B. contributed to the lipidomics data processing and analysis. M.R.M. and J.B.H. wrote the manuscript.

ACKNOWLEDGEMENTS

We thank Xin He, PhD, for providing and supporting the topONTO R-package. We thank Jeroen W.A. Jansen for the excellent technical assistance with the lipidomics experiments.

ADDITIONAL MATERIAL

Figure S1. LION-terms associated with PS(34:2).

Figure S2. Model validations of biophysical properties in LION.

Figure S3. Lipidomics of CHO-k1 cells incubated with free fatty acids.

Figure S4. Lipidomics of CHO-k1 cells incubated with arachidonic acid (AA).

Supplementary Data 1. XLSX-file containing all LION-terms excluding lipids with classification rules and sources.

Supplementary Data 2. CSV-file containing all lipids present in LION.

Supplementary Data 3. CSV-file with lipid clusters.

Supplementary Data 4. CSV-file with lipidomics dataset supporting Figure 2D.

Supplementary Data 5. CSV-file with LION/web output values supporting Figure 2D.

Supplementary Data 6. CSV-file with lipidomics dataset supporting Figure 2A.

Supplementary Data 7. CSV-file with LION/web output values supporting Figure 2A.

Supplementary Data 8. XLSX-file containing the coefficients of the biophysical models.

Supplementary Data 9. CSV-file with test-set for lipid names conversion.

Supplementary File 1. LION-database in OBO-format.

REFERENCES

[1] M. Ashburner, C. A. Ball, J. A. Blake, D. Botstein, H. Butler, J. M. Cherry, A. P. Davis, K. Dolinski, S. S. Dwight, J. T. Eppig, M. A. Harris, D. P. Hill, L. Issel-Tarver, A. Kasarskis, S. Lewis, J. C. Matese, J. E. Richardson, M. Ringwald, G. M. Rubin, and G. Sherlock, “Gene ontology: tool for the unification of biology. The Gene Ontology Consortium.,” *Nat. Genet.*, vol. 25, no. 1, pp. 25–9, May 2000.

[2] K. Degtyarenko, P. de Matos, M. Ennis, J. Hastings, M. Zbinden, A. McNaught, R. Alcántara, M. Darsow, M. Guedj, and M. Ashburner, “ChEBI: a database and ontology for chemical entities of biological interest.,” *Nucleic Acids Res.*, vol. 36, no. Database issue, pp. D344-50, Jan. 2008.

[3] H. J. Sharpe, T. J. Stevens, and S. Munro, “A comprehensive comparison of transmembrane domains reveals organelle-specific properties.,” *Cell*, vol. 142, no. 1, pp. 158–69, Jul. 2010.

[4] M. E. Inda, M. Vandenbranden, A. Fernández, D. de Mendoza, J.-M. Ruysschaert, and L. E. Cybulski, “A lipid-mediated conformational switch modulates the thermosensing activity of DesK.,” *Proc. Natl. Acad. Sci. U. S. A.*, vol. 111, no. 9, pp. 3579–84, Mar. 2014.

[5] E. Sezgin, I. Levental, S. Mayor, and C. Eggeling, “The mystery of membrane organization: composition, regulation and roles of lipid rafts,” *Nat. Rev. Mol. Cell Biol.*, vol. 18, no. 6, pp. 361–374, Mar. 2017.

[6] K. Ben M’barek, D. Ajjaji, A. Chorlay, S. Vanni, L. Forêt, and A. R. Thiam, “ER Membrane Phospholipids and Surface Tension Control Cellular Lipid Droplet Formation.,” *Dev. Cell*, vol. 41, no. 6, p. 591–604.e7, Jun. 2017.

[7] A. R. Thiam, R. V Farese, and T. C. Walther, “The biophysics and cell biology of lipid droplets.,” *Nat. Rev. Mol. Cell Biol.*, vol. 14, no. 12, pp. 775–86, Dec. 2013.

[8] J. Bigay, P. Gounon, S. Robineau, and B. Antonny, “Lipid packing sensed by ArfGAP1

couples COPI coat disassembly to membrane bilayer curvature.," *Nature*, vol. 426, no. 6966, pp. 563–6, Dec. 2003.

[9] G. Enkavi, H. Mikkolainen, B. Güngör, E. Ikonen, and I. Vattulainen, "Concerted regulation of npc2 binding to endosomal/lysosomal membranes by bis(monoacylglycero)phosphate and sphingomyelin," *PLOS Comput. Biol.*, vol. 13, no. 10, p. e1005831, Oct. 2017.

[10] E. Fahy, S. Subramaniam, R. C. Murphy, M. Nishijima, C. R. H. Raetz, T. Shimizu, F. Spener, G. van Meer, M. J. O. Wakelam, and E. A. Dennis, "Update of the LIPID MAPS comprehensive classification system for lipids.," *J. Lipid Res.*, vol. 50 Suppl, no. Supplement, pp. S9-14, Apr. 2009.

[11] D. Marsh, "Structural and thermodynamic determinants of chain-melting transition temperatures for phospholipid and glycolipids membranes.," *Biochim. Biophys. Acta*, vol. 1798, no. 1, pp. 40–51, Jan. 2010.

[12] T. A. Wassenaar, H. I. Ingólfsson, R. A. Böckmann, D. P. Tieleman, and S. J. Marrink, "Computational lipidomics with insane: A versatile tool for generating custom membranes for molecular simulations," *J. Chem. Theory Comput.*, vol. 11, no. 5, pp. 2144–2155, May 2015.

[13] A. Y. Andreyev, E. Fahy, Z. Guan, S. Kelly, X. Li, J. G. McDonald, S. Milne, D. Myers, H. Park, A. Ryan, B. M. Thompson, E. Wang, Y. Zhao, H. A. Brown, A. H. Merrill, C. R. H. Raetz, D. W. Russell, S. Subramaniam, and E. A. Dennis, "Subcellular organelle lipidomics in TLR-4-activated macrophages.," *J. Lipid Res.*, vol. 51, no. 9, pp. 2785–97, Sep. 2010.

[14] R. A. Haraszti, M.-C. Didiot, E. Sapp, J. Leszyk, S. A. Shaffer, H. E. Rockwell, F. Gao, N. R. Narain, M. DiFiglia, M. A. Kiebish, N. Aronin, and A. Khvorova, "High-resolution proteomic and lipidomic analysis of exosomes and microvesicles from different cell sources," *J. Extracell. Vesicles*, vol. 5, no. 1, p. 32570, Jan. 2016.

[15] M. S. Köberlin, B. Snijder, L. X. Heinz, C. L. Baumann, A. Fauster, G. I. Vladimer, A. C. Gavin, and G. Superti-Furga, “A Conserved Circular Network of Coregulated Lipids Modulates Innate Immune Responses,” *Cell*, vol. 162, no. 1, pp. 170–183, Jul. 2015.

[16] L. Lin, Y. Ding, Y. Wang, Z. Wang, X. Yin, G. Yan, L. Zhang, P. Yang, and H. Shen, “Functional lipidomics: Palmitic acid impairs hepatocellular carcinoma development by modulating membrane fluidity and glucose metabolism.,” *Hepatology*, vol. 66, no. 2, pp. 432–448, Aug. 2017.

[17] A. Alexa and J. Rahnenfuhrer, “Gene set enrichment analysis with topGO,” *Bioconductor*, 2017.

[18] W. T. Barry, A. B. Nobel, and F. A. Wright, “Significance analysis of functional categories in gene expression studies: a structured permutation approach.,” *Bioinformatics*, vol. 21, no. 9, pp. 1943–9, May 2005.

[19] J. C. M. Holthuis and A. K. Menon, “Lipid landscapes and pipelines in membrane homeostasis.,” *Nature*, vol. 510, no. 7503, pp. 48–57, Jun. 2014.

[20] G. van Meer, D. R. Voelker, and G. W. Feigenson, “Membrane lipids: where they are and how they behave.,” *Nat. Rev. Mol. Cell Biol.*, vol. 9, no. 2, pp. 112–24, Feb. 2008.

[21] P. C. Calder, “Marine omega-3 fatty acids and inflammatory processes: Effects, mechanisms and clinical relevance.,” *Biochim. Biophys. Acta*, vol. 1851, no. 4, pp. 469–84, Apr. 2015.

[22] X. Yang, W. Sheng, G. Y. Sun, and J. C. M. Lee, “Effects of fatty acid unsaturation numbers on membrane fluidity and α-secretase-dependent amyloid precursor protein processing.,” *Neurochem. Int.*, vol. 58, no. 3, pp. 321–9, Feb. 2011.

[23] J. Eisinger and S. F. Scarlata, “The lateral fluidity of erythrocyte membranes temperature and pressure dependence,” *Biophys. Chem.*, vol. 28, no. 3, pp. 273–281, Dec. 1987.

[24] D. Schwudke, A. Shevchenko, N. Hoffmann, and R. Ahrends, “Lipidomics informatics

for life-science," *J. Biotechnol.*, vol. 261, pp. 131–136, Nov. 2017.

[25] L. Aimo, R. Liechti, N. Hyka-Nouspikel, A. Niknejad, A. Gleizes, L. Götz, D. Kuznetsov, F. P. A. David, F. G. van der Goot, H. Riezman, L. Bougueleret, I. Xenarios, and A. Bridge, "The SwissLipids knowledgebase for lipid biology.," *Bioinformatics*, vol. 31, no. 17, pp. 2860–6, Sep. 2015.

[26] A. I. P. M. de Kroon, P. J. Rijken, and C. H. De Smet, "Checks and balances in membrane phospholipid class and acyl chain homeostasis, the yeast perspective.," *Prog. Lipid Res.*, vol. 52, no. 4, pp. 374–94, Oct. 2013.

[27] S. J. Marrink, A. H. de Vries, and A. E. Mark, "Coarse Grained Model for Semiquantitative Lipid Simulations," *J. Phys. Chem. B*, vol. 108, no. 2, pp. 750–760, 2004.

[28] T. Wächter and M. Schroeder, "Semi-automated ontology generation within OBO-Edit.," *Bioinformatics*, vol. 26, no. 12, pp. i88-96, Jun. 2010.

[29] E. G. Bligh and W. J. Dyer, "A rapid method of total lipid extraction and purification," *Can. J. Biochem. Physiol.*, vol. 37, no. 8, pp. 911–917, Aug. 1959.

[30] R. Adusumilli and P. Mallick, "Data Conversion with ProteoWizard msConvert.," *Methods Mol. Biol.*, vol. 1550, pp. 339–368, 2017.

[31] C. A. Smith, E. J. Want, G. O'Maille, R. Abagyan, and G. Siuzdak, "XCMS: processing mass spectrometry data for metabolite profiling using nonlinear peak alignment, matching, and identification.," *Anal. Chem.*, vol. 78, no. 3, pp. 779–87, Feb. 2006.

[32] J. Xia, I. V Sinelnikov, B. Han, and D. S. Wishart, "MetaboAnalyst 3.0--making metabolomics more meaningful.," *Nucleic Acids Res.*, vol. 43, no. W1, pp. W251-7, Jul. 2015.

[33] A. Y. Andreyev, E. Fahy, Z. Guan, S. Kelly, X. Li, J. G. McDonald, S. Milne, D. Myers, H. Park, A. Ryan, B. M. Thompson, E. Wang, Y. Zhao, H. A. Brown, A. H. Merrill, C. R. H. Raetz, D. W. Russell, S. Subramaniam, and E. A. Dennis, "Subcellular organelle

lipidomics in TLR-4-activated macrophages.," *J. Lipid Res.*, vol. 51, no. 9, pp. 2785–97, Sep. 2010. Supplemental data 1. http://www.jlr.org/content/suppl/2010/06/23/jlr.M008748.DC1/jlr.M008748-1.xls. Accessed 9 Jan 2018.

[34] topOnto R-package. https://github.com/hxin/topOnto . Accessed 26 Nov 2017.

[35] LION-database R-package. https://github.com/martijnmolenaar/topOnto.LION2.db. Accessed 10 April 2019.

[36] Molenaar MR; Jeucken A; Wassenaar TA; van de Lest CHA; Brouwers JF; Helms JB: Supporting data for "LION/web: a web-based ontology enrichment tool for lipidomic data analysis" *GigaScience* Database. 2019. http://dx.doi.org/10.5524/100599

FIGURE LEGENDS

Figure 1. Enrichment analysis approaches supported by LION/web. A lipidomics dataset containing lipid identifiers and abundances derived from two or more conditions (❶) can be processed by LION/web in two ways. In the 'target-list mode' (left, ❷), a subset of lipids (*e.g.*, derived from thresholding or clustering) is compared to the total set of lipids. After standardization of lipid nomenclature (❸), applicable LION-terms are associated and assessed for enrichment in the subset by Fisher's exact statistics. In the 'ranking mode', input lipids are ranked by numeric values ('local' statistics) (❷). After ranking, lipid nomenclature is standardized (❸). Applicable LION-terms are subsequently associated to the dataset and distributions are compared to a uniform distribution by 'global' statistics (here, Kolmogorov–Smirnov tests). Calculated *P* values of LION-terms from both modes are corrected for multiple testing (Benjamini-Hochberg).

Figure 2. LION-term enrichment analysis of RAW 264.7 macrophages. (A) Heatmap of scaled lipid amounts (z-score < 0: blue, z-score > 0: red) of subcellular lipidomics data [13] with samples on the x-axis and individual lipid species on the y-axis. Lipids were clustered into 10 groups by hierarchical clustering. **(B)** Enrichment analyses of all lipid clusters in the 'target-list mode'. For each cluster, the first $n + 2$ significant LION-terms are shown. **(C)** Enrichment analysis of PM *vs*. ER fractions in the 'ranking mode'. The gray vertical lines indicate the cut-off value of significant enrichments ($q < 0.05$). Bar colors are scaled with the enrichment (-log q-values).

Figure 3. LION-term enrichment and membrane fluidity of CHO-k1 cells. CHO-k1 cells were incubated overnight with PA, LA or AA (100 μM) **(A)** or with AA (250 μM) **(B-D)**. All incubations were performed in triplicate. For control incubations, cells were incubated with fatty-acid free BSA. **(A,D)** After extraction and lipidomics profiling by LC-MS/MS, enrichment analyses of the conditions of interest versus control incubations were performed by LION/web of **(A)** LION-terms indicating the presence of selected fatty acids or **(D)** LION-terms indicating the degree of membrane fluidity. Dot sizes in the dot plots are scaled to the number of associated lipids; colors are scaled to the level of enrichment (-log *q*-values). **(B,C)** After incubation, fluorescence emission spectra of lysates containing pyrenedecanoic acid (PDA) were measured **(B)**. Fluorescence spectra examples of either control (black) or AA-stimulated lysates (red). Gray shades indicate monomer and excimer fluorescence filters. **(C)** Mean ratios (bar) and individual datapoints (dots) of excimer over monomer fluorescence (representative data of three independent experiments). Statistical significance was determined by Student's two-tailed t-test. **(A,C,D)** * P or $q < 0.05$, ** P or $q < 0.01$, *** P or $q < 0.001$.

Figure 1

Click here to access/download;Figure;Figure 1.png

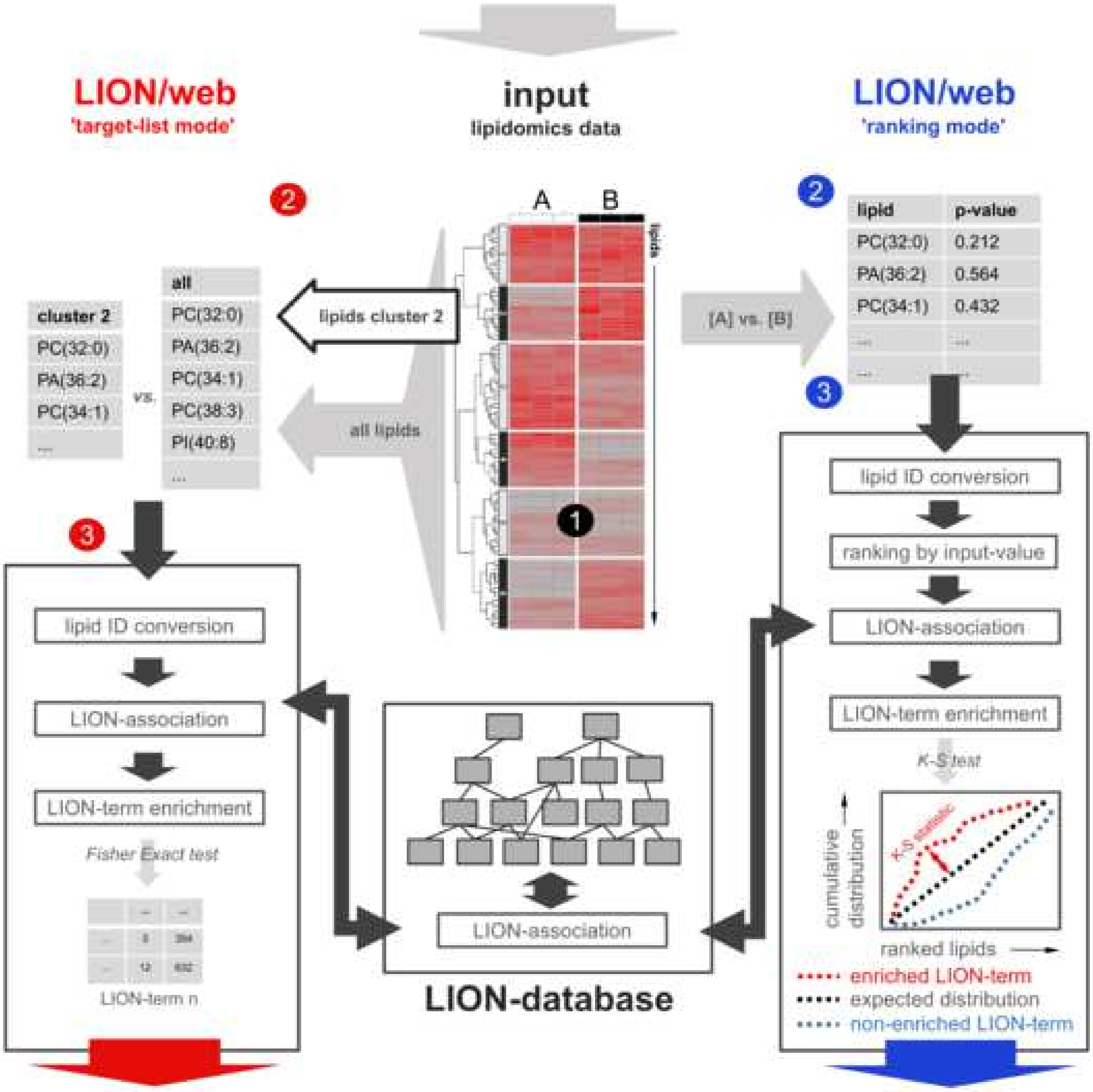

Figure 2 Click here to access/download;Figure;Figure 2.png

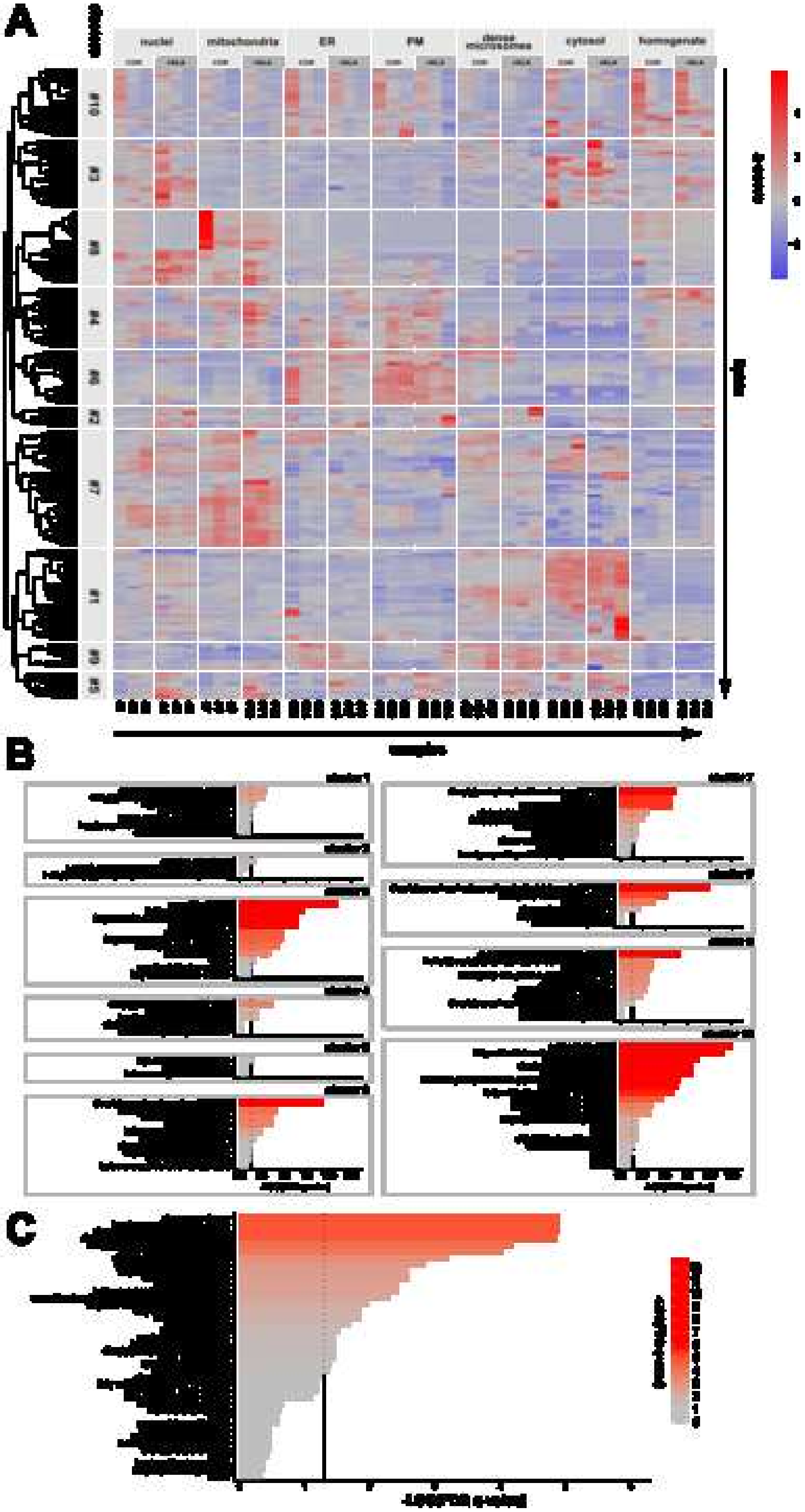

Figure 3

Click here to access/download;Figure;Figure 3.png

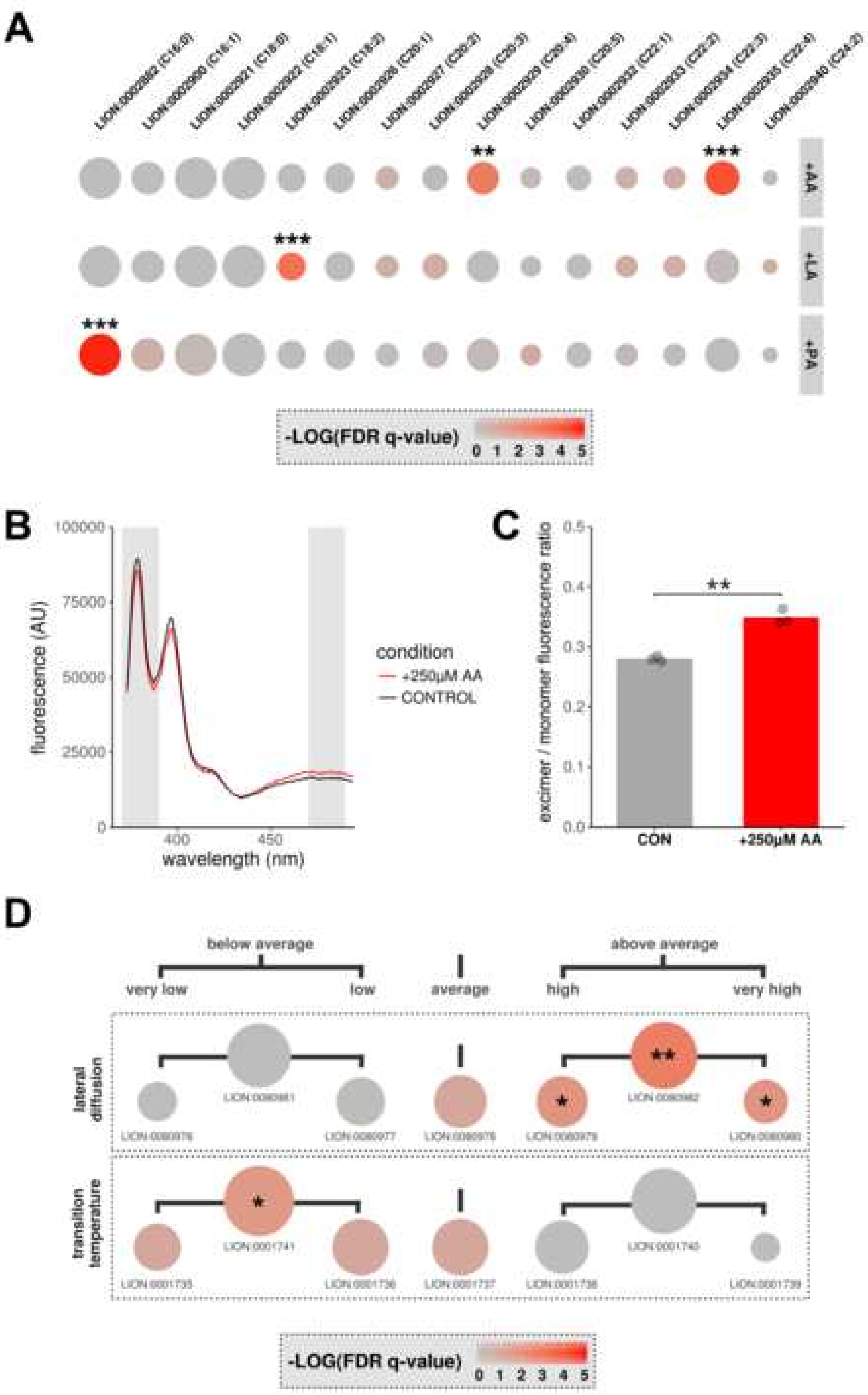

Supplementary text

Click here to access/download
Supplementary Material
20190226 LION Supplement_after review final.docx

Suppl. Fig. 1

Click here to access/download
Supplementary Material
Figure S1.png

Suppl. Fig. 2

Click here to access/download
Supplementary Material
Figure S2.png

Suppl. Fig. 3

Click here to access/download
Supplementary Material
Figure S3.png

Suppl. Fig. 4

Click here to access/download
Supplementary Material
Figure S4.png

Supplementary data 1

Click here to access/download
Supplementary Material
Supplemental Data 1.xlsx

Supplementary data 2

Click here to access/download

Supplementary Material

Supplemental Data 2.csv

Supplementary data 3

Click here to access/download
Supplementary Material
Supplemental Data 3.csv

Supplementary data 4

Click here to access/download
Supplementary Material
Supplemental Data 4.csv

Supplementary data 5

Click here to access/download
Supplementary Material
Supplemental Data 5.csv

Supplementary data 6

Click here to access/download
Supplementary Material
Supplemental Data 6.csv

Supplementary data 7

Click here to access/download
Supplementary Material
Supplemental Data 7.csv

Supplementary data 8

Click here to access/download
Supplementary Material
Supplemental Data 8.xlsx

Supplementary data 9

Click here to access/download
Supplementary Material
Supplemental Data 9.csv

Supplementary File 1

Click here to access/download

Supplementary Material

Supplemental File 1 LION.obo

Personal Cover

Click here to access/download;Personal Cover;Cover Letter Molenaar et al revised 2nd version.pdf

Universiteit Utrecht

GigaScience
The Editor
Nicole Nogoy, Ph.D

Faculty of Veterinary Medicine
Department of Biochemistry
and Cell Biology
PO Box 80176
3508 TD Utrecht
The Netherlands
Tel: +31-30-2535375
Fax: +31-30-2535492
Email: j.b.helms@uu.nl

April 12, 2019

Dear Nicole Nogoy,

Please find enclosed our second revised manuscript GIGA-D-18-00357R1 entitled "LION/web: a web-based ontology enrichment tool for lipidomic data analysis" by Martijn Molenaar et al.

Attached you will find a Point-by-Point answer to the comments to address the final revisions suggested by our reviewers. We thank the reviewers for their helpful comments that have helped to improve the quality of this manuscript.

We hope you find the manuscript now suitable for publication in GigaScience.
With kind regards

Bernd Helms

Prof. Dr. J.B. Helms
