# Supplementary material for: LION/web: a web-based ontology enrichment tool for lipidomic data analysis
Source: Gigascience. 2019 May 29;8(6):giz061. doi: 10.1093/gigascience/giz061 (PMC6541037; doi:10.1093/gigascience/giz061)
Supplement: giz061_Supplemental_Files [file giz061_supplemental_files.zip › Supplementary Information.pdf]

Supplementary Information

LION/web: a web-based ontology enrichment tool for lipidomic data analysis

Molenaar *et al.*

Supplementary Figures

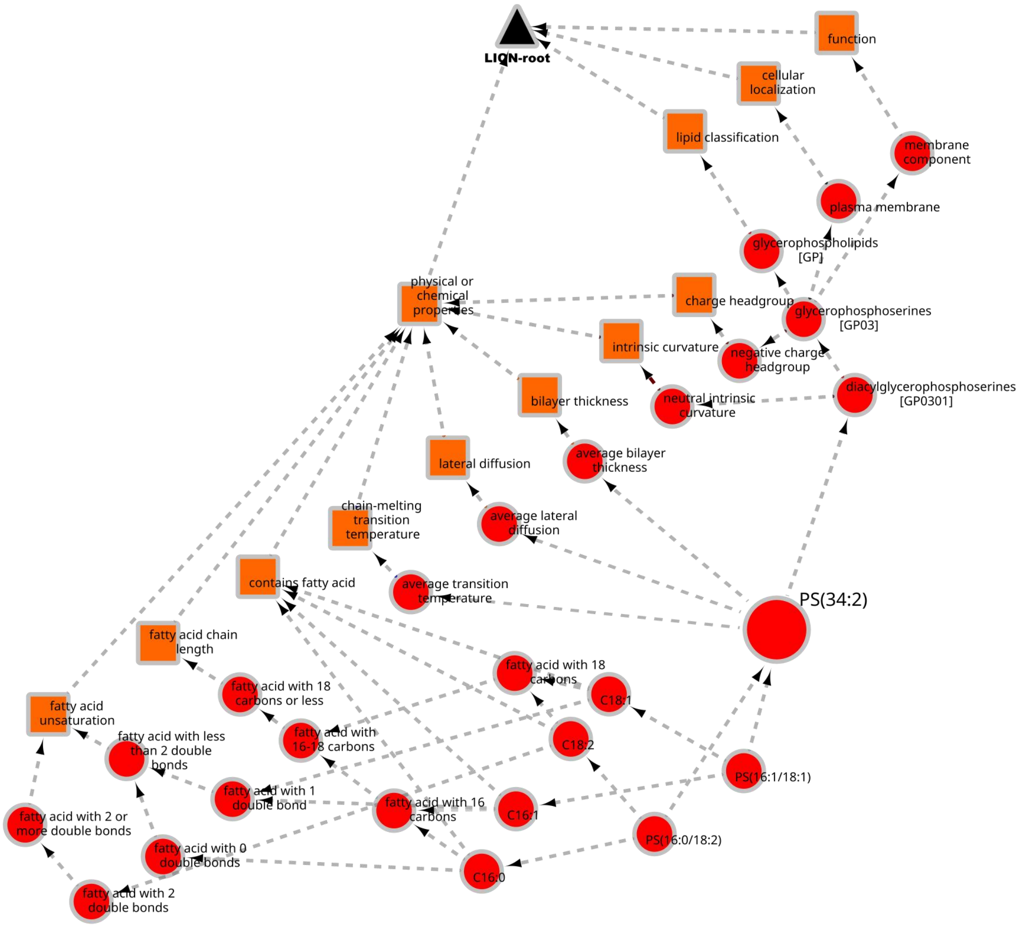

Figure S1. LION-terms associated with PS(34:2). All terms (shapes) with descriptions and edges (dashed lines) in LION associated with phosphatidylserine 34:2. LION's root is depicted as a black triangle. Terms that indicate the top of a category branch (terms with CAT-prefix) are shown as orange squares, categorical data are depicted as red circles.

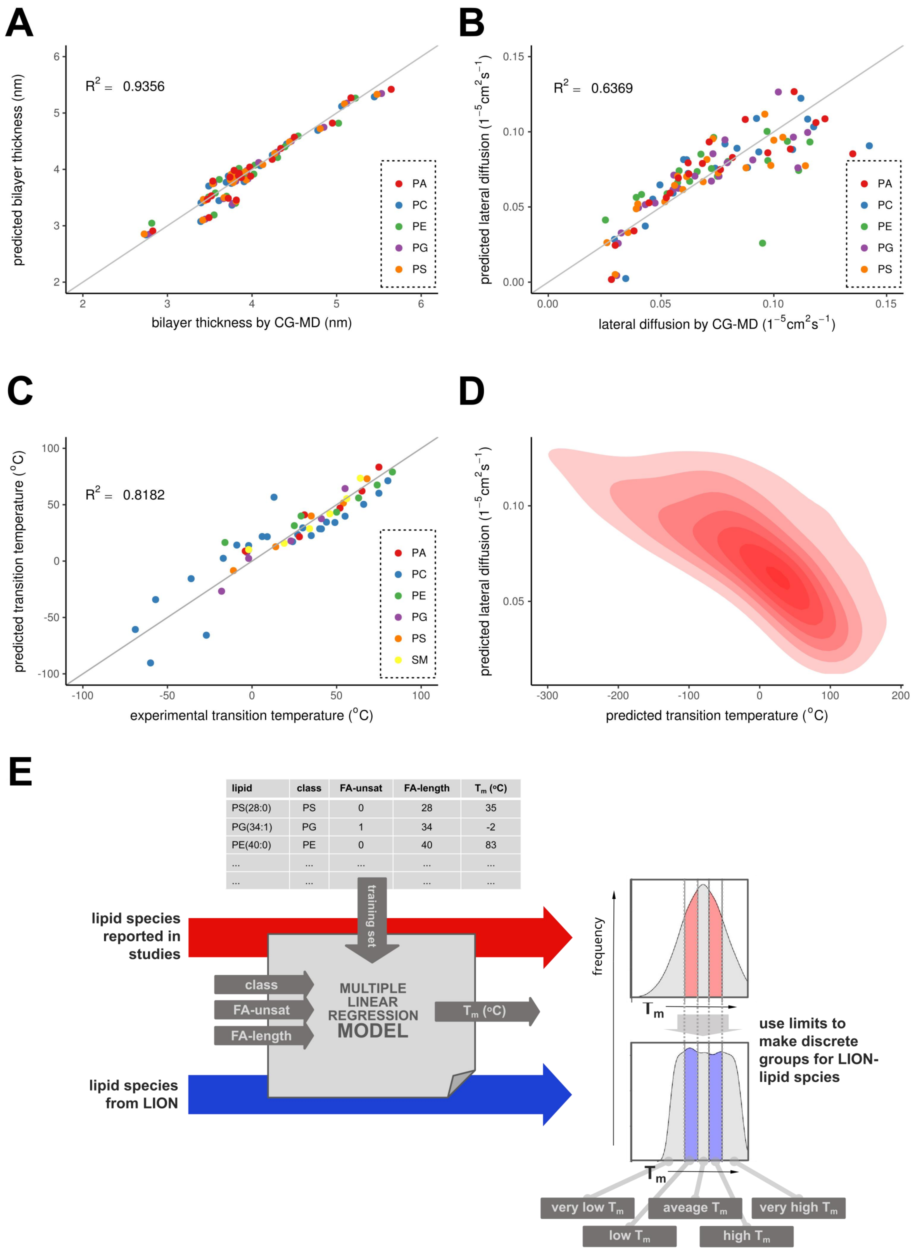

Figure S2. Model validations of biophysical properties in LION. Data about lipid classes, fatty acid lengths and level of unsaturation were used as predictors to build multiple linear regression analysis models with CG-MD **(A,B)** or experimentally derived **(C)** datasets. (**A-C)** Leave-one-out cross-validation plots of training set datapoints versus predicted values by the models of **(A)** bilayer thickness, **(B)** lateral diffusion and **(C)** transition temperature. Gray lines indicate theoretical relationships. Colors of the circles indicate lipid classes. **(D)** Density plot of the relation between predicted lateral diffusion and predicted transition temperature values of all applicable lipid species in LION. **(E)** Flow chart showing the categorization process of LION-terms associated with transition temperature. The same approach was used for bilayer thickness and lateral diffusion.

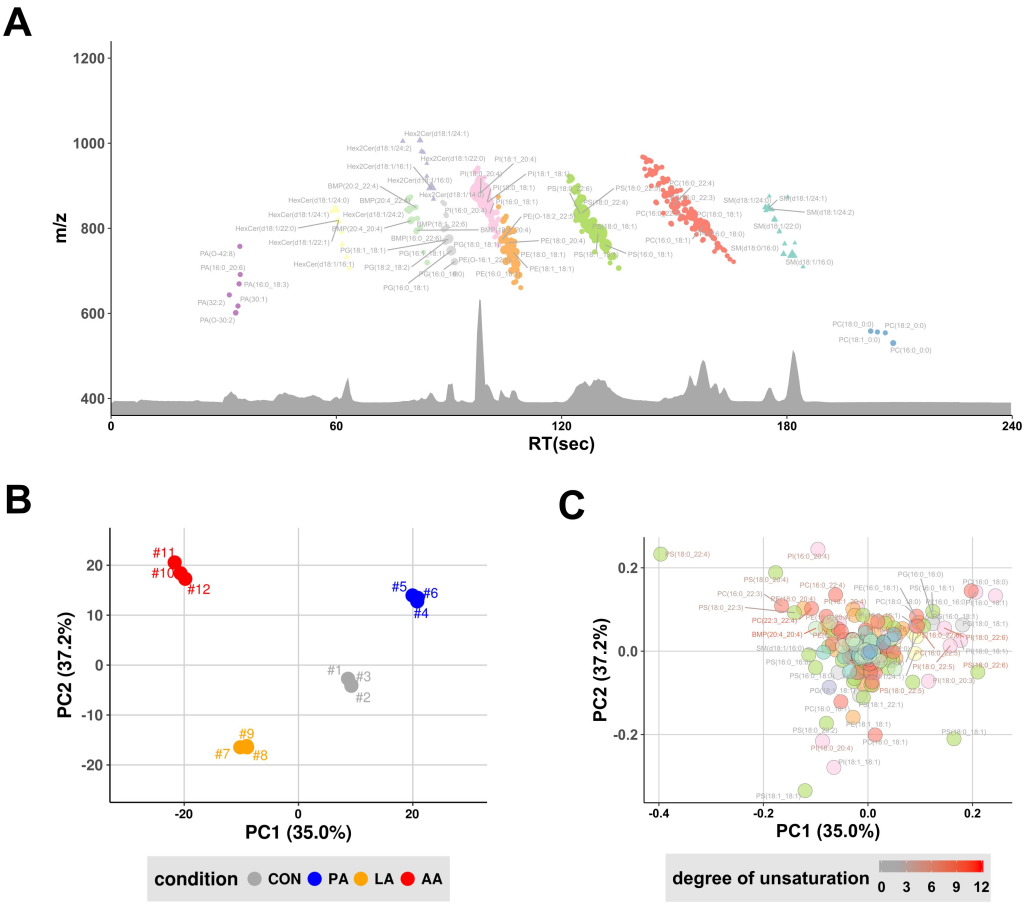

Figure S3. Lipidomics of CHO-k1 cells incubated with free fatty acids. CHO-k1 cells were cultured overnight with PA, LA or AA (100 µM, complexed to BSA) or control BSA. After extraction, lipids were measured by LC-MS/MS and processed as described in the Method section. **(A)** Scatterplot with retention times (RT) and m/z values of all annotated lipids. Each color represents a lipid class; sizes are scaled to mean lipid abundance. Circles represent phospholipids; triangles represent sphingolipids. The six most abundant species within every class are annotated in gray. The gray shade at the bottom of the plot represents the base peak ion chromatogram (BPC) of a control incubation. **(B,C)** Principal Component Analysis (PCA) with **(B)** score plot (indicating samples) and **(C)** loading plot (indicating lipid species). Colors of the labels are scaled to the degree of unsaturation (gray to red). Colors of the circles represent the same lipid classes as panel **A**.

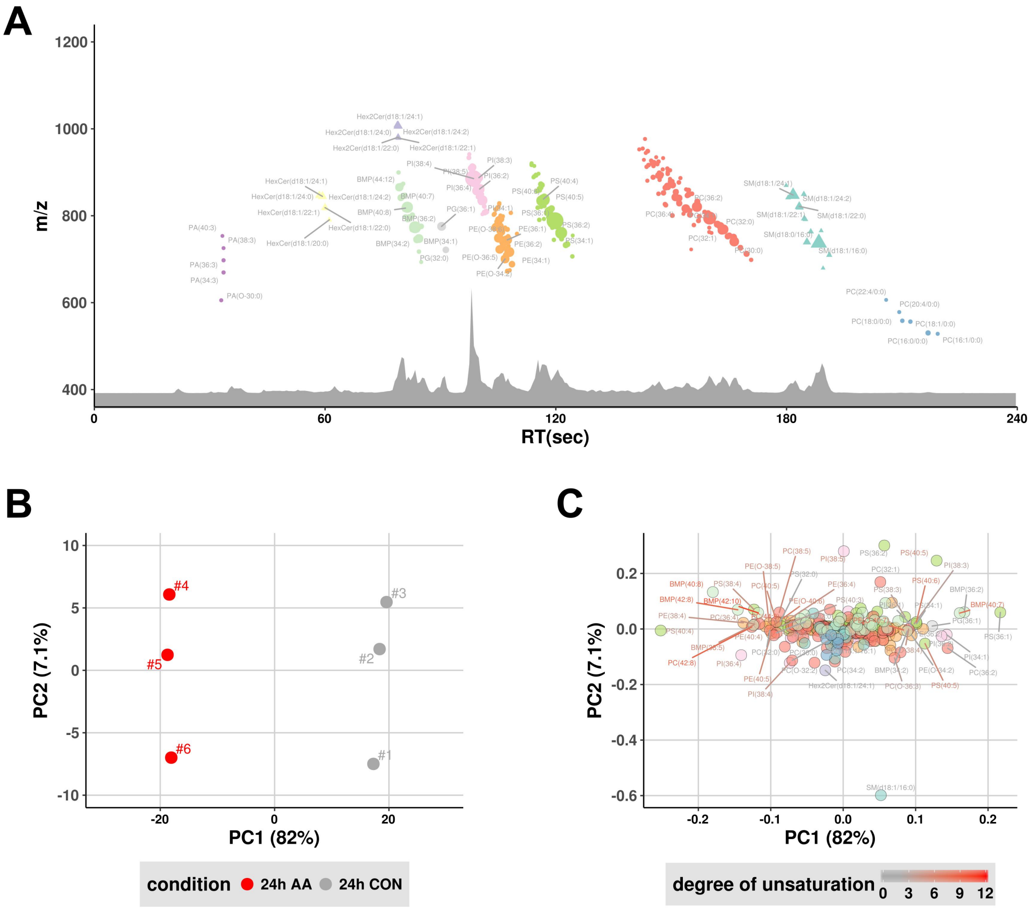

Figure S4. Lipidomics of CHO-k1 cells incubated with arachidonic acid (AA). CHO-k1 cells were cultured overnight with AA (250 µM) complexed to BSA or control BSA. After extraction, lipids were measured by LC-MS/MS and processed as described in the Method section. **(A)** Scatterplot with retention times (RT) and m/z values of all annotated lipids. Each color represents a lipid class, sizes are scaled to mean lipid abundance. Circles represent phospholipids, triangles sphingolipids. Names of the six most abundant species within every class are labeled in gray. The gray shade at the bottom of the plot represents the base peak ion chromatogram (BPC) of a control incubation. **(B,C)** Principal Component Analysis (PCA) with **(B)** score plot (indicating samples) and **(C)** loading plot (indicating lipid species). Colors of the labels are scaled to the degree of unsaturation (gray to red). Colors of the circles represent the same lipid classes as panel **A**.

Supplementary Notes

Note S1: 'Program Description For Users'

LION/web, accessible at www.lipidontology.com, offers an intuitive environment to perform LION-enrichment analysis. The obtained LION-terms provide unbiased information about differences between those sample groups.

STEP I: SETTINGS
Before analysis, LION/web can be customized by three optional settings. This panel can be found behind the blue toothed wheel (the first tab of the left panel).

Step I-a: 'Exclude parent terms with same associations as child'
In case LION-terms contain the exact same lipid associations and they have a child-parent relation, only the child be used in analysis when this option is set. This setting is switched on by default.

Step I-b: 'Automatically send unmatched lipids to LION-team'
To help to improve the coverage of LION, the application can be set to automatically send lipid identifiers that could not be matched to the webmasters. No user information or numeric data will be sent. This setting is not switched on by default.

Step I-c: 'Preselect LION-terms for analysis'
By default, LION/web will consider every LION-term. However, the application offers the option to limit analyses to terms selected by the user. To do so, switch on 'Preselect LION-terms for analysis' and select the LION-terms of interest.

STEP II: INPUT LIPIDOMICS DATA
LION/web supports two enrichment analysis modes: 'ranking-mode' and 'target-list mode'

Step II-a: input in 'ranking mode'
In this mode, enrichment analysis is performed by assessing LION-term distributions over a ranked list of lipids. Two statistics are used, a 'local' statistic to rank the lipids and a 'global' statistic to assess the distributions.

process input
The '(i) process input' tab on the left has a panel to upload a comma-separated values (csv-) file containing a lipidomics dataset that is formatted as follow; a column containing lipid identifiers (see **Note S2**), followed by columns containing normalized, numerical data. Columns should contain two rows of headers; the first row contains condition identifiers; the second row contains sample names. Example datasets are available on the bottom of the panel. After uploading, LION/web will recognize the sample groups, after which conditions of interest to compare can be selected by the users.

Selecting local statistics
Lipid identifiers can be ranked by three different 'local' statistics: (a) one-tailed Welch two sample t-tests *P*-values (2 conditions); (b) ^{2}log fold-change values (2 conditions) or (c) one-way ANOVA F-tests *P*-values (>2 conditions).

After clicking 'calculate local statistics', the selected values will be calculated for every lipid ('local' statistics) and be shown in a plot. The values will be used for analysis by clicking 'Use values as local statistics', after which the '(ii) analysis' tab will automatically open.

Analysis

In this tab, lipid identifiers with their corresponding local statistic is already pasted in the input field. Below the input field, LION/web automatically selects the correct 'ranking direction', depending on the selected local statistic.

Alternatively, users can skip previous steps and directly copy-paste lipids with custom local statistics in the input field when other statistics are desired. 'Ranking direction' should be set accordingly. Analysis is started by clicking on 'submit'.

Step II-b: input in 'target-list mode'

In this mode, a subset of lipids ('target-list') is compared to all lipids ('background') in the experiment. Enrichment analysis is performed by testing for over-presentation of LION-terms in the target list. The target-list mode offers two input field: the target-list (top field) and the background list (bottom field). Each field contains a list of lipid identifiers (see **Note S2**). Example datasets are available on the bottom of the panel. Analysis is started by clicking on 'submit'.

STEP III: INPUT MATCHING

After submission, LION/web matches the input to the lipid ontology database. If necessary, lipid names are reformatted to LION-compatible names (see **Note S2**). Subsequently, names or identifiers are compared with the association table that connects lipids to their most specific LION-terms. Lipids that cannot be linked to a LION-term are neglected in downstream analysis. To monitor this, LION/web returns a table that contains original input names together with either identified LION-term IDs and names, or with a label 'not found'. This table can be downloaded by clicking on 'download input table'. Only matched lipids will be used in the enrichment analysis.

STEP IV: ENRICHMENT ANALYSIS

The hierarchical nature of ontologies provides a framework that associates terms with all its downstream counterparts. Hence, every term can be viewed as a subset of associated lipids and, as a result, is suitable to be tested for enrichment. To enforce statistical power, only terms that can be associated with input lipids are used. In addition, category-terms (terms with CAT-prefix) are excluded. In the 'target-list mode', over-representation of terms in the subset is evaluated by one tailed Fisher tests. In the 'ranking mode', the input is ranked based on the local statistics. Subsequently, the distributions of all terms are compared to expected uniform distributions by chance and evaluated by one-tailed Kolmogorov-Smirnov tests ('global' statistics).

STEP V: RESULTS AND GRAPHICAL OUTPUT

The output of the enrichment analysis is presented as table, bar graph and network graph. The table ('LION enrichment table') contains IDs and descriptions of all terms used in the analysis. In addition, the table shows the raw P values obtained by either Fisher or Kolmogorov-Smirnov tests. To circumvent type I-errors, also corrected FDR (false discovery rate, Benjamini-Hochberg) q-values are provided. The column 'annotated' indicates the

number of lipids in the experiment that are associated with a given term. In the 'by target list' approach, the table includes two extra columns. The first ('significant') column contains information about the number of lipids that are associated with the given term and found in the target-list. The second ('expected') column provides the number of lipids that are expected by chance. The bottom of the page contains a button ('Download table') to download the table as CSV-file. Moreover, a detailed report containing all the experiment's LION-terms and lipid associations can be obtained with the button 'Download report'.

The enrichment analysis is also presented ('LION enrichment graph') as horizontal barplot. For readability, the terms are depicted on the y-axis and sorted based on enrichment. On the x-axis, the negative log scale of the FDR q-values is shown. The gray vertical line represents an FDR cut-off of 0.05. To further enhance intuitive interpretation of the plot, the colors of the bars are scaled to the FDR-values, starting with gray (for the non-significant terms) to bright red (for q-values lower than 1^{-10}).

Ontologies contain information about relations of its terms. As these relations are not shown in the enrichment table and graph, we also included a graph ('LION network view') that shows the enriched terms as a network. Here, all significant terms (with lower FDR q-values than 0.05) are selected. When less than four terms are significant, the four terms with the lowest FDR q-values are chosen. These terms, together with all terms that are present on the route to the ontology root (depicted as black triangle), are mapped as nodes in a hierarchical structure from left (root) to right (specific terms). In addition, terms are connected with gray arrows if they share a relation in LION. The nodes contain two types of information; (1) the size is scaled to the amount of lipids that are associated with the term and (2) the color is scaled to the raw *P* value of the enrichment (from gray to yellow to red). Category-terms are excluded from the enrichment analysis, but are included in the network. For readability, these terms are depicted as boxes. On the right side, the network contains control buttons to zoom in or out. On the other side, the network can be browsed by buttons for all four directions. For navigation, also the mouse cursor can be used.

Note S2: ‘Input identifiers conventions’

As lipid nomenclature is not fully standardized, we aimed to support several notation formats. The preferred input-format is based on LIPIDMAPS [10] and SwissLipids [25]. In short, lipid names start with a class prefix, followed by the fatty acid (FA) composition between parentheses. FA-composition must be formatted as the number of carbon-atoms and double bonds, separated by a colon. All FAs can be either summed or listed individually, separated by a slash (proved sn-position) or underscore (unknown sn-position). Currently, *sn*-position is not supported in LION. Therefore, slash and underscores are treated equal. Information about the position and nature (*cis* or *trans*) of double bonds are not supported and neglected. To enhance database-matching, LION/web reorders FAs automatically (ascending: first by level of unsaturation, second by fatty acid length). Serine-derivatives in sphingolipids (sphingosine or sphinganine), are represented as d18:1 or d18:0, respectively. Furthermore, lysolipids must be either formatted with a specific class prefix (e.g., LPC, LPE) or with its generic class prefix in combination with a missing FA (indicated with 0:0), *e.g.,* PC(18:1/0:0), PE(16:0/0:0). Shorthand notations without parentheses as proposed by Liebisch and colleagues [33] are also supported (note, however, that spaces in identifiers are prone to lead to unexpected behavior). In addition to human readable lipid identifiers, data can be introduced as LIPIDMAPS (*e.g.,* LMGP01010578, LMGP02010009) or Swiss Lipids identifiers (*e.g.,* SLM:000088148, SLM:000094452). Both knowledge databases offer search-engines on their website. Note that LIPIDMAPS does not support generalized lipid species, *e.g.,* PC(34:1).
We tested whether the conversion script of LION/web was able to reformat a number of non-standardized lipid identifiers from various sources into LION-friendly nomenclature. The results are depicted in **Data S9.**

Supplementary References

[33] Liebisch, G., Vizcaíno, J.A., Köfeler, H., Trötzmüller, M., Griffiths, W.J., Schmitz, G., Spener, F., and Wakelam, M.J.O. (2013). Shorthand notation for lipid structures derived from mass spectrometry. J. Lipid Res. *54*, 1523–30.
