# Supplementary material for: LION/web: a web-based ontology enrichment tool for lipidomic data analysis
Source: Gigascience. 2019 May 29;8(6):giz061. doi: 10.1093/gigascience/giz061 (PMC6541037; doi:10.1093/gigascience/giz061)
Supplement: giz061_Response_to_Reviewer_Comments_Original_Submission [file giz061_response_to_reviewer_comments_original_submission.pdf]

Author's Response To Reviewer Comments

Close

For a formatted version with answers to the comments in blue, please see uploaded document 'Response to reviewers'

Ruth Welti (Reviewer 1)

LION provides useful information helping users associate lipidomics data on membrane lipid species from mammalian systems with the chemical and physical properties of those systems. Overall this is an ambitious undertaking that is likely to provide insights on lipid properties, particularly to users that are not familiar with chemical or physical properties of membrane lipids. Overall, the tool seems useful and the paper is well-written, but a few points could be explained in more detail.

We appreciate the positive and constructive comments of the reviewer.

#1. It should be mentioned, and perhaps the authors could include an explanatory note at the site, noting that actual physical properties of membranes (such as fluidity) depend on factors in addition to the typically measured lipids, including sterols and protein type and content.

We incorporated a statement about this aspect at three different locations: i) in the web-tool (on the '?' sign, beneath the results output); ii) in a new F.A.Q. that is now available via the website; and iii) in the Discussion (line 200-204).

#2. It might be useful to point out specifically that the samples chosen to "calibrate" the lipid categorization are all from mammalian cells and thus the ability to accurately interpret lipidomics data from other types of systems is not clear. Perhaps this is because it is not clear to the reviewer precisely how the categorized lipids (page 4, lines 69-74) were used in the analysis. Since many mammalian tissues (e.g. brain, heart) have more extreme compositions, will this be a problem for analysis?

Indeed, we made use of mammalian lipidomics datasets as reference to define the groups of three biophysical properties. To emphasize this, we included a comment on LION's focus on mammalian lipidomes in the Discussion (line 193-197). This will, however, not compromise the results in specific examples as mentioned by the reviewer as the principle of LION/Web is based on sample comparison (Fig.1, sample A and B). A comparison between tissues with more extreme compositions (e.g. brain and liver) is likely to result in enriched terms related to very low Tm's or very high lateral diffusions, and in different lipid classes/species, results that reflect the respective lipidomes. Comparison between samples from the same tissue (e.g. wt brain vs. geneX-/- brain) will often yield more subtle differences, depending on the knockout. However, LION/Web will report any significant difference, e.g. if geneX affects lipid composition. The statistical power of the significance can be further increased by increasing the number of replicates (n).

#3. The ranking approach appears to be a pairwise comparison. I.e., even when multiple samples are present, comparison is to one (control) sample. This is analogous to a typical transcriptomic approach but, given that it's actually easier to collect lipidomic data than transcriptomic data on hundreds of samples/conditions, having to analyze the data pairwise might be a bit burdensome. Maybe you could discuss the choice of approach in the paper or clarify if the reviewer's understanding is incorrect.

We thank the reviewer for this comment. We have extended the web-tool with more options to calculate local statistics (values that are used to rank lipids). One of these options is the use of p-values derived from one-way ANOVA F-tests. This statistic analysis allows comparison of multiple conditions and can be used to rank the most fluctuating lipid species in datasets. Subsequent enrichment analysis will result in LION-terms summarizing these lipids.
A second option that we included to characterize lipidomic datasets with more than 2 conditions, is the use of hierarchical clustering in combination with the target-list mode. A new figure (Figure 2B) illustrates this approach using the same public dataset that we used in the initial version of the

manuscript. Enrichment analysis of the lipids in the clusters, in combination with a visual presentation of the clusters in relation to the conditions, further aids in characterization of the full dataset.

#4. An example showing the output from the target mode would be helpful to the reader.

We agree with the reviewer that the manuscript would benefit from an example of the target mode. As mentioned above (#3) we now include a new figure (Figure 2B) that shows a clustered heat map of the RAW 264.7 macrophages dataset. Each cluster is characterized by assessing LION-term enrichment of the lipids within each cluster, as compared to all the lipids in the experiment.

Aleksander Andreyev (Reviewer 2)

This technical note describes a LIpid-related ONtology database (LION) and accompanying enrichment analysis tool with potentially high value for lipidomics research. According to the authors they aim to "bridge<> the gap between lipidomics and cell biology" (p.7, l.138). A mere attempt at this herculean task is highly commendable. This entails, however, that the narration should be comprehensible for a non-expert user, presumably a cell biologist with little understanding of bioinformatics (which would be also in line with the GigaScience editorial guidelines). Unfortunately, the manuscript is plagued with multiple issues that make it very hard to understand the utility and intended use of the tools and nearly impossible to evaluate their validity. From the way manuscript is written, it feels as if it is intended more for bioinformatics audience which almost defeats the purpose. It is also somewhat disorganized with the logical flow being interrupted by off-hand remarks and description of one topic spread over different parts of the manuscript, sometimes repetitively. In a few cases, the text is burdened with statements of the obvious (e.g., "lipid structure is closely related to lipid function", "allows identification of lipid-associated terms in lipidomes"). There are multiple typos, grammar errors and misused words or terms that make a mere reading of the article a torture. One step to address this issues might be including subsections under the Findings section, another - careful reassessment of what material represents technical side and belongs to Methods and what should be in the Findings (my feeling is that a good portion of the LION description, currently under Methods, actually belongs to the Findings, right after the background information). The same goes to figure legends - I think currently they are overloaded with information that belongs in the Methods. The manuscript suffers from frequent use of vague statements. Instead of describing WHAT was done the authors simply state the means for doing it: "we used" this or that, "we made use of" this or that, such and such "was used", etc. Instead of explaining HOW something was done a bare statement "based on" is often made. References are missing (e.g., "as described in the literature", p.4, l.53, "was reported", p.5, l.99). The tally of connections between membrane biophysics and cell biology (p.3, l.35-43) looks random and lacking completeness. Besides, it seems somewhat misplaced. Authors use what appears to be in-house or jargon terms, such as "by target list", "by ranking" for the modes of the enrichment analysis, "local" statistics, etc. Use of such terms should be avoided. For such important terms as the modes of analysis the names should be related to their function and, ideally, self- explanatory (or, at least, thoroughly explained). All these issues pertaining to the quality of the narration should be addressed before the substance of the work can be properly evaluated.

We thank reviewer #2 for his thorough review report and would like to apologize for the typos and grammar errors in the manuscript that made 'reading of the article a torture'. As suggested, the Findings section is now subdivided into subsections with headings. We also include a separate Discussion section to avoid the 'interruption by off-hand remarks'.

Indeed, LION/web is intended to be useful for non-experts in bioinformatics. We recognize that some concepts used in the manuscript might be difficult to grasp with limited bioinformatics experience. Nevertheless, some basic understanding of data-analysis must be expected from users that obtained omics-data (which is obviously a prerequisite to use LION/web). In the updated version, we have provided more explanation and illustrate some of the concepts with examples in the following ways:
i) throughout the manuscript, we added additional information.
ii) we added a point-by-point frequently asked question (F.A.Q.) section in the web-tool, that can be accessed via the main menu of the website.

iii) we added ‘tooltips’ in the LION/Web application. Tooltips are pieces of information or instructions that appear when users hover the mouse cursor over an item - without clicking on it. This allows for specific instructions for specific steps.
Upon the reviewer’s request, we have considered several alternative names for the enrichment modes (ranking and target-list mode). However, we found the initial names to be the clearest, as it describes the difference between the modes the best. The use of a target-list (usually referred to as gene list, ID list, etc.) is also common practise in gene ontology enrichment procedures (DAVID, Panther, GOrilla). Users who have experience in this field will recognize the concept ‘target-list’. To improve the understanding of these terms/modes, we included more details about both modes in the Methods section. In addition, we added a new figure (Figure 2A+B) to illustrate the target-list mode.

With respect to the comment “The tally of connections between membrane biophysics and cell biology (p.3, l.35-43) looks random and lacking completeness” we note that the listing of biophysical properties related to membrane biology in the background section was not intended to be complete, but to provide a few intuitive examples. To clarify this, we put these examples in parentheses and ‘e.g.’.

Concerning missing references: Details about references per data source is now available via Supplemental Data 1. The statement ‘was reported’ (page-5/line-99 of initial manuscript) refers to LION-terms that were reported by the web-tool.

However, even in the present state the manuscript allows to point out the following weaknesses/areas for improvement:

#1. The LION should be completely verbally described (beyond the present reference to the .obo file). This should include a list of categorical ontology terms and rules of association between them. For the ones that are not obvious, a justification should be provided. As it stands now, the terms in question are hidden inside 1275-page long Excel file among about 50,000 terms representing individual lipids. Some of them relate to conventional structural elements of lipids, others are less obvious. For example, "fatty acid with 16-18 carbons" - is there any scientific meaning in this term? What is so special about this particular chain length? What exactly are the extra levels of classification between lipid classes and species? - they are mentioned but not described.

Upon the reviewer’s request, we describe LION in a better structured way by inclusion of two additional tables:
(1) Supplemental Data 1; describing all LION-terms (excluding lipid species), with detailed information about hierarchy, classification and references.
(2) Supplemental Data 2; describing all lipid species present in LION.

Concerning the scientific meaning of terms: one of the guiding principles of LION was to be able to construct defined subsets of lipids (‘terms’). LION/web then aids to report the most interesting subsets. Some of these subsets might be of interest, others might not. Scientific meaning should be evaluated by the scientist. For example, "fatty acid with 16-18 carbons" might indeed sound trivial at the first sight. Nevertheless, its enrichment could hint towards testable biological hypotheses.

#2. The enrichment tool is the crux of the article, the thing the authors are trying to "sell". However, there is no description of what it does and how it can be used. I flatter myself to be a qualified used but I could not make a head or tail of what the so called "by target list" mode does. If my "target list" includes unsaturated lipids I'll get enrichment in "double bonds", "below average transition temperature", etc. That much is obvious without running the tool. What else? What are the scenarios when I need to use it? Why do I need two lipidomic data sets for this? What does "derived from thresholding or clustering" mean?

We recognize that in the initial version of the manuscript, the use of the ‘target-list mode’ was not illustrated. We added an extra figure (figure 2) that demonstrates the use of both modes using the RAW 264.7 macrophages dataset (figure 2A+B for the target-list, figure 2C for the ranking mode). Figure 2C was a supplemental figure in the original manuscript.

The second mode, apart from the name (why "by ranking"? isn't this purely technical approach to facilitate stat analysis?), is less problematic. However, the option to limit analysis to a specific set of terms ("terms of interest") should be mentioned upfront. Then, the questions arise in what scenarios this would be advantageous? Would this create a bias in the analysis or not, both with regard to outcome and its stat significance?

We now describe the selection of specific sets at an earlier stage.

#3. The claim of the scope is overreaching. The "function" category, most interesting for cell biology researchers, appears to be extremely frugal, limited just to the crudest distinction between structural, signaling and storage functions. If this perception is correct, the LION would be of limited value for cell biology. The "chemical" properties appear to be a misnomer with chemical information limited purely to structural elements with no regard to reactivity, biochemical synthetic pathways, etc. I would say that, according to this Technical Note, the LION is the ontology linking lipidomics data to biophysical properties of corresponding membranes. The testing of the ontology was performed in a set of assays pertaining to membrane biophysics.

We found a single occurrence of 'cell biology' ('...web-tool bridges the gap between lipidomics and cell biology...') in the initial manuscript. This claim is now phrased with greater caution by '... future expansions of the LION database..., LION/web will be increasingly successful to bridge the gap between lipidomics and cell biology.' (line 216-218). However, we believe that besides 'function', also 'cellular component' and the biophysical properties are of interest for scientists studying cell biology. In addition, we will maintain the LION database and update it when new lipid data and functions of individual lipid species or classes become available (see also our reply to the comment of the expert editorial board member).

#4. It would be advantageous to sync terminology with other ontologies whenever possible, for example, use the GO term "cellular component" instead of "cellular localization", etc. "Lipid component" is a very dubious term for a structural lipid.

As suggested, we replaced the LION-term name "cellular localization" by "cellular component". "Lipid component" was a typo in the manuscript, and not the name of a term in LION. We apologise for this mistake.

#5. The biophysical properties of the vast majority of lipids were inferred from a limited set of literature data. It is therefore of utmost importance to thoroughly describe the approach used. What kind of data the sources provided? Where they for individual lipids or mixes, measured or calculated? How many entries? The equations for the multiple linear (sic!) regression analysis should be shown. The resulting coefficients could be of value by itself - why not publish them here?

We thank the reviewer for noticing the missing word 'linear'. We replaced multiple occurrences of 'multiple regression analysis' by 'multiple linear regression analysis'.
As mentioned earlier, we now include a supplemental table with data sources per LION-term. The raw numeric values (per lipid) of the biophysical properties derived from these sources were already provided together with the original manuscript in 'scripts' folder. It is our understanding that this folder is available to the reviewers (and to the public after publication).

We appreciate the suggestion to report the coefficients of the models. To this end, we now include an Excel spreadsheet containing the coefficients of the models, together with input cells to predict (numerical) values of the biophysical properties (Suppl. Data 8).

#6. The lipids appear to be divided into "quintiles" using a hard-to-describe (and almost lacking description in the manuscript) procedure based initially on a number of lipids in each group rather than the value of a biophysical parameter. What is the rationale for this? Does transition temperature of a lipid membrane care how many other membranes share the same value? I think the categorization should be based upon the magnitudes of biophysical properties alone. By the way, how many groups are actually there? The text says 5 but Fig. 2 shows 7… Also, Fig. 2 shows FDR q-values which are not mentioned in either legend or the main text.

We categorized 'transition temperature' into 5 groups: very low, low, etc. These descriptions are not defined and intrinsically subjective: whether a membrane has a low Tm depends on the context. To provide this context, we selected four lipidomics studies to serve as reference. Lipids from these reference lipidomes were ranked based in the predicted numeric values of the biophysical property. Then, the first 20% (first quintile) was defined as 'very low', the second 20% (second quintile) as 'low', etc. The limits of these quintiles were then used to classify all lipids present in LION. We believe that this approach defines the group limits with more physiological relevance. The alternative approach, based on

magnitudes of biophysical properties alone (as suggested by the reviewer) is more likely to yield a quintile 'average' for a group of non-physiological lipids.

The confusing of 5 groups vs. the 7 groups in figure 2 (now figure 3) is related to hierarchy. The groups 'very low ...' and 'low ...' are linked to a parental group called 'below average ...'. The same goes for 'high ...' and 'very high ...', they are linked to 'above average ...'. We updated the figure by adding a graphical representation of this hierarchy to the figure (new figure 3D). The hierarchy of LION-terms is also depicted in supplemental Data S1.

We now include a reference to 'q-values' in the figure legends.

#7. It is not absolutely clear from the manuscript but appears that the enrichment tool relies on the significance of the changes (p-value), as opposed to magnitude, to evaluate enrichment. Is this true? Is it possible that highly significant changes in low abundance lipids would dominate the outcome list without having much effect on the properties of membrane?

All enrichment analyses in the initial version of the manuscript used the ranking-mode with one-tailed t-test p-values to rank the lipids. Other statistical methods could be considered, but every choice has its pros and its cons. Magnitude (fold-change of condition B over condition A) has the undesirable property to overestimate effects when lipid concentrations are close to noise levels: it does not take sample variance into account. In contrast, p-values are more robust, but might be less intuitive to users without strong background in statistics. Using p-values, it is potentially possible that 'highly significant changes in low abundance lipids could dominate the outcome list'. However, most low abundant lipids usually display higher variance due to lower signal/noise levels. As a result, they usually do not generate extreme low p-values.

To provide more flexibility for users and to make the choice of a local statistic explicit, we now offer three local statistics (one-tailed t-test p-values, 2log fold-change, F-test p-values) in the updated version of the web-tool. The statistical method must be selected each time an analysis in the ranking mode is initiated.

#8. More detail should be provided on the statistics, for example, how the distribution curve was generated for K-S analysis, what were the input parameters for the Fisher exact test, etc.

We added more information in the Methods section.

#9. Methods for PDA assay and LC-MS should be brought to compliance with editorial guidelines to allow duplicate these studies. Missing are parameters such as cell number, concentration of the dye, shape of LC gradient, LC system used, MS/MS settings, to name a few. The full name of the Fusion mass spec should be provided because there are several different models. The text is not clear on the sequence of events: it sounds as if analyte ions fly from orbitrap to linear ion trap for detection - is this even possible?

We added details about the PDA in the manuscript.

The methods for LC-MS have now been described in greater detail to facilitate easy replication of experiments. Parallelization of MS1 and MS2 experiments has been clarified to avoid confusion. Current versions of the MS instrument are branded as 'Fusion Lumos' or 'Fusion IDX'. However, the original 'Orbitrap Fusion' mass spectrometer (serial number FSN10438) was branded under that name and this is the model used in our studies. Therefore, we cannot specify the type of instrument more accurately than we currently do.

#10. With regard to membrane fluidity data, although they show the desired differences they could be made much more convincing with appropriate controls subtracting intrinsic fluorescence of the cells.

The membrane fluidity data presented in the manuscript were subtracted from background fluorescence (blanks were samples with cells but without PDA dye). To make this clear, we updated the Methods section with this information.

#11. Annotating lipids with the "most abundant fatty acid composition" is misleading - if isobaric species are not resolved the overall composition (total carbons, total double bonds) should be shown as primary annotation (possibly followed by the most abundant isomer).

We now include the overall composition as primary annotation, together with a second column containing the most abundant isomer (Data S4). MS/MS analysis allows identification of the most abundant isomer (e.g. PC with a C18:1 and a C18:0 fatty acid) without assignment of the sn1/sn2 position of the respective fatty acids. It is important for experiments such as described in figure 3A to use identifiers containing individual fatty acids. LION-terms related to fatty acids cannot be associated to a dataset that lacks this information. To avoid confusion, we have renamed the lipid species from e.g. PC(18:1/18:0) to PC(18:1_18:0) to indicate the fatty acid composition of lipid species without sn1/sn2 assignment.

Expert editorial board comments on usability:

The following comments are thus from the perspective of a potential user.

Can the authors specify the source of the 50,000 lipid species included into the analyses? To my knowledge the lipidmaps database reports around 42,000 entries only.

We used the lipid classification system (hierarchy) in accordance with LIPIDMAPS. The individual lipid species in LION were constructed by combining lipid classes with abundant fatty acids. LIPIDMAPS is probably somewhat more stringent about the inclusion of lipid species in their database as they intend to provide additional information for individual species. We added a few lines (231-236) about the construction of individual lipid species in LION to the Methods section.

The number of lipid species linked to experimental or in silico data is more than two orders of magnitudes lower that the indicated number of 50,000 and mainly refers to membrane lipids. Are all of these 50,000 species associated with one or more than one feature? Can the authors comment how many of these 50,000 lipids are associated with features going beyond chemical properties? What kind of cell biological features were used and which of these features where linked to which lipid species? In order to understand and validate the assignments as more detailed description would be helpful.

Many lipids have a number of associations, whereas some lipids only have a few. As a consequence of the hierarchical structure of LION, lipids with only one association will not occur: lipids are (indirectly) associated with the neighbour's neighbour. To make this information more accessible for users, we improved the enrichment-report , which can be obtained by the button 'download report'. It now contains three files: a CSV-file with the enrichment information, a CSV-file containing all the LION-terms associated with the lipids in the dataset, and vice versa, a CSV-file containing all lipids of the dataset with associated LION-terms. With this information, users are better equipped to understand the underlying data structures and improve interpretation of obtained results.

Can the authors comment on why they integrated coarse-grain but not (in addition) atomistic MD data?

To our knowledge, there is no comprehensive lipid dataset available that has been obtained by atomistic molecular dynamics simulations. More importantly, the biophysical properties are categorized into distinct groups (very low, low, average, etc.). Given this categorization in groups, we suspect that the increased resolution of atomistic MD will be of no or very limited added value.

Can the authors specify which data of the two papers in particular was included into building the application?

We now provide a detailed supplemental table (Data S1) containing references per LION-term. Moreover, the source data and code are available via the script folder.

The fact that the application in its current form is restricted to glycerol-based lipids and fatty acids should be indicated in the abstract and in the discussion of the dataset.

We agree that the current LION database is not a complete end product. However, it is not true that LION only contains (associations to) glycerol-based lipids and fatty acids. The database includes many more: sphingolipids (sphingomyelins, ceramides, glycosphingolipids), cholesterol derivatives and retinoids. As comprehensive biophysical data about these classes is hardly available or too complex in the case of cholesterol, not all these classes are associated with biophysical properties. The biophysical properties obtained by MD are limited to glycerol-based lipids. The transition temperatures are also associated with sphingomyelins. Cellular component, intrinsic curvature, headgroup charge are

associated with many lipid classes. Limitations of LION/web are included in the Discussion section.

For this first version of LION the authors included only information from two publications. There is an increasing amount of data available going beyond this information. Can the authors comment on how they plan to allow for integration of additional information? Will users be able to do so in a 'customized' fashion?

We recognize the importance to involve users in the improvement of LION and LION/web. To this end, we added several features to the web-tool.
(i) We include an option (not selected by default for privacy reasons) that -when selected- informs us when lipids could not be matched to LION. This helps us to keep track of lipid identifiers that are often used, but not present in LION.
(ii) We include a contact form on the website to lower the threshold to contact us for questions, requests, suggestions or feedback.
Web-tool improvement will not stop after publication. Currently, we are working on features to build heat maps and principle component analyses within the web-tool. When new sources containing useful data become available, this will be added this to the database.

The power of application depends on the number of features associated with each lipid species. Can the authors comment on how they plan to advance the data base, e.g., by including the community? Will the application be hosted and if so, what is the perspective?

The full ontology, R-packages to perform enrichment analysis and R-code for the web-tool is publicly available. This is sufficient for experienced users to build customized versions of LION or the web-tool. We understand, however, that this will be challenging for inexperienced users. In the future, we plan to build a dedicated LION R-package with detailed instructions and guidelines to augment the ontology by individual users. An R-package provides more flexibility than a web-tool and the use of user-customized ontology versions will be easier to support.

The web-tool is currently hosted by Shinyapps.io. It will be hosted elsewhere in case this service discontinuous its operation. The domain name lipidontology.com is owned by the department and the web-tool LION/web will remain accessible via lipidontology.com.

Close
