# Supplementary material for: LION/web: a web-based ontology enrichment tool for lipidomic data analysis
Source: Gigascience. 2019 May 29;8(6):giz061. doi: 10.1093/gigascience/giz061 (PMC6541037; doi:10.1093/gigascience/giz061)
Supplement: giz061_Response_to_Reviewer_Comments_Revision_1 [file giz061_response_to_reviewer_comments_revision_1.pdf]

Author's Response To Reviewer Comments

Close

Reviewer #1:
The authors have responded appropriately to my comments, adding statistical options and an example of the target mode. In the pdf, the figure quality appears to be low, but that may be related to the conversion to pdf. In particular, I can't read the text on Figure 2 at all.

We thank the reviewer for her support. Indeed, the low resolution is related to the pdf-conversion. There is a hyperlink on top of the figure pages to download the figures in the original resolution.

Reviewer #2:
This is a tremendously improved version of the manuscript. All critiques are adequately addressed, and the current revision is logical and detailed.

We appreciate the positive comments of the reviewer.

A couple of minor comments:
#1. The use of colon in the linear regression terms (Suppl. Material #8) is very confusing given the fact that these terms are actually something opposite, i.e., products of corresponding predictors. This should be explicitly stated and the colon changed to something more appropriate.

We agree that the use of the colon in Supplementary Data 8 can be somewhat misleading. Accordingly, we changed the symbols into asterisks, a more appropriate way to indicate multiplication. The explanation has been added to each sheet of Supplementary Data 8.

#2. For LION term describing fatty acids with 2 or more double bonds a conventional designation as PUFAs (polyunsaturated FA) should be mentioned; the same may be also applied to their monounsaturated and saturated counterparts.

We thank the reviewer for pointing this out. We have changed the respective term names into 'polyunsaturated fatty acid', 'monounsaturated fatty acid' and 'saturated fatty acid', updated the web-tool and occurrences of these terms in the figures.

Close
