# Supplementary material for: LION/web: a web-based ontology enrichment tool for lipidomic data analysis
Source: Gigascience. 2019 May 29;8(6):giz061. doi: 10.1093/gigascience/giz061 (PMC6541037; doi:10.1093/gigascience/giz061)
Supplement: giz061_Reviewer_1_Report_Original_Submission -- Ruth Welti — 10/29/2018 Reviewed [file giz061_reviewer_1_report_original_submission.pdf]

Reviewer Report

Title: LION/web: a web-based ontology enrichment tool for lipidomic data analysis

Version: Original Submission **Date:** 10/29/2018

Reviewer name: Ruth Welti

Reviewer Comments to Author:

LION provides useful information helping users associate lipidomics data on membrane lipid species from mammalian systems with the chemical and physical properties of those systems. Overall this is an ambitious undertaking that is likely to provide insights on lipid properties, particularly to users that are not familiar with chemical or physical properties of membrane lipids.

Overall, the tool seems useful and the paper is well-written, but a few points could be explained in more detail.

1. It should be mentioned, and perhaps the authors could include an explanatory note at the site, noting that actual physical properties of membranes (such as fluidity) depend on factors in addition to the typically measured lipids, including sterols and protein type and content.
2. It might be useful to point out specifically that the samples chosen to "calibrate" the lipid categorization are all from mammalian cells and thus the ability to accurately interpret lipidomics data from other types of systems is not clear. Perhaps this is because it is not clear to the reviewer precisely how the categorized lipids (page 4, lines 69-74) were used in the analysis. Since many mammalian tissues (e.g. brain, heart) have more extreme compositions, will this be a problem for analysis?
3. The ranking approach appears to be a pairwise comparison. I.e., even when multiple samples are present, comparison is to one (control) sample. This is analogous to a typical transcriptomic approach but, given that it's actually easier to collect lipidomic data than transcriptomic data on hundreds of samples/conditions, having to analyze the data pairwise might be a bit burdensome. Maybe you could discuss the choice of approach in the paper or clarify if the reviewer's understanding is incorrect.
4. An example showing the output from the target mode would be helpful to the reader.

Level of Interest

Please indicate how interesting you found the manuscript: Choose an item.

Quality of Written English

Please indicate the quality of language in the manuscript: Choose an item.

Declaration of Competing Interests

Please complete a declaration of competing interests, considering the following questions:

- Have you in the past five years received reimbursements, fees, funding, or salary from an organisation that may in any way gain or lose financially from the publication of this manuscript, either now or in the future?
- Do you hold any stocks or shares in an organisation that may in any way gain or lose financially from the publication of this manuscript, either now or in the future?
- Do you hold or are you currently applying for any patents relating to the content of the manuscript?
- Have you received reimbursements, fees, funding, or salary from an organization that holds or has applied for patents relating to the content of the manuscript?
- Do you have any other financial competing interests?
- Do you have any non-financial competing interests in relation to this paper?

If you can answer no to all of the above, write 'I declare that I have no competing interests' below. If your reply is yes to any, please give details below.

I declare that I have no competing interests.

I agree to the open peer review policy of the journal. I understand that my name will be included on my report to the authors and, if the manuscript is accepted for publication, my named report including any attachments I upload will be posted on the website along with the authors' responses. I agree for my report to be made available under an Open Access Creative Commons CC-BY license (http://creativecommons.org/licenses/by/4.0/). I understand that any comments which I do not wish to be included in my named report can be included as confidential comments to the editors, which will not be published.

I agree to the open peer review policy of the journal

To further support our reviewers, we have joined with Publons, where you can gain additional credit to further highlight your hard work (see: https://publons.com/journal/530/gigascience). On publication of this paper, your review will be automatically added to Publons, you can then choose whether or not to claim your Publons credit. I understand this statement.

Yes Choose an item.
