# Supplementary material for: LION/web: a web-based ontology enrichment tool for lipidomic data analysis
Source: Gigascience. 2019 May 29;8(6):giz061. doi: 10.1093/gigascience/giz061 (PMC6541037; doi:10.1093/gigascience/giz061)
Supplement: giz061_Reviewer_2_Report_Original_Submission -- Aleksander Andreyev — 11/24/2018 Reviewed [file giz061_reviewer_2_report_original_submission.pdf]

Reviewer Report

Title: LION/web: a web-based ontology enrichment tool for lipidomic data analysis

Version: Original Submission **Date:** 11/24/2018

Reviewer name: Aleksander Andreyev

Reviewer Comments to Author:

This technical note describes a LIpid-related ONtology database (LION) and accompanying enrichment analysis tool with potentially high value for lipidomics research. According to the authors they aim to "bridge<> the gap between lipidomics and cell biology" (p.7, l.138). A mere attempt at this herculean task is highly commendable. This entails, however, that the narration should be comprehensible for a non-expert user, presumably a cell biologist with little understanding of bioinformatics (which would be also in line with the GigaScience editorial guidelines).
Unfortunately, the manuscript is plagued with multiple issues that make it very hard to understand the utility and intended use of the tools and nearly impossible to evaluate their validity. From the way manuscript is written, it feels as if it is intended more for bioinformatics audience which almost defeats the purpose.
It is also somewhat disorganized with the logical flow being interrupted by off-hand remarks and description of one topic spread over different parts of the manuscript, sometimes repetitively. In a few cases, the text is burdened with statements of the obvious (e.g., "lipid structure is closely related to lipid function", "allows identification of lipid-associated terms in lipidomes"). There are multiple typos, grammar errors and misused words or terms that make a mere reading of the article a torture. One step to address this issues might be including subsections under the Findings section, another - careful reassessment of what material represents technical side and belongs to Methods and what should be in the Findings (my feeling is that a good portion of the LION description, currently under Methods, actually belongs to the Findings, right after the background information). The same goes to figure legends - I think currently they are overloaded with information that belongs in the Methods.
The manuscript suffers from frequent use of vague statements. Instead of describing WHAT was done the authors simply state the means for doing it: "we used" this or that, "we made use of" this or that, such and such "was used", etc. Instead of explaining HOW something was done a bare statement "based on" is often made. References are missing (e.g., "as described in the literature", p.4, l.53, "was reported", p.5, l.99). The tally of connections between membrane biophysics and cell biology (p.3, l.35-43) looks random and lacking completeness. Besides, it seems somewhat misplaced.
Authors use what appears to be in-house or jargon terms, such as "by target list", "by ranking" for the modes of the enrichment analysis, "local" statistics, etc. Use of such terms should be avoided. For such important terms as the modes of analysis the names should be related to their function and, ideally, self- explanatory (or, at least, thoroughly explained).
All these issues pertaining to the quality of the narration should be addressed before the substance of the work can be properly evaluated. However, even in the present state the manuscript allows to point out the following weaknesses/areas for improvement:

1. The LION should be completely verbally described (beyond the present reference to the .obo file). This should include a list of categorical ontology terms and rules of association between them. For the ones that are not obvious, a justification should be provided. As it stands now, the terms in question are hidden inside 1275-page long Excel file among about 50,000 terms representing individual lipids. Some of them relate to conventional structural elements of lipids, others are less obvious. For example, "fatty acid with 16-18 carbons" - is there any scientific meaning in this term? What is so special about this particular chain length? What exactly are the extra levels of classification between lipid classes and species? - they are mentioned but not described.
2. The enrichment tool is the crux of the article, the thing the authors are trying to "sell". However, there is no description of what it does and how it can be used. I flatter myself to be a qualified used but I could not make a head or tail of what the so called "by target list" mode does. If my "target list" includes unsaturated lipids I'll get enrichment in "double bonds", "below average transition temperature", etc. That much is obvious without running the tool. What else? What are the scenarios when I need to use it? Why do I need two lipidomic data sets for this? What does "derived from thresholding or clustering" mean? The second mode, apart from the name (why "by ranking"? isn't this purely technical approach to facilitate stat analysis?), is less problematic. However, the option to limit analysis to a specific set of terms ("terms of interest") should be mentioned upfront. Then, the questions arise in what scenarios this would be advantageous? Would this create a bias in the analysis or not, both with regard to outcome and its stat significance?
3. The claim of the scope is overreaching. The "function" category, most interesting for cell biology researchers, appears to be extremely frugal, limited just to the crudest distinction between structural, signaling and storage functions. If this perception is correct, the LION would be of limited value for cell biology. The "chemical" properties appear to be a misnomer with chemical information limited purely to structural elements with no regard to reactivity, biochemical synthetic pathways, etc. I would say that, according to this Technical Note, the LION is the ontology linking lipidomics data to biophysical properties of corresponding membranes. The testing of the ontology was performed in a set of assays pertaining to membrane biophysics.
4. It would be advantageous to sync terminology with other ontologies whenever possible, for example, use the GO term "cellular component" instead of "cellular localization", etc. "Lipid component" is a very dubious term for a structural lipid.
5. The biophysical properties of the vast majority of lipids were inferred from a limited set of literature data. It is therefore of utmost importance to thoroughly describe the approach used. What kind of data the sources provided? Where they for individual lipids or mixes, measured or calculated? How many entries? The equations for the multiple linear (sic!) regression analysis should be shown. The resulting coefficients could be of value by itself - why not publish them here?
6. The lipids appear to be divided into "quintiles" using a hard-to-describe (and almost lacking description in the manuscript) procedure based initially on a number of lipids in each group rather than the value of a biophysical parameter. What is the rationale for this? Does transition temperature of a lipid membrane care how many other membranes share the same value? I think the categorization should be based upon the magnitudes of biophysical properties alone. By the way, how many groups are actually there? The text says 5 but Fig. 2 shows 7… Also, Fig. 2 shows FDR q-values which are not mentioned in either legend or the main text.

7. It is not absolutely clear from the manuscript but appears that the enrichment tool relies on the significance of the changes (p-value), as opposed to magnitude, to evaluate enrichment. Is this true? Is it possible that highly significant changes in low abundance lipids would dominate the outcome list without having much effect on the properties of membrane?
8. More detail should be provided on the statistics, for example, how the distribution curve was generated for K-S analysis, what were the input parameters for the Fisher exact test, etc.
9. Methods for PDA assay and LC-MS should be brought to compliance with editorial guidelines to allow duplicate these studies. Missing are parameters such as cell number, concentration of the dye, shape of LC gradient, LC system used, MS/MS settings, to name a few. The full name of the Fusion mass spec should be provided because there are several different models. The text is not clear on the sequence of events: it sounds as if analyte ions fly from orbitrap to linear ion trap for detection - is this even possible?
10. With regard to membrane fluidity data, although they show the desired differences they could be made much more convincing with appropriate controls subtracting intrinsic fluorescence of the cells.
11. Annotating lipids with the "most abundant fatty acid composition" is misleading - if isobaric species are not resolved the overall composition (total carbons, total double bonds) should be shown as primary annotation (possibly followed by the most abundant isomer).

Level of Interest

Please indicate how interesting you found the manuscript: Choose an item.

Quality of Written English

Please indicate the quality of language in the manuscript: Choose an item.

Declaration of Competing Interests

Please complete a declaration of competing interests, considering the following questions:

- Have you in the past five years received reimbursements, fees, funding, or salary from an organisation that may in any way gain or lose financially from the publication of this manuscript, either now or in the future?
- Do you hold any stocks or shares in an organisation that may in any way gain or lose financially from the publication of this manuscript, either now or in the future?
- Do you hold or are you currently applying for any patents relating to the content of the manuscript?
- Have you received reimbursements, fees, funding, or salary from an organization that holds or has applied for patents relating to the content of the manuscript?
- Do you have any other financial competing interests?
- Do you have any non-financial competing interests in relation to this paper?

If you can answer no to all of the above, write 'I declare that I have no competing interests' below. If your reply is yes to any, please give details below.

I declare that I have no competing interests

I agree to the open peer review policy of the journal. I understand that my name will be included on my report to the authors and, if the manuscript is accepted for publication, my named report including any attachments I upload will be posted on the website along with the authors' responses. I agree for my report to be made available under an Open Access Creative Commons CC-BY license (http://creativecommons.org/licenses/by/4.0/). I understand that any comments which I do not wish to be included in my named report can be included as confidential comments to the editors, which will not be published.

I agree to the open peer review policy of the journal

To further support our reviewers, we have joined with Publons, where you can gain additional credit to further highlight your hard work (see: https://publons.com/journal/530/gigascience). On publication of this paper, your review will be automatically added to Publons, you can then choose whether or not to claim your Publons credit. I understand this statement.

Yes Choose an item.
